# Hepatocyte BDNF Acts as a Novel Immune Checkpoint to Restrain TLR4‐Mediated Acute Hepatitis

**DOI:** 10.1002/advs.202521164

**Published:** 2026-03-25

**Authors:** Weiwei Zhu, Yaqian Cui, Yongqiang Zhou, Yihui Zheng, Lijiang Huang, Leiming Jin, Qianhui Zhang, Pan Chen, Mengsha Lin, Jiaxi Ye, Yongqiang Xiong, Joo Young Huh, Xiang Hu, Xiaokun Li, Wu Luo, Guang Liang

**Affiliations:** ^1^ Department of Cardiology and Medical Research Center the First Affiliated Hospital of Wenzhou Medical University Wenzhou Zhejiang China; ^2^ Affiliated Cangnan Hospital and Chemical Biology Research Center Wenzhou Medical University Wenzhou Zhejiang China; ^3^ School of Pharmaceutical Science Wenzhou Medical University Wenzhou Zhejiang China; ^4^ Department of Chemoradiation Oncology the First Affiliated Hospital of Wenzhou Medical University Wenzhou Zhejiang China; ^5^ The Second Affiliated Hospital and Yuying Children's Hospital of Wenzhou Medical University Wenzhou Zhejiang China; ^6^ Joint Research Center on Medicine the Affiliated Xiangshan Hospital of Wenzhou Medical University Ningbo Zhejiang China; ^7^ College of Pharmacy Chung‐Ang University Seoul Republic of Korea; ^8^ School of Pharmaceutical Sciences Hangzhou Medical College Hangzhou Zhejiang China

**Keywords:** Acute hepatitis, Acute liver failure, BDNF, BDNF‐mimetic peptide, TLR4

## Abstract

Acute hepatitis is a major pathological process underlying acute liver injury (ALI) and acute liver failure (ALF), both of which are associated with high mortality. Yet, no effective treatment is currently available, underscoring the pressing need for novel therapeutic targets. By integrating multiple transcriptomic datasets, this study finds that the expression of brain‐derived neurotrophic factor (BDNF) is consistently downregulated in hepatocytes across various ALI/ALF models. Mechanistically, this downregulation is attributed to transcriptional repression of BDNF by RE1‐silencing transcription factor. Restoration of endogenous BDNF or exogenous administration of recombinant BDNF significantly alleviates LPS/DGal‐induced ALI/ALF. Correlation analysis and proteomic profiling reveal that BDNF exerts potent anti‐inflammatory effects by directly binding to and antagonizing Toll‐like receptor 4 (TLR4) on macrophages. Structural analysis identifies amino acids 233–244 of BDNF as the key functional domain responsible for this effect. A synthetic 12‐mer peptide derived from this region, termed BDP12, retains TLR4‐antagonizing ability, demonstrating strong anti‐inflammatory efficacy and a favorable safety profile in cultured macrophages and mouse ALI/ALF models. In conclusion, this study identifies hepatocyte‐derived BDNF as an endogenous antagonist of TLR4 and a critical immune checkpoint in acute hepatitis. BDNF and its mimetic peptide BDP12 represent promising therapeutic candidates for treating acute hepatitis‐mediated ALI/ALF.

## Graphical abstract

This study identifies hepatocyte‐derived BDNF as an endogenous TLR4 antagonist that alleviates acute hepatitis. BDNF is downregulated in hepatocytes via REST‐mediated transcriptional repression during ALI/ALF. Mechanistically, BDNF binds to TLR4 on macrophages to suppress inflammation. The functional domain (aa 233244) was used to develop a synthetic mimetic peptide, BDP12, which retains TLR4 antagonism and shows potent anti‐inflammatory efficacy and safety in vitro and in vivo, thus establishing BDP12 as a promising therapeutic candidate for acute hepatitis.

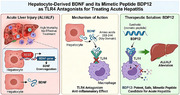

**Abbreviations**
ALF: acute liver failure
ALI: acute liver injury
ALT: alanine aminotransferase
AST: aspartate aminotransferase
BDNF: brain‐derived neurotrophic factor
BDP12: BDNF‐mimetic dodecapeptide
CLP: cecal ligation and puncture
DAMP: damage‐associated molecular pattern
DGal: d‐galactosamine
HMGB1: high mobility group box 1
ICAM1: intercellular adhesion molecule 1
IFN‐α: interferon‐alpha
IL‐6: interleukin‐6
IRF3: interferon regulatory factor 3
LPS: lipopolysaccharide
MLKO: myeloid lineage‐specific knockout
MPO: myeloperoxidase
NF‐κB: nuclear factor kappa B
PLA: proximity ligation assay
REST: RE1‐silencing transcription factor
SPR: surface plasmon resonance
TAK1: TGF‐β activated kinase 1
TBK1: TANK‐binding kinase 1
TLR4: Toll‐like receptor 4
TNF‐α: tumor necrosis factor‐alpha
TrkB: tropomyosin receptor kinase B
VCAM1: vascular cell adhesion molecule 1

## 1 Introduction

Acute hepatitis, which can be triggered by microbial infections (e.g., hepatitis virus, bacteria) [1, 2], drugs (e.g., acetaminophen, methotrexate) [3], alcohol [4], or autoimmune responses [5], is characterized by complex immune activation and hepatocellular injury. Especially, pathogen‐associated molecular patterns (PAMPs) from viruses or bacteria engage pattern recognition receptors (PRRs), such as Toll‐like receptors (TLRs; e.g., TLR2/3/4/7/9) and RIG‐I‐like receptors, activating downstream NF‐κB and IRF3 signaling pathways in hepatic macrophages [6, 7]. This initiates a pro‐inflammatory cascade, including the release of inflammatory factors and the recruitment of cytotoxic T lymphocytes, ultimately leading to hepatocyte injury [8]. Damage‐associated molecular patterns (DAMPs), such as high mobility group box‐1 protein (HMGB1), are subsequently released, also activating TLR4 and NLRP3 inflammasomes to amplify inflammatory responses [9]. Although mild cases of acute hepatitis are often self‐limiting, moderate to severe forms frequently progress to acute liver injury and failure (ALI/ALF), which carries mortality rates of 50%–75% [10]. Patients with preexisting chronic liver diseases, such as metabolic dysfunction‐associated steatotic liver disease (MASLD), exhibit diminished hepatic resilience, predisposing them to acute‐on‐chronic liver failure (ACLF) and worse clinical outcomes [11]. Current therapeutic strategies, including antimicrobials and N‐acetylcysteine (NAC) [12, 13], primarily target etiological factors but lack precision in attenuating the inflammatory storm, thereby limiting their clinical applicability and highlighting the urgent need for novel immune‐based therapies.

Brain‐derived neurotrophic factor (BDNF), a member of the neurotrophin family firstly identified in 1982 and exerts its effects via the high‐affinity tropomyosin receptor kinase B (TrkB) in the brain [14]. BDNF plays essential roles in neuronal development and function, with BDNF knockout mice exhibiting early postnatal lethality due to severe neurodevelopmental defects [15]. Beyond its well‐established regulatory role in the central nervous system (CNS), peripheral BDNF also exerts important physiological functions in various non‐neural organs. Accumulating evidence suggests that BDNF participates in the protection and repair of peripheral tissues through diverse mechanisms. For example, a previous study has shown that BDNF significantly attenuates myocardial ischemia‐reperfusion injury, primarily by enhancing cardiomyocyte survival and myocardial contractility [16]. In the respiratory system, BDNF has been shown to promote alveolar epithelial regeneration and repair following lung injury, representing a potential strategy for destructive lung diseases [17]. Notably, BDNF also functions as a key myokine, reprogramming lipid metabolism through activation of the PPARδ signaling pathway and regulating mitochondrial fission and quality control to maintain metabolic homeostasis in skeletal muscle [18, 19]. Despite these advances, the roles of hepatic BDNF in liver diseases remain poorly understood. A recent study showed that whole‐body BDNF knockdown mice spontaneously developed non‐alcoholic steatohepatitis [20]. Paradoxically, another study found that hepatocyte‐specific BDNF knockout alleviated diet‐induced hyperglycemia and dyslipidemia in mice [21]. These contradictions underscore the need to elucidate the context‐dependent mechanisms of BDNF in the liver.

In the present study, using well‐established models of lipopolysaccharide (LPS)/d‐galactosamine (DGal)‐induced ALI/ALF, we identified hepatic BDNF consistently downregulated across multiple injury paradigms. We found that hepatocyte‐derived BDNF protected mouse livers against acute hepatitis and ALI/ALF. Mechanistically, BDNF directly binds to TLR4 and then antagonizes TLR4 signaling on macrophages, attenuating pro‐inflammatory cascades. Guided by structural simulation into the BDNF‐TLR4 interaction interface, we developed BDP12, a peptidyl BDNF‐mimetic that potently inhibits TLR4 activation. BDP12 also demonstrated therapeutic efficacy across multiple models of ALI/ALF, including LPS/D‐Gal‐, sepsis‐, and autoimmune‐induced injury. Collectively, our findings establish hepatocyte‐derived BDNF as a new endogenous antagonist of TLR4 and provide a rationale for finding new therapeutic strategies for ALI/ALF.

## 2 Results

### 2.1 Hepatocyte BDNF Expression Was Downregulated in the Progression of Acute Liver Injury and Failure

**FIGURE 1:**
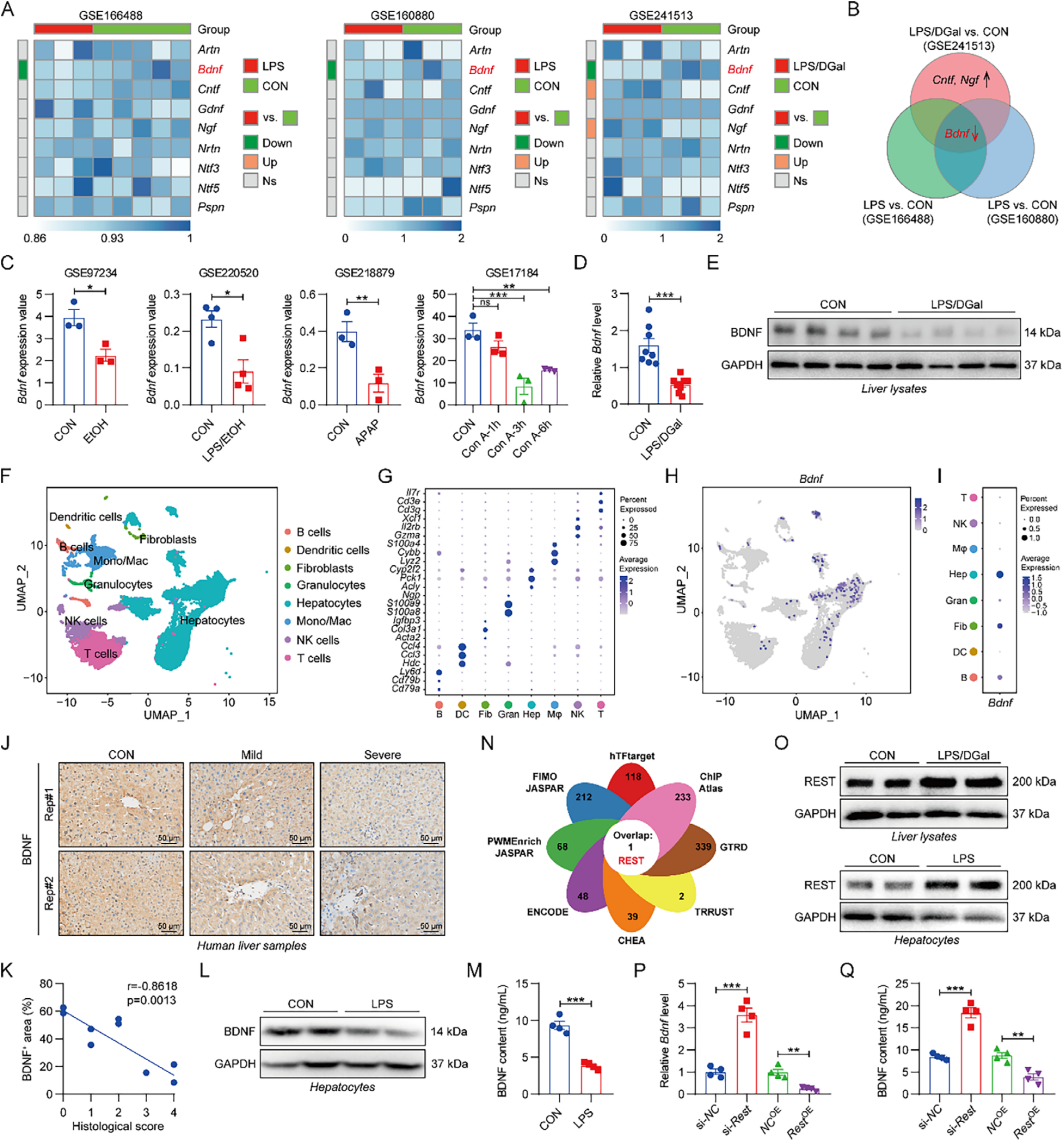
Hepatocyte BDNF expression was downregulated in the progression of acute liver injury and failure. (A) Heatmap of representative neurotrophic factors (NTKs), including *Artn*, *Bdnf*, *Cntf*, *Gdnf*, *Ngf*, *Nrtn*, *Ntf3*, *Ntf5*, and *Pspn*, derived from publicly available datasets (GSE166488, GSE160880, and GSE241513) of LPS‐ and LPS/DGal‐induced ALI/ALF in the GEO database. (B) Venn diagram showing the differentially expressed genes among the datasets presented in panel A. (C) Count/FPKM values of *Bdnf* from datasets GSE97234, GSE220520, GSE218879, and GSE17184 across various models of ALI/ALF induced by EtOH, LPS/EtOH, APAP, and Con A. (D) mRNA levels of *Bdnf* in liver tissues from control and LPS/DGal‐treated mice, detected by RT‐qPCR. (E) Protein expression levels of BDNF in liver tissues from control and LPS/DGal‐treated mice, with GAPDH as the loading control. (F) UMAP plot showing eight cell clusters in the mouse liver. (G) Dot plot displaying the expression of marker genes for the eight liver cell clusters. (H) Feature plot illustrating the single‐cell expression distribution of *Bdnf* in the mouse liver. (I) Dot plot showing *Bdnf* expression across the eight liver cell clusters. (J) Immunohistochemical staining of BDNF in human liver tissues from non‐hepatitis and hepatitis individuals with no, mild, or severe injury. (K) Correlation analysis between BDNF‐positive areas and histological scores in human liver samples. Hepatocytes were treated with or without LPS (500 ng/mL for 24 h), and intracellular BDNF protein levels (L) and secreted BDNF levels (M) were detected. (N) Venn diagram summarizing the transcription factors predicted to regulate *Bdnf* using eight independent databases. (O) Protein expression of REST in liver tissues from control and LPS/DGal‐treated mice, and in hepatocytes treated with or without LPS (500 ng/mL for 24 h), with GAPDH as the loading control. (P,Q) Primary hepatocytes were transfected with *Rest* siRNA or *Rest* overexpression plasmid for 24 h. mRNA levels of *Bdnf* were measured by RT‐qPCR (P), and secreted BDNF levels in the medium were assessed by ELISA (Q). Data are presented as mean ± SEM; *n* = 8 for panel D and *n* = 4 for panels M–Q; ns = not significant; *p < 0.05; **p < 0.01; ***p < 0.001.

We first investigated transcriptional changes of neurotrophic factor (NTF) family members in the liver tissues across two sepsis‐induced and one LPS/DGal‐induced models of ALI/ALF. Among the nine classical NTFs, *Bdnf* emerged as the only one consistently and significantly downregulated across all three RNA‐seq datasets (Figure 1A,B). Hepatic downregulation of *Bdnf* was also observed in multiple ALI/ALF models induced by diverse triggers, including alcohol, alcohol/LPS, acetaminophen (APAP), and concanavalin A (Con A) (Figure 1C). We further confirmed this downregulation at both mRNA and protein levels in the LPS/DGal‐induced model (Figure 1D,E and S1A). To address the cellular origin of hepatic BDNF, we employed single‐cell RNA sequencing to map *Bdnf* expression across liver cell types. Based on 24 canonical markers, eight major liver cell populations were identified, among which *Bdnf* was predominantly expressed in parenchymal hepatocytes (Figure 1F–I). Consistently, immunoblotting of isolated primary hepatocytes, primary Kupffer cells, and primary hepatic stellate cells further confirmed that BDNF expression was largely restricted to hepatocytes rather than nonparenchymal cells (Figure S1B). These findings were further corroborated by immunohistochemical and immunofluorescence staining of both human and mouse liver tissues, which showed predominant BDNF localization in hepatocytes and a marked reduction in hepatocyte BDNF expression in patients with hepatitis and in LPS/DGal‐treated mice (Figure S1C–F). Notably, in human hepatitis samples, hepatocyte BDNF expression exhibited a significant inverse correlation with disease severity (Figure 1J,K). In vitro, LPS stimulation at a noncytotoxic dose in primary hepatocytes also led to reduced intracellular BDNF levels and diminished BDNF secretion into the culture medium (Figures 1L,M and S1G).

To elucidate the underlying mechanism of LPS‐induced BDNF suppression, we performed an integrative analysis of eight transcription factor (TF) prediction databases and identified RE1‐silencing transcription factor (REST) as the only high‐confidence upstream regulator (Figure 1N). REST represses transcription by binding to neuron‐restrictive silencer elements (NRSE) through its zinc‐finger domains, thereby silencing neuronal genes in non‐neuronal tissues [22]. Western blot analysis revealed that REST expression was significantly upregulated in both LPS‐treated hepatocytes and LPS/DGal‐challenged liver tissues (Figures 1O and S1H). Functional modulation of REST in hepatocytes showed that REST knockdown elevated BDNF transcription and secretion, whereas REST overexpression suppressed both (Figures 1P,Q and S1I). Collectively, these findings demonstrate that REST‐mediated BDNF suppression in hepatocytes may be involved in the pathogenesis of ALI/ALF.

### 2.2 Hepatocyte‐Derived BDNF Attenuates Acute Liver Injury and Failure

**FIGURE 2:**
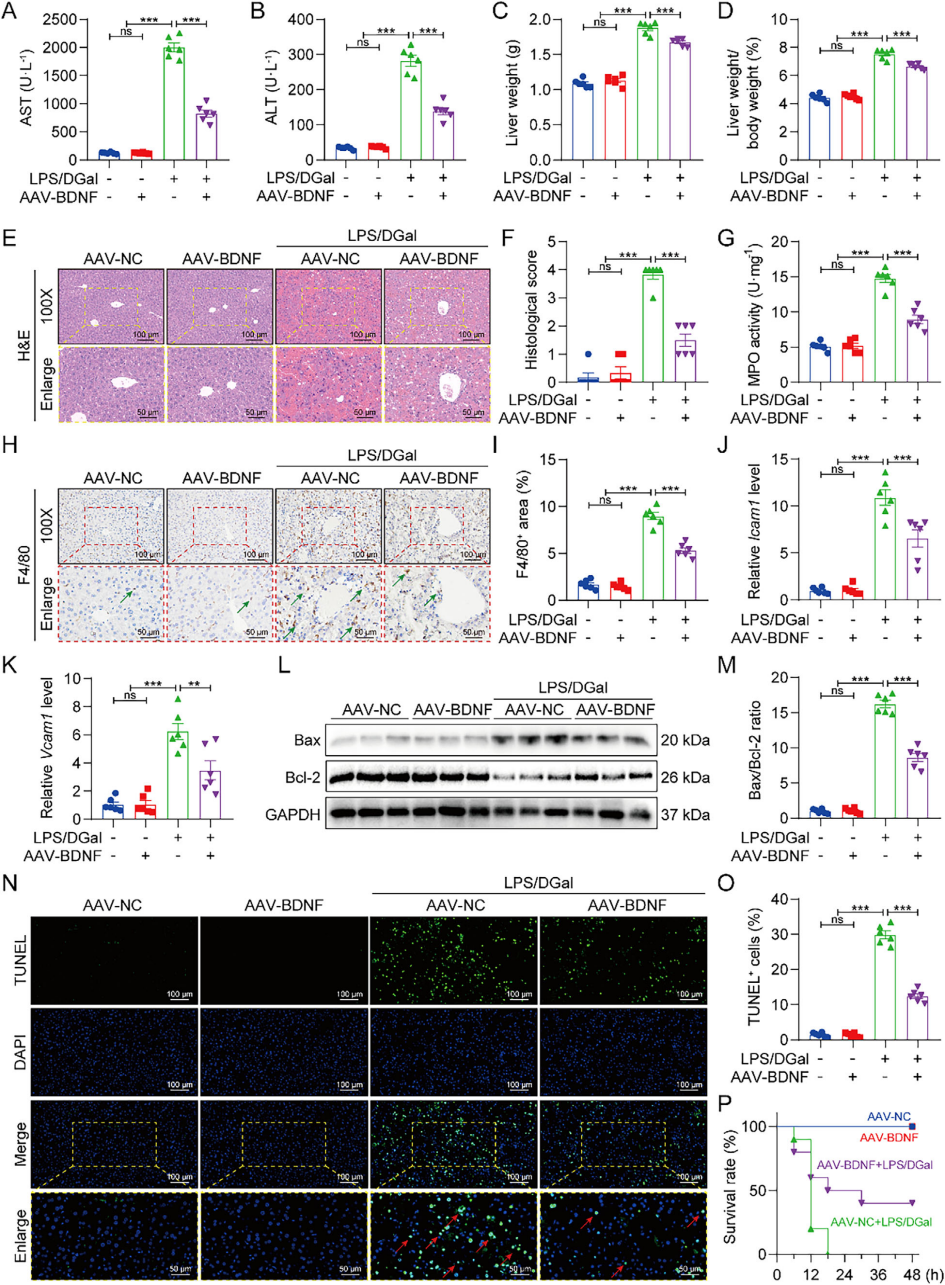
Hepatocyte‐derived BDNF attenuates acute liver injury and failure. Hepatocyte‐specific BDNF overexpression and secretion were achieved via tail vein injection of AAV8‐TBG‐sp‐BDNF (Ad‐BDNF) for 2 weeks, while AAV8‐TBG‐sp‐NC (Ad‐NC) served as the negative control. ALI/ALF was induced using LPS combined with DGal. Serum levels of AST (A) and ALT (B). Liver weight (C) and its ratio to body weight (D). (E) Representative H&E‐stained images of the liver section. (F) Quantification of hepatic histological scores for panel E. (G) MPO activity levels in liver lysates measured by a commercial kit. (H) Immunohistochemistry staining of F4/80 in liver tissues. (I) Quantification of F4/80‐positive areas shown in panel H. mRNA levels of adhesion molecules *Icam1* (J) and *Vcam1* (K) in liver tissues. (L) Protein expression of pro‐apoptotic Bax and anti‐apoptotic Bcl‐2 in liver tissues, with GAPDH as the loading control. (M) Bax/Bcl‐2 ratio calculated from densitometric quantification of blot in panel L. (N) Representative images of TUNEL staining in liver sections. TUNEL‐positive nuclei (green) indicate apoptotic cells. Nuclei were counterstained with DAPI. (O) Quantification of TUNEL‐positive cells shown in panel N. (P) Survival curve of Ad‐NC‐ or Ad‐BDNF‐infected mice subjected to LPS/DGal‐induced lethal model. Data are represented as mean ± SEM; *n* = 6 per group (panel P, n = 10); ns = not significant; *p < 0.05; **p < 0.01; ***p < 0.001.

To elucidate the specific role of hepatocyte BDNF in ALI/ALF, we first employed an adeno‐associated virus (AAV) vector to specifically overexpress the secreted form of mature BDNF in hepatocytes (AAV‐BDNF) (Figure S2A–C). In the LPS/DGal‐induced ALI/ALF model, a rapid and marked elevation in serum AST and ALT levels was observed, indicating severe hepatic injury (Figure 2A,B). This was accompanied by substantial liver edema, reflected by an approximately 60% increase in liver weight and liver‐to‐body weight ratio (Figure 2C,D). Notably, hepatocyte‐specific BDNF overexpression by AAV‐BDNF significantly ameliorated these injury markers (Figure 2A–D). Histological analysis revealed that AAV‐BDNF robustly attenuated LPS/DGal‐induced hepatic sinusoidal congestion, hepatocellular death, and other histopathological changes (Figure 2E,F). Further assessments, including myeloperoxidase (MPO) activity for inflammation condition (Figure 2G), F4/80 immunohistochemistry for macrophage infiltration (Figure 2H,I), and mRNA expression of adhesion molecules *Icam1* and *Vcam1* (Figure 2J,K), collectively demonstrated intense immune cell recruitment in ALI/ALF, which was also markedly alleviated by hepatic BDNF overexpression. At the 6‐h timepoint of LPS/DGal administration, hepatocyte‐specific BDNF overexpression also led to a reduced Bax/Bcl2 ratio (Figure 2L,M) and fewer TUNEL‐positive cells (Figure 2N,O), indicating AAV‐BDNF's protection against hepatocyte death. Collectively, these findings indicate that hepatocyte‐derived BDNF exerts broad protective effects in ALI/ALF, including attenuation of edema, hemorrhage, immune infiltration, and cell death, aligning with increased survival rates in hepatocyte‐specific BDNF‐overexpressing mice (Figure 2P).

**FIGURE 3:**
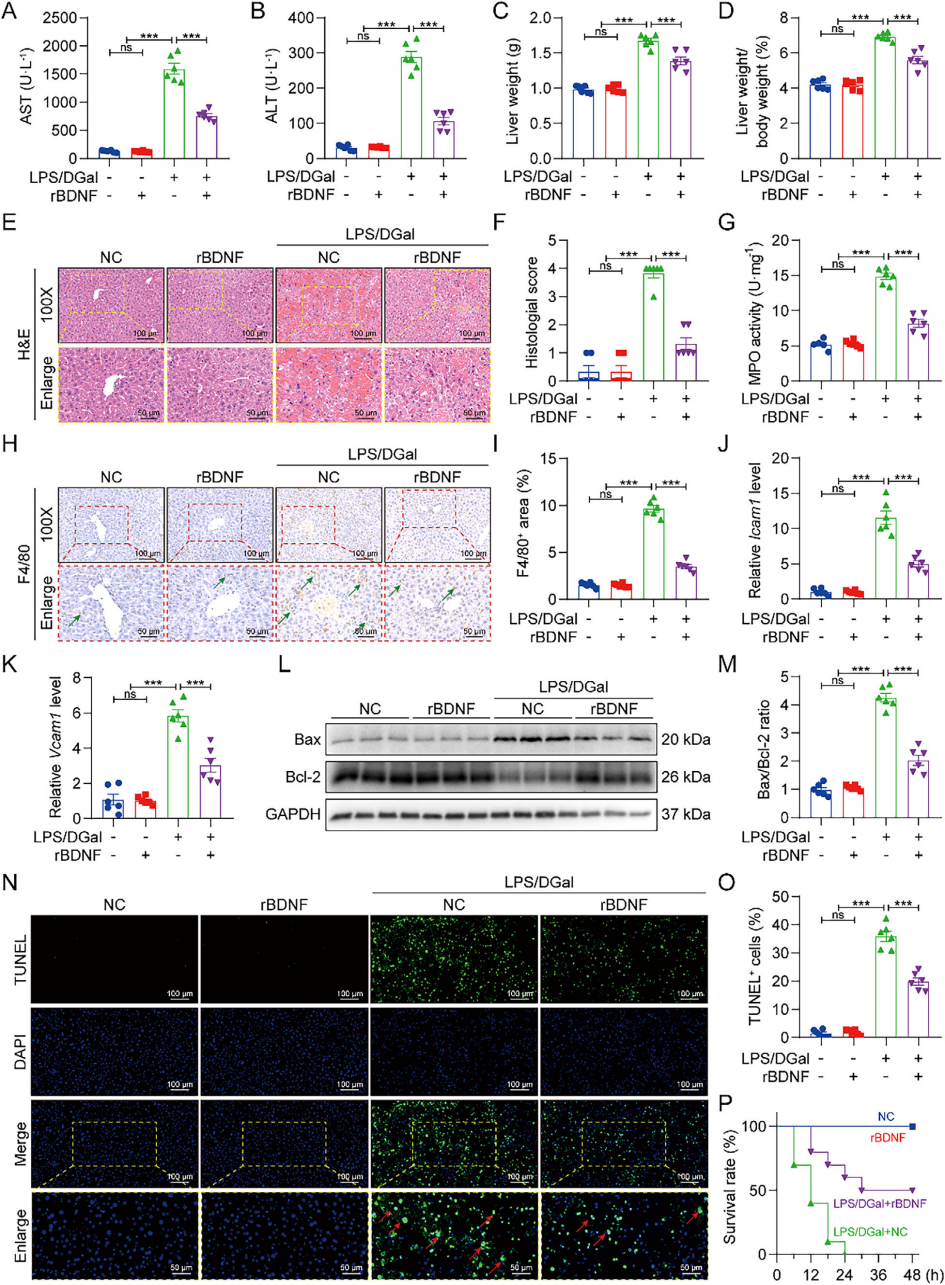
Recombinant BDNF protein alleviates acute liver injury and failure. Recombinant BDNF protein (rBDNF) or vehicle control was administered to mice via tail vein injection following LPS/D‐Gal treatment. Serum levels of AST (A) and ALT (B). Liver weight (C) and its ratio to body weight (D). (E) Representative H&E‐stained images of the liver section. (F) Quantification of hepatic histological scores for panel E. (G) MPO activity levels in liver lysates measured by a commercial kit. (H) Immunohistochemistry staining of F4/80 in liver tissues. (I) Quantification of F4/80‐positive areas shown in panel H. mRNA levels of adhesion molecules *Icam1* (J) and *Vcam1* (K) in liver tissues. (L) Protein expression of pro‐apoptotic Bax and anti‐apoptotic Bcl‐2 in liver tissues, with GAPDH as the loading control. (M) Bax/Bcl‐2 ratio calculated from densitometric quantification of blot in panel L. (N) Representative images of TUNEL staining in liver sections. TUNEL‐positive nuclei (green) indicate apoptotic cells. Nuclei were counterstained with DAPI. (O) Quantification of TUNEL‐positive cells shown in panel N. (P) Survival curve of NC‐ or rBDNF‐injected mice subjected to LPS/DGal‐induced lethal model. Data are represented as mean ± SEM; *n* = 6 per group (panel P, *n* = 10); ns = not significant; *p < 0.05; **p < 0.01; ***p < 0.001.

To increase the translational significance, we directly administered mice with recombinant BDNF protein (rBDNF) to rapidly elevate the BDNF level in the whole body, including blood and liver (Figure S2D–F). Compared to the LPS/DGal‐induced ALI/ALF mice treated with the vehicle, rBDNF administration similarly attenuated liver injury (Figure 3A,B), hepatic edema (Figure 3C,D), sinusoidal congestion (Figure 3E,F), inflammatory infiltration (Figure 3G–K), hepatocyte apoptosis (Figure 3L–O), and mortality (Figure 3P). These findings support a protective role of hepatocyte‐derived BDNF against LPS/DGal‐induced ALI/ALF.

### 2.3 Paracrine BDNF From Hepatocytes Alleviates Inflammation in Macrophages via NF‐κB Suppression

**FIGURE 4:**
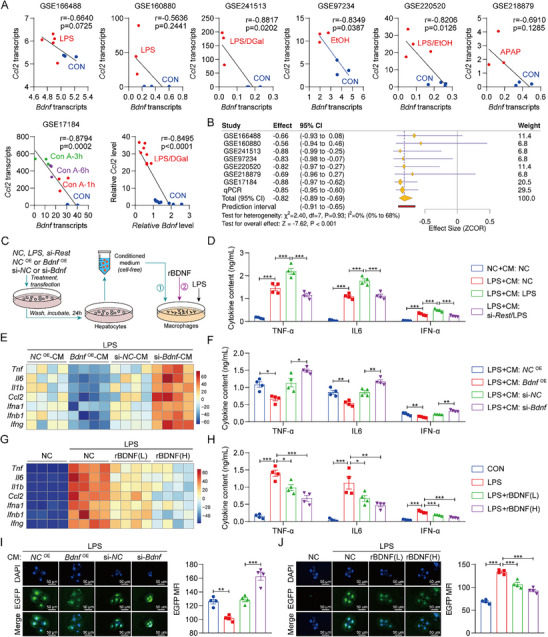
Paracrine BDNF from hepatocytes alleviates inflammation in macrophages via NF‐κB suppression. (A) Correlation analysis between *Bdnf* and the classical pro‐inflammatory chemokine *Ccl2* across multiple publicly available transcriptomic datasets of ALI/ALF induced by LPS, LPS/DGal, EtOH, LPS/EtOH, APAP, and Con A (including GSE166488, GSE160880, GSE241513, GSE97234, GSE220520, GSE218879, GSE17184), as well as in liver tissues from our LPS/DGal‐induced ALI/ALF mouse model. (B) A forest plot based on a random‐effects meta‐analysis model was generated to comprehensively evaluate the correlation between BDNF and CCL2 expression across eight independent datasets. (C) Schematic diagram illustrating the indirect co‐culture of macrophages with hepatocytes treated with or without LPS, subjected to *Rest* silencing, or to *Bdnf* silencing or overexpression. Alternatively, macrophages were directly treated with rBDNF. Macrophages were subsequently stimulated with LPS (500 ng/mL). (D) Macrophages were treated with hepatocyte CM for 0.5 h and then stimulated with LPS for 24 h. The concentrations of TNF‐α, IL‐6, and IFN‐α in the macrophage culture supernatant were measured by ELISA. (E) Hepatocyte CM was applied to macrophages for 0.5 h, followed by LPS stimulation for 12 h. Heatmap shows mRNA levels of several pro‐inflammatory genes, including *Tnf*, *Il6*, *Il1b*, *Ccl2*, *Ifna1*, *Ifnb1*, and *Ifng*. (F) Macrophages were treated with hepatocyte CM for 0.5 h and then stimulated with LPS for 24 h. The concentrations of TNF‐α, IL‐6, and IFN‐α in the macrophage culture supernatant were measured by ELISA. (G) rBDNF (50, 100 ng/mL) was applied to macrophages for 1 h, followed by LPS stimulation for 12 h. Heatmap shows mRNA levels of several pro‐inflammatory genes, including *Tnf*, *Il6*, *Il1b*, *Ccl2*, *Ifna1*, *Ifnb1*, and *Ifng*. (H) rBDNF (50, 100 ng/mL) was applied to macrophages for 1 h, followed by LPS stimulation for 24 h. The concentrations of TNF‐α, IL‐6, and IFN‐α in the macrophage culture supernatant were measured by ELISA. NF‐κB‐EGFP reporter cells were treated with hepatocyte CM for 2 h (I) or with rBDNF (50, 100 ng/mL) for 1 h (J), followed by stimulation with LPS for 24 h. EGFP fluorescence (green), indicating NF‐κB activation, was detected using fluorescence microscopy. Nuclei were counterstained with DAPI (blue). The mean fluorescence intensity (MFI) of EGFP was quantified using ImageJ and shown on the right. Data are represented as mean ± SEM; each dot represents data from an individual sample; ns = not significant; *p < 0.05; **p < 0.01; ***p < 0.001.

To investigate whether BDNF alleviates ALI/ALF by downregulating hepatic inflammation, we systematically analyzed the transcriptional correlation between BDNF and the classical inflammatory cytokine C‐C motif chemokine ligand 2 (CCL2) across various ALI/ALF models. In eight transcriptomic datasets induced by six different stimuli, *Bdnf* levels consistently exhibited a negative correlation with *Ccl2*, with five datasets showing statistically significant inverse correlations, suggesting that BDNF may act as a negative regulator of hepatic inflammation (Figure 4A). Importantly, random‐effects meta‐analysis of correlation coefficients demonstrated a strong and highly significant pooled inverse association between *Bdnf* and *Ccl2* expression (overall effect size = −0.82, 95% CI −0.89 to −0.69; *Z* = −7.62, *p* < 0.001) (Figure 4B). Between‐study heterogeneity was negligible (*I* ^2^ = 0%), indicating a high degree of consistency across diverse experimental models and stimuli (Figure 4B). The narrow prediction interval further supports that this negative correlation is likely to hold in future ALI/ALF datasets.

Given that infiltrated macrophages are key effector cells in liver inflammation [23], we employed an indirect hepatocyte‐macrophage co‐culture system (Figure 4C). Conditioned medium (CM) from LPS‐treated hepatocytes enhanced the inflammatory response of macrophages to LPS. Notably, this potentiation of inflammation was reversed when *Rest* was silenced in hepatocytes, indicating that the hepatocyte REST‐BDNF axis modulates macrophage inflammatory activation through intercellular communication (Figure 4D). Furthermore, conditioned media (CM) from hepatocytes with BDNF overexpression markedly suppressed macrophage inflammatory responses, as evidenced by significantly reduced transcription of proinflammatory cytokines (including *Tnf*, *Il6*, *Il1b*, *Ccl2*, *Ifna1*, *Ifnb1*, and *Ifng*), along with decreased secretion of cytokines (including TNF‐α, IL‐6, and IFN‐α) (Figure 4E‐F). Conversely, CM from low‐BDNF hepatocytes exacerbated the inflammatory response in macrophages (Figure 4E‐F). This anti‐inflammatory effect was further validated using rBDNF, which exerted dose‐dependent inhibition of LPS‐induced macrophage activation (Figure 4G,H). Furthermore, using an NF‐κB reporter system (EGFP‐tagged), we observed that high‐BDNF CM or rBDNF treatment significantly reduced NF‐κB activation in LPS‐induced challenged macrophages, while low‐BDNF CM enhanced it (Figure 4I,J). These data collectively demonstrate that hepatocyte‐derived BDNF is an endogenous anti‐inflammatory molecule in macrophages.

### 2.4 BDNF Is a Novel Antagonistic Ligand of TLR4 and Limits the TLR4 Inflammatory Signal

Extracellular BDNF secreted by hepatocytes may exert its anti‐inflammatory effects through binding to its transmembrane receptor in macrophages. Since TrkB has been reported as a receptor of BDNF in neuronal cells, we first employed ANA‐12, a specific inhibitor of TrkB, and found that ANA‐12 failed to affect the inhibition of BDNF on inflammatory responses in macrophages (Figure S3). These results suggest that the anti‐inflammatory activity of BDNF is TrkB‐independent and may involve an alternative, previously unidentified BDNF receptor in macrophages.

**FIGURE 5:**
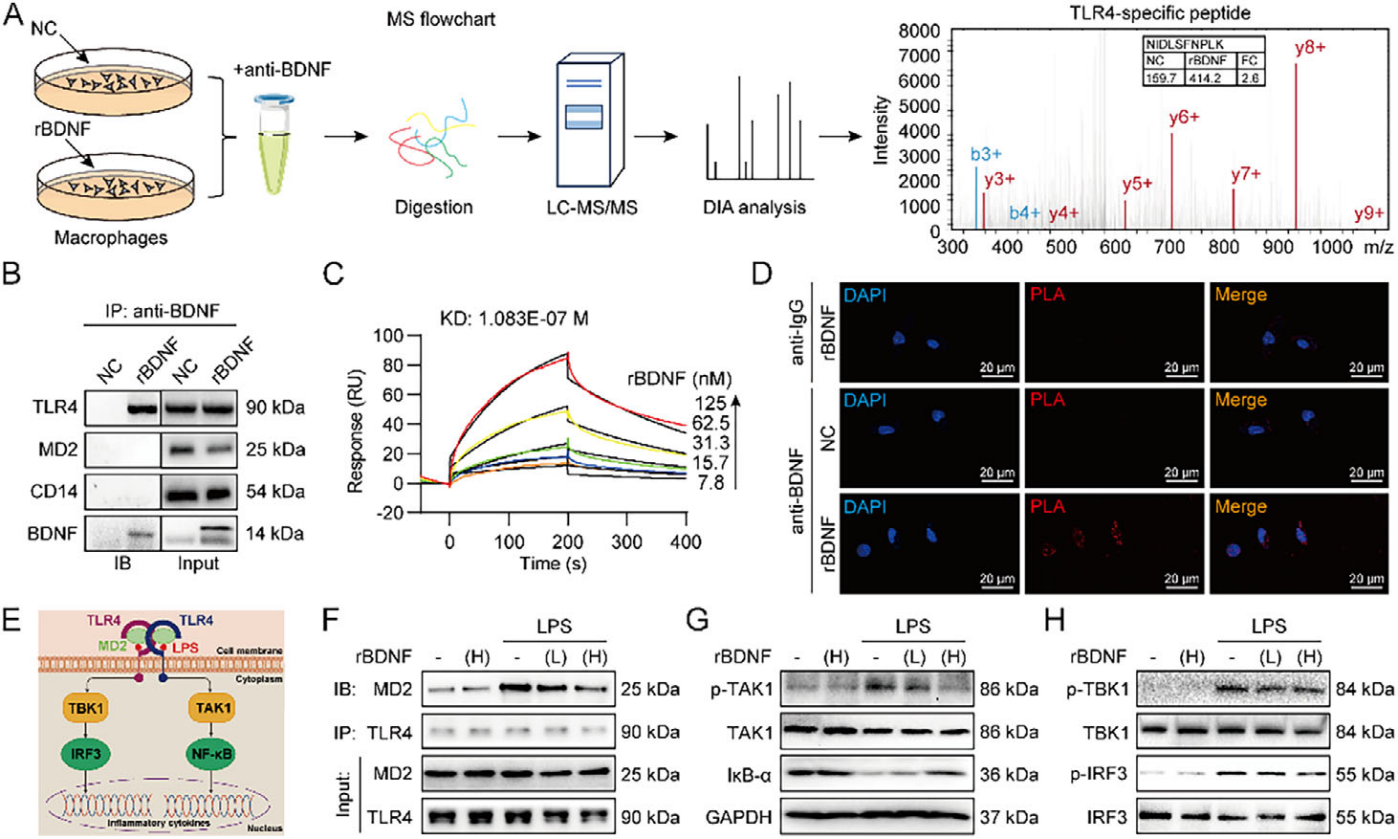
BDNF is a novel antagonistic ligand of TLR4 and limits the TLR4 inflammatory signal. (A) Schematic illustration of the IP‐MS/MS workflow used to identify membrane proteins interacting with BDNF. Macrophages were pretreated with recombinant BDNF (rBDNF, 100 ng/mL) or vehicle for 1 h, followed by cell lysis, anti‐BDNF immunoprecipitation, and peptide digestion. Peptides were analyzed by DIA‐based mass spectrometry. The mass spectrum of a representative TLR4‐derived unique peptide highly enriched in the rBDNF‐treated group is shown. (B) Co‐immunoprecipitation analysis of the interaction between BDNF and TLR4, as well as its co‐receptors MD2 and CD14, in macrophages pretreated with rBDNF (100 ng/mL) or vehicle for 1 h. (C) Surface plasmon resonance (SPR) analysis demonstrated direct binding between BDNF and the recombinant extracellular TIR domain of TLR4, with quantification of binding affinity. (D) In situ proximity ligation assay (PLA) demonstrating direct interaction between BDNF and TLR4 in macrophages pretreated with rBDNF (100 ng/mL) or vehicle for 1 h. PLA signals (red puncta) indicate protein‐protein interactions; nuclei are counterstained with DAPI (blue). IgG antibody was used as a negative control. (E) Schematic diagram of TLR4 signaling: LPS binds to the extracellular TLR4‐MD2 complex, triggering activation of intracellular TAK1‐NF‐κB and TBK1‐IRF3 pathways, leading to inflammatory responses. Immunoprecipitation and immunoblotting analyses showing that pretreatment of macrophages with rBDNF (50 or 100 ng/mL) for 1 h attenuates LPS (500 ng/mL, 0.5 h)‐induced formation of the MD2‐TLR4 complex (F), activation of the TAK1‐NF‐κB pathway (G), and activation of the TBK1‐IRF3 pathway (H).

We then used immunoprecipitation coupled with mass spectrometry to identify a new receptor of BDNF in macrophages. Interestingly, we identified the pattern recognition receptor TLR4, which is highly associated with acute hepatitis, as a binding partner of BDNF (Figure 5A and S4A). TLR4, a well‐established transmembrane receptor of the inflammatory pathway, was further validated to interact directly with BDNF in macrophages, which is independent of its canonical co‐receptors MD2 and CD14 (Figure 5B). Direct interaction between BDNF and TLR4 was confirmed by surface plasmon resonance (SPR), and their co‐localization in macrophages was further demonstrated using a proximity ligation assay (PLA), which yielded specific red fluorescent signals (Figure 5C,D). Under physiological conditions, TLR4 typically forms a heterodimer with MD2 via its extracellular domain to recognize inflammatory stimuli and subsequently activate the intracellular TAK1‐NF‐κB and TBK1‐IRF3 pathways (Figure 5E). However, our findings indicate that BDNF might occupy, in a spatially overlapping manner, the MD2‐binding site on TLR4, thereby competing with MD2 for TLR4 binding (Figure S4B). Consistently, treatment with rBDNF led to a marked dissociation of the MD2‐TLR4 complex (Figures 5F and S4C), accompanied by significant inhibition of downstream TAK1‐NF‐κB and TBK1‐IRF3 signaling (Figures 5G,H and S4D,E). These results establish TLR4 as a novel functional receptor for BDNF and demonstrate that BDNF exerts anti‐inflammatory effects by competitively binding to TLR4 and thereby antagonizing the TLR4‐mediated inflammatory cascade.

We also examined the in vivo effects of BDNF on the TLR4 signaling pathway. Administration of rBDNF led to an increase in hepatic BDNF‐TLR4 complex level and a decrease in MD2‐TLR4 complex formation (Figure S5A–C). This was accompanied by the suppression of downstream TAK1 and TBK1 pathway activation (Figure S5D–G), as well as a reduction in the transcription and secretion of pro‐inflammatory cytokines (Figure S5H,I). These findings support that, in mouse models of ALI/ALF, extracellular BDNF antagonizes TLR4 and limits the inflammatory signaling activation, thereby exerting anti‐inflammatory effects against hepatitis.

### 2.5 Myeloid‐Specific TLR4 Deletion Abolishes the Protective Effects of BDNF Against Acute hepatitis and Liver Injury/Failure

**FIGURE 6:**
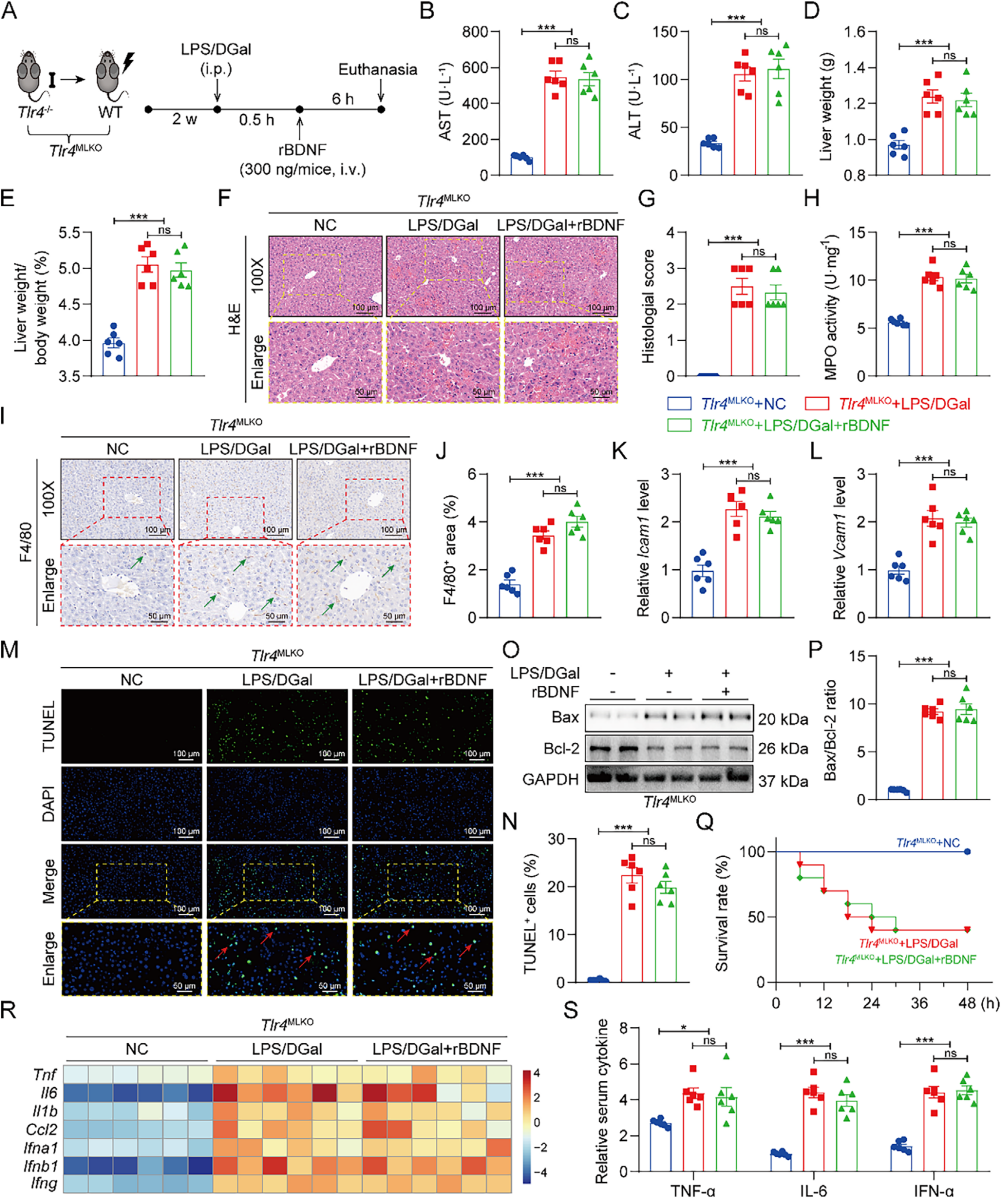
Myeloid‐specific TLR4 deletion abolishes the protective effects of BDNF against acute hepatitis and liver injury/failure. (A) Schematic illustration of the generation of myeloid lineage‐specific TLR4‐deficient mice (*Tlr4* ^MLKO^) through bone marrow transplantation, followed by LPS/DGal administration and rBDNF treatment. Serum levels of AST (B) and ALT (C). Liver weight (D) and its ratio to body weight (E). (F) Representative H&E‐stained images of the liver section. (G) Quantification of hepatic histological scores for panel F. (H) MPO activity levels in liver lysates measured by a commercial kit. (I) Immunohistochemistry staining of F4/80 in liver tissues. (J) Quantification of F4/80‐positive areas is shown in panel I. mRNA levels of adhesion molecules *Icam1* (K) and *Vcam1* (L) in liver tissues. (M) Representative images of TUNEL staining in liver sections. TUNEL‐positive nuclei (green) indicate apoptotic cells. Nuclei were counterstained with DAPI. (N) Quantification of TUNEL‐positive cells is shown in panel M. (O) Protein expression of pro‐apoptotic Bax and anti‐apoptotic Bcl‐2 in liver tissues, with GAPDH as the loading control. (P) Bax/Bcl‐2 ratio calculated from densitometric quantification of blot in panel O. (Q) Survival curve of myeloid‐specific TLR4‐deficient mice receiving either vehicle or rBDNF treatment in the LPS/DGal‐induced lethal model. (R) Heatmap showing mRNA levels of pro‐inflammatory factors *Tnf*, *Il6*, *Il1b*, *Ccl2*, *Ifna1*, *Ifnb1*, and *Ifng* in liver tissues. (S) Serum levels of TNF‐α, IL‐6, and IFN‐α in the ALI/ALF mouse model. Data are represented as mean ± SEM; *n* = 6 per group (panel Q, *n* = 10); ns = not significant; *p < 0.05; **p < 0.01; ***p < 0.001.

Single‐cell expression profiling of TLR4 in the liver revealed that TLR4 is predominantly expressed by monocytes, macrophages, and granulocytes, all of which are derived from myeloid lineage cells (Figure S6A–C). To determine whether BDNF exerts its anti‐hepatitis and protective effects against ALI/ALF through antagonizing TLR4 in myeloid cells, we generated myeloid lineage‐specific TLR4 knockout mice (TLR4^MLKO^) using bone marrow transplantation (Figure 6A). Following the LPS/DGal challenge, TLR4^MLKO^ mice exhibited a blunted damage response characterized by only moderate elevations in serum ALT and AST levels (Figure 6B,C) and an approximately 20% increase in liver weight (Figure 6D,E), indicating that TLR4 deficiency reduced the hepatic susceptibility to LPS/DGal‐induced injury. However, administration of rBDNF in these TLR4^MLKO^ mice failed to further improve liver injury or alleviate hepatic edema (Figure 6B–E). Consistently, multiple parameters, including histological liver hemorrhage assessed by H&E staining (Figure 6F,G), immune cell infiltration (Figure 6H–L), markers for hepatocyte apoptosis (Figure 6M–P), mouse survival rates (Figure 6Q), and expression and secretion of various inflammatory cytokines (Figure 6R,S), demonstrated enhanced tolerance to ALI/ALF in TLR4^MLKO^ mice. Notably, rBDNF treatment did not maintain its protective effects across these phenotypes in LPS/DGal‐challenged TLR4^MLKO^ mice (Figure 6F–S). These results validate that BDNF exerts its anti‐inflammatory and hepatoprotective effects through myeloid cell‐specific TLR4.

### 2.6 TLR4‐Targeting BDNF Mimetic Peptide Mitigates Inflammation Without TrkB‐Associated Proliferation

**FIGURE 7:**
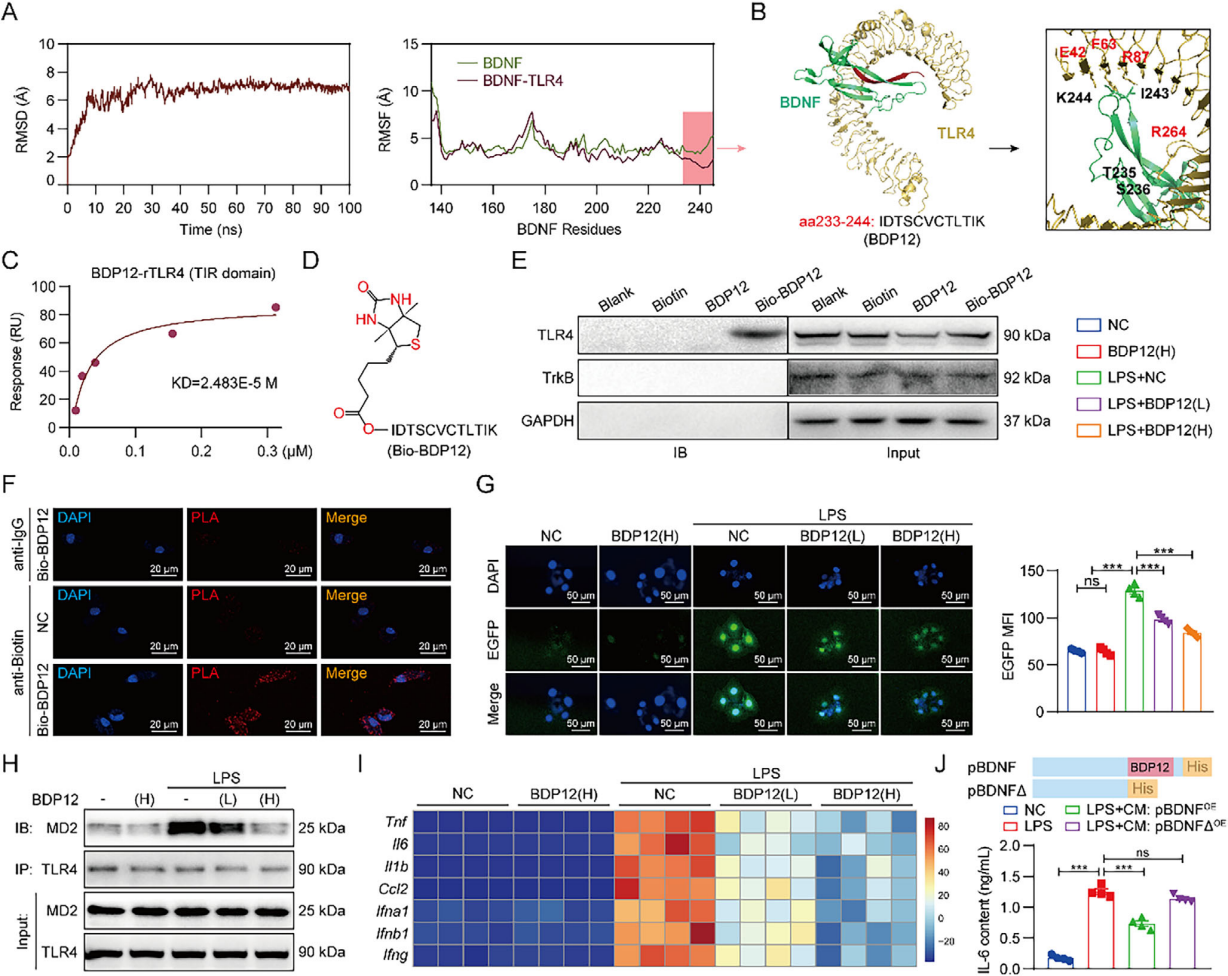
A TLR4‐targeting BDNF mimetic peptide mitigates inflammation. (A) Molecular dynamics (MD) simulations showed the root‐mean‐square deviation (RMSD) of the BDNF‐TLR4 complex over 100 ns. Root‐mean‐square fluctuation (RMSF) analysis demonstrated a pronounced reduction in RMSF at BDNF residues 233–244 upon complex formation compared with free BDNF. (B) Structural analysis of the BDNF‐TLR4 complex revealed that the BDNF residues (T235, S236, I243, and K244) and the TLR4 residues (E42, F63, R87, and R264) form intermolecular contacts within 3 Å at the binding interface. The amino acid sequence corresponding to BDNF residues 233–244 was synthesized as a BDNF‐mimetic peptide (BDP12). (C) Surface plasmon resonance (SPR) analysis demonstrated direct binding between BDP12 and the recombinant extracellular TIR domain of TLR4, with quantification of binding affinity. (D) Schematic representation of the N‐terminal biotinylated BDP12 (Bio‐BDP12). (E) Biotinylated pull‐down assays were performed to assess the interaction between BDP12 and TLR4 or TrkB. GAPDH was included as a negative control. (F) In situ proximity ligation assay (PLA) demonstrating direct interaction between BDP12 and TLR4 in macrophages pretreated with Bio‐BDP12 (4 µM) or vehicle for 1 h. PLA signals (red puncta) indicate polypeptide–protein interactions; nuclei are counterstained with DAPI (blue). IgG antibody was used as a negative control. (G) BDP12 (2, 4 µM) was applied to NF‐κB‐EGFP reporter cells for 1 h, followed by LPS stimulation for 24 h. EGFP fluorescence (green), indicating NF‐κB activation, was detected using fluorescence microscopy. Nuclei were counterstained with DAPI (blue). The mean fluorescence intensity (MFI) of EGFP was quantified using ImageJ and shown on the right. (H) Macrophages were pretreated with BDP12 (2, 4 µM) for 1 h, then stimulated with LPS (500 ng/mL) for 30 min. Immunoprecipitation was performed to assess MD2‐TLR4 complex formation. (I) Macrophages were pretreated with BDP12 (2, 4 µM) for 1 h, then stimulated with LPS (500 ng/mL) for 12 h. Heatmap shows mRNA levels of several pro‐inflammatory genes, including *Tnf*, *Il6*, *Il1b*, *Ccl2*, *Ifna1*, *Ifnb1*, and *Ifng*. (J) Hepatocytes were transfected with pBDNF or pBDNFΔ, respectively. Macrophages were then incubated with hepatocyte CM for 0.5 h and subsequently stimulated with LPS for 24 h. The concentration of IL‐6 in the macrophage culture supernatant was measured by ELISA. Data are represented as mean ± SEM; each dot represents data from an individual sample; ns = not significant; *p < 0.05; **p < 0.01; ***p < 0.001.

Our data demonstrated BDNF as an effective TLR4‐antagonist for treating inflammatory ALI/ALF. However, due to BDNF's strong activation of its canonical receptor TrkB, which may cause proliferative or oncogenic side effects [24], we tried to develop a more specific TLR4‐targeting strategy to maintain the anti‐inflammatory activity but avoid TrkB engagement. Using molecular docking and dynamics simulations, we found that the BDNF‐TLR4 interaction significantly altered the conformation of the BDNF 233–244 amino acid region, suggesting this segment as a key TLR4‐binding motif (Figure 7A). Further structural analysis revealed close intermolecular contacts within this region, particularly involving residues T235, S236, I243, and K244 (Figure 7B). Based on this finding, we synthesized a 12‐mer peptide (BDP12), corresponding to the 233–244 region of BDNF. Notably, molecular docking analysis further suggested that E42, F63, R87, and R264 residues of TLR4 may participate in the interaction with this BDNF segment (Figure 7B). Among these residues, E42, F63, and R87 are located in the N‐terminal leucine‐rich repeat (LRRNT) region of TLR4, a structural region important for maintaining the ectodomain configuration required for MD2 engagement [25]. Interaction with these residues may therefore interfere with the initial recognition process between TLR4 and MD2. In addition, R264 has been reported to contribute to ligand recognition during TLR4‐LPS binding, suggesting that interaction at this site may further affect ligand–receptor association [26]. Together, these results indicate that the 233–244 region of BDNF likely targets functionally important residues within the TLR4 ectodomain, potentially disrupting ligand recognition or adaptor engagement and thereby modulating TLR4 activation.

In parallel, we explored the structural basis of BDNF‐TrkB binding, where a tetrameric complex comprising a BDNF dimer and a TrkB dimer is formed. Using AlphaFold, we modeled the conformation of this complex, calculated the binding free energy of individual BDNF residues, and identified the central residues in BDNF‐TrkB interaction (Figure S7A,B). Interestingly, no significant interactions of TrkB with the BDNF aa 233–244 region were observed, suggesting that this segment may lack the capacity to bind TrkB.

Subsequently, SPR confirmed that BDP12 binds directly to the extracellular domain of TLR4 (Figure 7C). Biotin‐labeled pull‐down and proximity ligation assays demonstrated that BDP12 selectively binds to TLR4 in macrophages, whereas no detectable interaction was observed with TrkB or other potential BDNF‐interacting proteins, including CD9, TFRC, and TLR2, which were identified in Figure S4A (Figures 7D–F and S8A). This shared TLR4‐binding capacity enables BDP12 and BDNF to compete for TLR4; accordingly, as the concentration of one ligand, such as BDP12, increases, the binding of the other to TLR4 is competitively reduced (Figure S8B). Functionally, BDP12 also inhibited LPS‐induced MD2‐TLR4 complex formation, NF‐κB activation, and downstream inflammatory gene expression in both macrophages and NF‐κB reporter cells (Figures 7G–I and S8C). This inhibitory effect on inflammatory signaling may arise because BDP12, similar to BDNF, binds to TLR4 and sterically hinders MD2 association, or alternatively, because BDP12 binding induces conformational changes of TLR4 that reduce its affinity for MD2. Furthermore, deletion of the C‐terminal region of BDNF containing the BDP12 sequence (BDNFΔ) abolished the anti‐inflammatory activity of full‐length BDNF, underscoring the essential contribution of the BDP12 region to its anti‐inflammatory function (Figure 7J). Notably, BDP12 displayed even stronger anti‐inflammatory efficacy than full‐length BDNF, suppressing up to ∼80% of IL‐6 production, whereas BDNF achieved ∼50% inhibition in macrophages (Figure S8D). In addition to LPS induction, both BDNF and BDP12 alleviated macrophage inflammation induced by an endogenous TLR4 agonist HMGB1, validating BDNF/BDP12's TLR4‐antagonizing capability (Figure S8E). More importantly, BDP12 did not promote hepatocyte proliferation compared to BNDF. Treatment with rBDNF increased proliferating cell nuclear antigen (PCNA)‐positive hepatocytes in the mouse liver and promoted proliferation in cultured HepG2 cells, while BDP12 showed no such effect, indicating it avoids TrkB‐driven proliferative signaling (Figure S9A–C).

### 2.7 Therapeutic Potential of the BDNF‐Mimetic Peptide BDP12 Against Infection‐Related ALI/ALF

**FIGURE 8:**
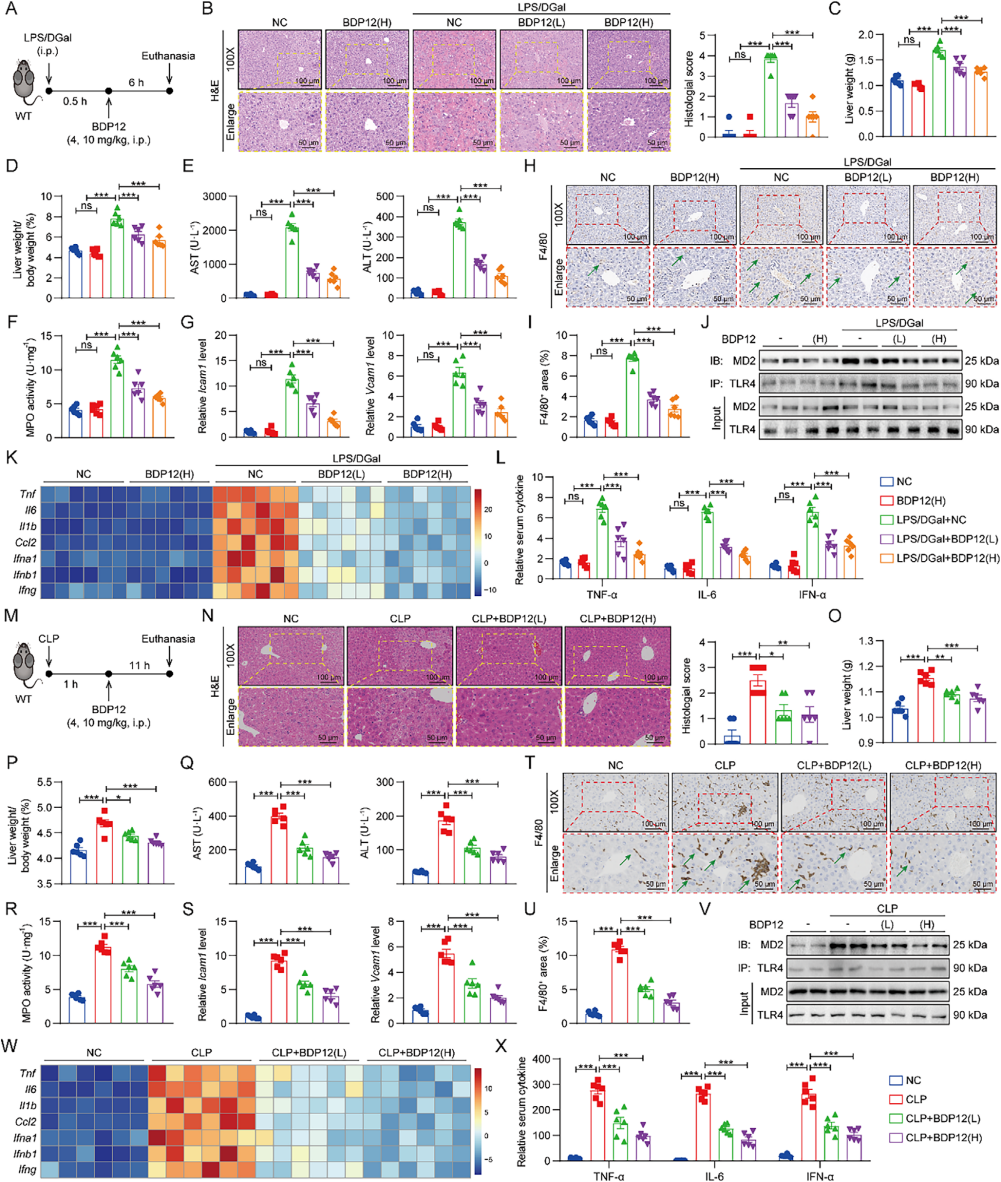
Therapeutic potential of the BDNF‐mimetic peptide BDP12 against infection‐related ALI/ALF. (A) Schematic diagram of the experimental workflow. Mice were treated with BDP12 or vehicle control via intraperitoneal injection following LPS/DGal challenge. (B) Representative H&E‐stained images of liver sections in mice from panel A. Quantification of hepatic histological scores is shown on the right. Liver weight (C) and its ratio to body weight (D) recorded of mice from panel A. (E) Serum levels of AST and ALT of mice from panel A. (F) MPO activity levels in liver lysates of mice from panel A. (G) mRNA levels of adhesion molecules *Icam1* and *Vcam1* in liver tissues of mice from panel A. (H) Immunohistochemistry staining of F4/80 in liver tissues of mice from panel A. (I) Quantification of F4/80‐positive areas shown in panel H. (J) Co‐immunoprecipitation analysis of MD2‐TLR4 complex formation in liver tissues of mice in panel A. (K) Heatmap showing mRNA levels of pro‐inflammatory factors *Tnf*, *Il6*, *Il1b*, *Ccl2*, *Ifna1*, *Ifnb1*, and *Ifng* in liver tissues of mice in panel A. (L) Serum levels of TNF‐α, IL‐6, and IFN‐α in the mice from panel A. (M) Schematic diagram of the experimental workflow. Mice were treated with BDP12 or vehicle control via intraperitoneal injection following cecal ligation and puncture (CLP) modeling. (N) Representative H&E‐stained images of liver sections in mice from panel M. Quantification of hepatic histological scores is shown on the right. Liver weight (O) and its ratio to body weight (P) recorded of mice from panel M. (Q) Serum levels of AST and ALT of mice from panel M. (R) MPO activity levels in liver lysates of mice from panel M. (S) mRNA levels of adhesion molecules *Icam1* and *Vcam1* in liver tissues of mice from panel M. (T) Immunohistochemistry staining of F4/80 in liver tissues of mice from panel M. (U) Quantification of F4/80‐positive areas shown in panel T. (V) Co‐immunoprecipitation analysis of MD2‐TLR4 complex formation in liver tissues of mice in panel M. (W) Heatmap showing mRNA levels of pro‐inflammatory factors *Tnf*, *Il6*, *Il1b*, *Ccl2*, *Ifna1*, *Ifnb1*, and *Ifng* in liver tissues of mice in panel M. (X) Serum levels of TNF‐α, IL‐6, and IFN‐α in the mice from panel M. Data are shown in mean ± SEM; *n* = 6; *p < 0.05; **p < 0.01; ***p < 0.001.

To investigate the therapeutic efficacy of BDP12 in ALI/ALF, we first employed the LPS/DGal‐induced model (Figure 8A). Consistent with previous observations, this model induced hepatic sinusoidal congestion (Figure 8B), a marked increase in liver weight (Figure 8C,D), and significantly increased serum ALT and AST levels (Figure 8E). BDP12 injection conferred a dose‐dependent hepatoprotective effect, as evidenced by the attenuation of these pathological indicators (Figure 8B–E). Histological and molecular analyses revealed a reduction in LPS/DGal‐induced immune cell infiltration, with marked decreases in the level of MPO (Figure 8F), the adhesion molecules *Vcam1* and *Icam1* (Figure 8G), and the macrophage marker F4/80 (Figure 8H,I). Furthermore, BDP12 treatment mitigated hepatocyte apoptosis (Figure S10A,B) and significantly improved survival outcomes in LPS/DGal‐challenged mice (Figure S10C). Mechanistically, BDP12 disrupted the formation of the MD2‐TLR4 complex (Figures 8J and S10D), resulting in downregulation of proinflammatory cytokine transcription and secretion (Figure 8K,L) in LPS/DGal‐challenged mouse liver tissues.

We further evaluated BDP12 in a CLP‐induced septic ALI/ALF model (Figure 8M). In this model, sepsis induced pericentral hepatocellular degeneration (Figure 8N) and modest hepatic edema (∼15%) (Figure 8O,P), accompanied by a 3‐ to 5‐fold increase in serum transaminases (Figure 8Q). BDP12 treatment also effectively reversed these injury markers (Figure 8N–Q) and reduced immune cell infiltration (Figure 8R–U). Although hepatocyte death in the sepsis model was less severe than that in the LPS/DGal model, BDP12 still conferred measurable protective effects (Figure S10E,F) and partially rescued sepsis‐induced mortality (Figure S10G). Importantly, BDP12 administration in this model also disrupted MD2‐TLR4 complex assembly (Figures 8V and S10H) and potently suppressed the expression of downstream inflammatory mediators in mouse livers (Figure 8W,X).

### 2.8 BDP12 Exhibits Advancing Therapeutic Efficacy in Noninfectious ALI/ALF

**FIGURE 9:**
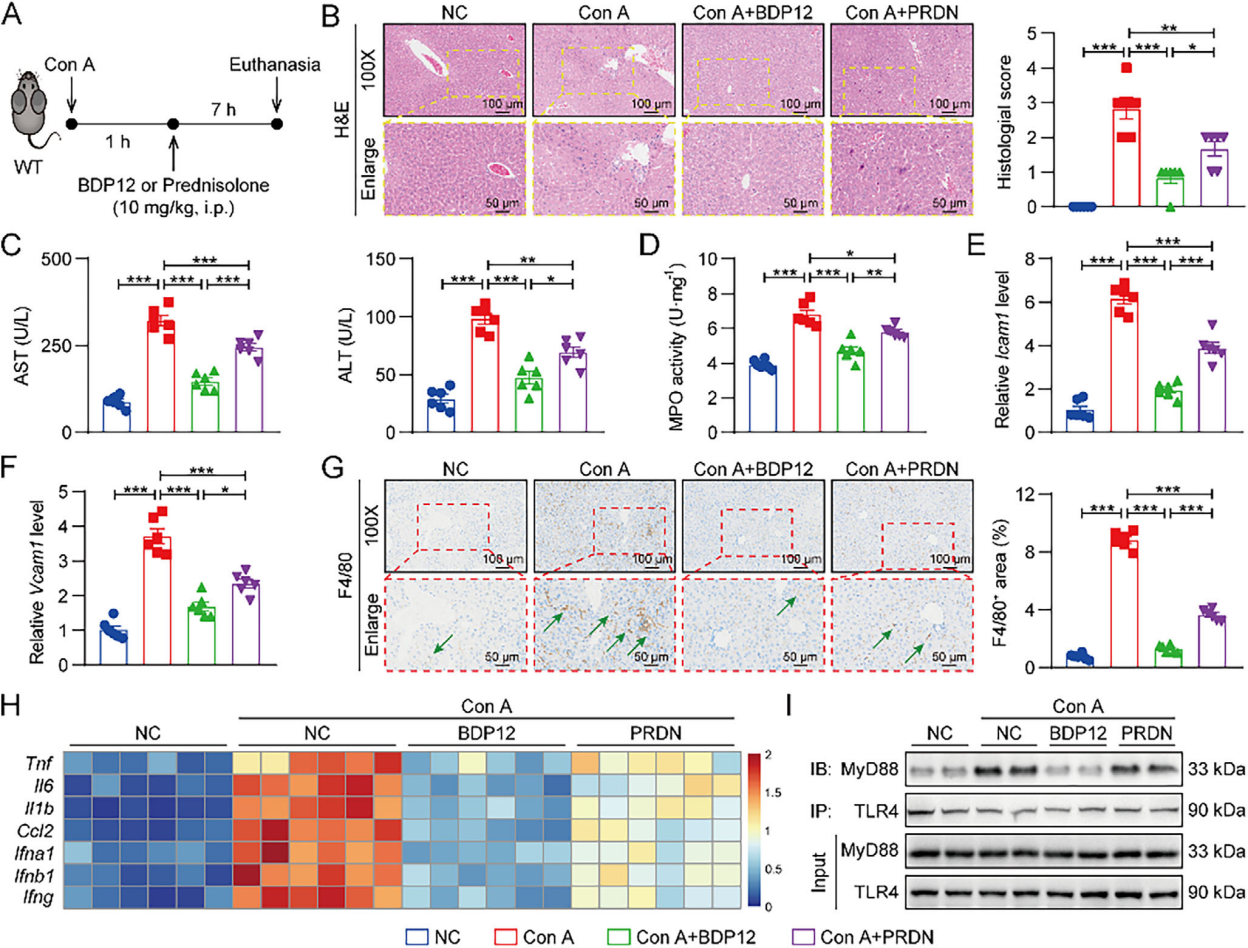
BDP12 exhibits advancing therapeutic efficacy in noninfectious ALI/ALF. (A) Schematic diagram of the experimental workflow. Mice were treated with BDP12 or isodose prednisolone via intraperitoneal injection following concanavalin A (Con A) challenge. (B) Representative H&E‐stained images of the liver section in mice. Quantification of hepatic histological scores is shown on the right. (C) Serum levels of AST and ALT in mice. (D) MPO activity levels in liver lysates of mice. mRNA levels of adhesion molecules *Icam1* (E) and *Vcam1* (F) in liver tissues. (G) Immunohistochemistry staining of F4/80 in liver tissues of mice. Quantification of F4/80‐positive areas is shown on the right. (H) Heatmap showing mRNA levels of pro‐inflammatory factors *Tnf*, *Il6*, *Il1b*, *Ccl2*, *Ifna1*, *Ifnb1*, and *Ifng* in liver tissues. (I) Co‐immunoprecipitation analysis of MyD88‐TLR4 complex formation in liver tissues. Data are shown in mean ± SEM; *n* = 6; *p < 0.05; **p < 0.01; ***p < 0.001.

In Figure 1C, we observed that BDNF is associated not only with infection‐related models of ALI/ALF but also with noninfectious models. To further investigate this, we evaluated the therapeutic efficacy of the BDNF‐mimetic peptide BDP12 in Con A‐induced autoimmune ALI/ALF, using prednisone (PRDN), a clinically recognized first‐line therapy for autoimmune hepatitis, as a positive control (Figure 9A). Histological analyses together with serum AST and ALT measurements demonstrated that BDP12 conferred greater protection than PRDN at equivalent doses (Figure 9B,C). Further assessment of the hepatic inflammatory microenvironment revealed that MPO activity (Figure 9D), mRNA levels of *Icam* and *Vcam* (Figure 9E,F), and IHC staining for the macrophage marker F4/80 (Figure 9G) were all markedly reduced in the BDP12‐treated group, whereas PRDN only partially suppressed these indicators. Consistently, multiple pro‐inflammatory cytokines followed the same trend (Figure 9H). Importantly, examination of TLR4 signaling, assessed by MyD88 recruitment, showed that BDP12 exerted a strong inhibitory effect on TLR4 activation, while PRDN had no detectable impact on this pathway (Figures 9I and S10I). These findings suggest that PRDN regulates inflammation through TLR4‐independent mechanisms, which may account for its weaker protective efficacy compared with BDP12. Collectively, results from both infection‐related models (including LPS/D‐Gal and sepsis‐induced model) and the noninfectious Con A‐induced model establish BDP12 as a potent anti‐inflammatory agent and a promising therapeutic candidate for ALI/ALF.

## 3 Discussion

In this study, we focused on acute hepatitis in ALI/ALF, identifying a novel immune regulatory checkpoint and developing a new peptide‐based anti‐inflammatory candidate. The three key findings of this work are as follows: (1) hepatocyte‐specific expression and secretion of BDNF can impede disease progression in ALI/ALF; (2) hepatocyte‐derived BDNF, in a paracrine manner, acts as a TLR4 antagonist on hepatic macrophage to suppress its pro‐inflammatory activity; and (3) the BDNF‐mimetic peptide BDP12 demonstrates improved specificity in targeting TLR4 and exhibits high druggability and low side effects. Collectively, this study not only reveals a previously unrecognized mechanism of immune regulation during acute hepatitis but also enables the successful translation of this mechanism into a promising BDNF‐based therapeutic candidate, BDP12, potentially addressing a critical unmet need in the treatment of ALI/ALF.

The liver, as a central organ for immune regulation and metabolic homeostasis, relies heavily on the intricate interplay between hepatocytes and their microenvironment, including non‐parenchymal cells and extracellular matrix components. Among these interactions, the crosstalk between hepatocytes and macrophages plays a pivotal role in various pathophysiological processes in the liver. For instance, hepatocytes can secrete bile acids such as chenodeoxycholic acid (CDCA) to activate the TGR5 receptor on macrophages, thereby inhibiting NLRP3 inflammasome activation [27, 28]; hepatocyte‐derived IL‐33 can stimulate macrophage‐induced ILC2 responses, leading to the production of reparative cytokines like IL‐13 [29, 30]; under stress conditions, hepatocyte‐released exosomes carrying miR‐122 modulate HO‐1 pathway in macrophages [31], while the necroptotic hepatocytes govern macrophage phagocytic activity by CD47‐SIRPα axis [32]. Other examples of hepatocyte–nonparenchymal cell interactions include apoptotic hepatocyte‐derived exosomes delivering miR‐128‐3p to activate hepatic stellate cells via the PPARγ suppression [33, 34], or hepatocyte‐derived VEGF‐A promoting loss of fenestrae in liver sinusoidal endothelial cells (LSECs) under hypoxia [35]. In this study, we identified a novel mode of intercellular interaction in which hepatocyte‐secreted BDNF directly binds to and antagonizes TLR4 on hepatic macrophages. Dysregulation of this crosstalk contributes to the exacerbation of acute hepatitis and related disease progression. Targeting this specific communication node may represent a new therapeutic paradigm that moves beyond broad anti‐inflammatory strategies directed solely at macrophages.

BNDF is well known for regulating cell proliferation, differentiation, and survival via its classical receptor TrkB, while the involvement of TrkB in inflammatory responses remains unclear. Although existing studies ascribed the anti‐inflammatory effects of BDNF to the BDNF‐TrkB axis and the imaginary TrkB‐mediated mechanism [36, 37, 38], these studies did not conclusively demonstrate that BDNF's anti‐inflammatory effects are dependent on TrkB signaling. In contrast, our experiments for the first time show that BDNF primarily exerts its anti‐inflammatory effects through direct antagonism of TLR4, rather than through TrkB activation. This finding not only expands the receptors of BDNF but also provides novel mechanistic insight into its role in immune regulation. In addition to TLR4, our IP‐MS analysis identified several other potential BDNF‐interacting receptors, including TLR2, CD9, and TFRC. However, TLR2 is predominantly involved in inflammatory responses triggered by Gram‐positive bacterial infection, whereas CD9 and TFRC have been mainly implicated in virus‐induced inflammatory processes [39, 40, 41]. Given that the present study focuses on acute hepatitis driven by multiple etiologies, these receptors are less likely to represent the dominant mediators of BDNF signaling in the inflammatory context investigated here and were therefore not pursued further. Nevertheless, the possibility that BDNF may engage TLR2, CD9, or TFRC to modulate inflammation in other disease settings remains an important and worthwhile subject for future investigation.

TLR4 is a well‐established pattern recognition receptor, originally recognized for its role in detecting LPS from bacteria [42]. Subsequently, TLR4 has been shown to recognize numerous endogenous DAMPs, such as advanced glycation end‐products (AGEs) [43, 44], saturated fatty acids [45], oxidized LDL [46], HMGB1 [47], HSP60/70 [48, 49], and S100A8/A9 [50]. Among these, HMGB1 is a prototypical DAMP that is released from necrotic or stressed hepatocytes [51]. During almost all forms of ALI/ALF, HMGB1 released from damaged hepatocytes may activate TLR4 on immune cells, amplifying inflammation and promoting hepatic cell death in a positive feedback loop. Consequently, TLR4 has emerged as a pivotal therapeutic target in ALI/ALF, and numerous studies show that its inhibition or genetic ablation significantly mitigates this disease [52, 53]. However, no small‐molecule TLR4 inhibitors have achieved clinical success to date. Our study identifies BDNF as the first endogenous TLR4‐antagonizing molecule, with further design and development of a peptide candidate, BDP12, offering unique translational potential due to its natural origin and intrinsic biocompatibility. In addition, BDNF effectively suppresses both LPS‐ and HMGB1‐induced TLR4 activation, highlighting its broader therapeutic potential across diverse ALI/ALF etiologies. This may also explain the observed reduction of BDNF expression and its inverse correlation with inflammation in various ALI/ALF models, including those induced by ethanol, APAP, or Con A.

Using liver single‐cell omics analysis, we identified a specific reduction in hepatocyte‐derived BDNF expression during ALI/ALF. Concurrently, we further demonstrated that hepatocyte *Bdnf* expression is under the control of the transcriptional repressor REST. REST is a well‐established negative regulator of the *Bdnf* gene, as extensively documented in previous studies [54]. In particular, *Chiara* et al. provided direct mechanistic evidence showing that REST binds to the RE1 motif within the *Bdnf* promoter and represses its transcription, as demonstrated by ChIP assays [55]. These findings are consistent with our observation that REST suppresses *Bdnf* expression in hepatocytes. Regarding the upstream signals leading to REST induction, we propose that LPS may upregulate REST as an intermediate step linking inflammatory signaling to *Bdnf* downregulation. REST transcription is regulated by the canonical Wnt/β‐catenin pathway [56], and LPS has been shown to robustly activate Wnt/β‐catenin signaling in multiple cell types [57]. Moreover, IL‐1β, a key cytokine induced by LPS, has been reported to rapidly increase REST transcription and translation [58]. Together, these findings suggest that LPS may enhance REST expression through both Wnt/β‐catenin activation and IL‐1β signaling, thereby contributing to the repression of *Bdnf* transcription in hepatocytes.

In addition to hepatocyte BDNF, a considerable pool of BDNF is distributed within the systemic circulation. Given that BDNF synthesized in the brain does not efficiently cross the blood‐brain barrier, peripheral BDNF is primarily derived from platelet release [59]. Although immune cells have been reported to produce BDNF [60, 61], recent single‐cell transcriptomic data show that BDNF mRNA is predominantly expressed in platelets among peripheral blood mononuclear cell (PBMC) populations (see details in https://www.proteinatlas.org/ENSG00000176697‐BDNF/single+cell/pbmc), reinforcing the role of platelets as the principal source of circulating BDNF. An important question, therefore, is whether circulating BDNF affects the course of ALI/ALF in our study. We consider this possible, but likely limited. Although extrahepatic circulating BDNF may partially compensate for local reductions, hepatic BDNF is tightly regulated in a spatiotemporal manner in vivo. When BDNF levels decline within the hepatic microenvironment, such systemic compensation is insufficient, and the local BDNF milieu is still predominantly determined by hepatic BDNF expression itself. This concept is consistent with observations in myocardial infarction models, where muscle‐specific BDNF deletion primarily reduced cardiac microenvironmental BDNF and aggravated myocardial injury [16]. By analogy, hepatocyte‐specific BDNF knockdown would be expected to cause a dominant decrease in hepatic microenvironmental BDNF, thereby impacting liver macrophage behavior and hepatic function during ALI/ALF.

In this study, we developed a BDNF‐mimetic dodecapeptide, BDP12, through simulating the BDNF‐TLR4 complex interface. We showed the TLR4‐antagonistic activity as well as the potent anti‐inflammatory properties of BDP12. Importantly, unlike full‐length BDNF, BDP12 lacks TrkB receptor‐activating activity, thereby enhancing anti‐inflammatory specificity and minimizing potential proliferative side effects. It is worth noting that in acute organ failure characterized by parenchymal necrosis, proliferative effects of BDNF might support tissue repair in damaged livers and provide therapeutic benefit. Therefore, full‐length BDNF may exert dual anti‐inflammatory and regenerative effects in the context of ALF. However, considering the TrkB‐mediated oncogenic potential [24], concerns regarding the clinical use of BDNF remain regarding the possible carcinogenesis promotion and metastasis promotion. Consequently, BDP12 may offer a safer therapeutic alternative in most cases of acute hepatitis and other inflammatory liver diseases. If necessary, it could be combined with hepatocyte‐protective agents or pro‐regenerative factors to achieve synergistic effects. Besides, preliminary pharmacokinetic and tissue distribution analyses showed that BDP12 is relatively stable in vivo and accumulates in the liver, as well as in major organs such as the kidney and lung (Figure S11A,B), supporting its potential relevance for liver‐related inflammatory diseases and its possible extension to inflammatory disorders in other organs.

Despite these findings, a limitation of the present study warrant acknowledgment. The modest clinical sample size may constrain the statistical power and generalizability of our observations concerning BDNF expression profiles in human ALI/ALF patients. Consequently, future multicenter studies involving larger and more diverse patient cohorts are essential to validate these expression patterns.

In summary, our findings dissect the REST‐BDNF‐TLR4 axis, a novel paradigm in the immunopathology of acute hepatitis, positioning hepatocyte‐derived BDNF as an endogenous immune checkpoint that restrains macrophage‐mediated inflammation via direct TLR4 antagonism. Moreover, we establish the non‐oncogenic peptide inhibitor BDP12 as a promising therapeutic strategy for ALI/ALF. This work paves the way for hepatocyte‐driven interventions in liver immunology and highlights the potential of peptide‐based therapeutics in treating acute inflammatory liver diseases.

## 4 Experimental Section

### 4.1 Antibodies and General Reagents

Primary antibodies used in this study included anti‐BDNF (ab108319) and anti‐MD2 (ab24182) from Abcam (Cambridge, UK); anti‐GAPDH (30201ES60) from Yeasen Biotechnology (Shanghai, China); and anti‐TLR4 (sc‐293072), anti‐F4/80 (sc‐52664), anti‐Biotin (sc‐101339), and mouse IgG isotype control (sc‐66931) from Santa Cruz Biotechnology (Dallas, TX, USA). Antibodies from Cell Signaling Technology (Danvers, MA, USA) included anti‐phospho‐TAK1 (Thr187; #9339), total TAK1 (#5206), anti‐phospho‐TBK1/NAK (Ser172; #5483), total TBK1/NAK (#3013), anti‐phospho‐IRF3 (Ser396; #37829), total IRF3 (#11904), and anti‐IκB‐α (#9242). Additional antibodies from Proteintech Group (Wuhan, China) included anti‐PCNA (10205‐2‐AP), anti‐REST (22242‐1‐AP), anti‐Bax (50599‐2‐Ig), anti‐Bcl‐2 (68103‐1‐Ig), anti‐CD14 (17000‐1‐AP), and anti‐TrkB (13129‐1‐AP). All antibodies were used at dilutions recommended by the manufacturers for applications including Western blotting, immunohistochemistry, immunofluorescence, immunoprecipitation, and proximity ligation assay (PLA).

Reagents used in this study included LPS (L2880) and Con A (C2010) from Sigma‐Aldrich (St. Louis, MO, USA); recombinant BDNF protein (HY‐P7116A), recombinant HMGB1 protein (HY‐P70274), ANA‐12 (HY‐12497), d‐galactosamine hydrochloride (HY‐42682), prednisolone (HY‐17463), and biotin (HY‐B0511) from MedChemExpress (MCE, Monmouth Junction, NJ, USA); and recombinant human TLR4 leucine‐rich repeat (LRR) domain protein (10146‐H08B) from Sino Biological (Beijing, China). BDP12 and its N‐terminal biotinylated derivative were custom‐synthesized by GenScript (Nanjing, China).

### 4.2 Cell

Peritoneal macrophages were isolated using a thioglycolate‐elicited method. Briefly, mice were intraperitoneally injected with 2 mL of sterile 6% thioglycolate broth (containing 0.3% beef extract, 1% tryptone, and 0.5% NaCl) three days prior to harvest. Peritoneal lavage was performed using 8 mL ice‐cold RPMI‐1640 medium, and the collected cells were centrifuged at 300 ×g for 5 min. After resuspension in complete RPMI‐1640 medium (supplemented with 10% FBS and 1% penicillin‐streptomycin), cells were plated at 7 × 10^5^ cells per 35 mm dish. Non‐adherent cells were removed after 4‐h incubation, and adherent macrophages were cultured overnight before experimental use.

Primary liver cells were isolated via a two‐step collagenase perfusion method. The liver was first perfused through the portal vein with calcium‐free HBSS (37 °C, 5 mL/min, 5–10 min), followed by digestion with 0.08% type IV collagenase in calcium‐containing buffer. The digested tissue was gently dissociated, sequentially filtered through 70 and 40 µm cell strainers, and centrifuged at 50×*g* for 3 min to separate hepatocytes from non‐parenchymal cells (NPCs). NPCs were further purified using a discontinuous Percoll density gradient (800×*g*, 20 min). Cell viability was consistently >85% by trypan blue exclusion.

HepG2 (Procell: CL‐0103) and HEK‐293T (Procell: CL‐0005) cell lines were cultured in MEMα and DMEM, respectively, both supplemented with 10% FBS and 1% penicillin‐streptomycin. The NRK‐52E cell line stably expressing an NF‐κB‐EGFP reporter was maintained in DMEM complete medium.

### 4.3 Human Liver Samples

Paraffin‐embedded human liver specimens were obtained from patients with acute hepatitis (*n* = 8) and non‐hepatitis controls (*n* = 2) at the Affiliated Xiangshan Hospital of Wenzhou Medical University (Approval No. 2024‐SL‐005). None of the patients with hepatitis were receiving anti‐inflammatory therapy or medications known to affect liver function at the time of biopsy. Histopathological evaluation of parenchymal necrosis was performed in a blinded manner by trained pathologists using Suzuki's scoring criteria [62]. Necrosis was graded on a scale from 0 to 4, where 0 indicates no necrosis, 1 indicates single‐cell necrosis, 2 indicates necrosis involving <30% of the parenchyma, 3 indicates necrosis involving <60% of the parenchyma, and 4 indicates necrosis involving >60% of the parenchyma. Scores 1–2 were classified as mild hepatitis, whereas scores 3–4 were classified as severe hepatitis.

### 4.4 Mice

All animal experiments were conducted in accordance with protocols approved by the Animal Policy and Welfare Committee of Wenzhou Medical University (Approval No. wydw2020‐0012). Six‐week‐old male C57BL/6J wild‐type mice and *Tlr4* ^−/−^ mice (T051714 strain) were obtained from Gempharmatech (Jiangsu, China) and maintained under specific pathogen‐free conditions at 22 ± 2 °C with 50%–60% humidity, a 12‐h light/dark cycle, and free access to standard rodent chow and water. Following a two‐week acclimation period, mice were randomly assigned to experimental groups using a blinded randomization procedure, with six animals housed per microisolator cage. To ensure unbiased group allocation, each mouse received both a temporary weight‐based identifier and a permanent cage‐specific numerical designation prior to study initiation.

### 4.5 Adeno‐Associated Virus Construction and Infection

The coding sequence of mouse mature BDNF fused to an N‐terminal albumin signal peptide and a C‐terminal 6×His tag (SP‐matureBDNF‐6His) was obtained from Genechem (Shanghai, China) and cloned into the AAV plasmid KV289 containing a hepatocyte‐specific TBG promoter (TBG‐MCS‐FT2A‐EGFP‐WPRE‐BGH polyA) using AgeI restriction digestion followed by In‐Fusion recombination. The recombinant plasmid was verified by Sanger DNA sequencing to confirm correct insertion and reading frame integrity. Recombinant adeno‐associated virus serotype 8 (AAV8) was produced by transient triple transfection of HEK293T cells using Lipofectamine 2000 with the AAV vector plasmid, pHelper, and pRepCap plasmids. Seventy‐two hours after transfection, viral particles were harvested, purified by iodixanol gradient ultracentrifugation, and concentrated. Viral titers were determined by quantitative PCR. The final preparations were formulated in 0.001% Pluronic F‐68 solution. Mice received intravenous injections of AAV8 vectors at a dose of 2 × 10^11^ viral genomes per animal. Following the injection, mice were housed for two weeks to allow sufficient transgene expression. Peripheral tissues were subsequently collected to evaluate the tissue specificity of BDNF overexpression.

### 4.6 Bone Marrow Transplantation

Seven‐week‐old male recipient mice were housed in sterile, filter‐top cages and given autoclaved water supplemented with 1000 U/L polymyxin B and 1.1 mg/L neomycin for 7 days prior to total body irradiation (6 Gy). Twelve hours after irradiation, bone marrow cells were harvested from donor mouse femurs and tibias by flushing with a 24‐gauge needle. Red blood cells were lysed, and the remaining cells were passed through a 100‐µm nylon mesh to obtain a single‐cell suspension. Each recipient mouse received 2 × 10^6^ bone marrow cells via tail vein injection. Mice were then allowed to recover for two weeks before initiating experimental procedures.

### 4.7 LPS/DGal‐Induced ALI/ALF Model

Mice were intraperitoneally administered LPS (10 µg/kg) and DGal (500 mg/kg), both dissolved in 0.9% saline. According to experimental protocols, recombinant BDNF (300 ng), the BDNF‐memetic peptide BDP12 (4 or 10 mg/kg), or vehicle control was administered intravenously or intraperitoneally 30 min post‐LPS/DGal injection. Six hours later, animals were euthanized, and blood and liver tissues were harvested for further analysis. Mice pre‐infected with AAV8 underwent the same protocol to induce ALI/ALF.

For survival assessment, an additional cohort received the same LPS/DGal treatment and was monitored over a 48‐h period.

### 4.8 Sepsis‐Induced ALI/ALF Model

Polymicrobial sepsis was induced in mice via cecal ligation and puncture (CLP). Mice were anesthetized with 2% sodium pentobarbital (80 mg/kg, i.p.), and the abdominal area was shaved and disinfected with ethanol. A midline laparotomy was performed to expose the cecum, which was ligated at approximately 40% of its length from the tip using sterile sutures. A single through‐and‐through puncture was made in the ligated cecum using a 21‐gauge needle, allowing minimal fecal content to extrude. The cecum was repositioned into the peritoneal cavity, and the abdominal wall was closed in two layers with absorbable sutures. Postoperative care included fluid resuscitation and thermal support.

At 1 h postsurgery, mice received an intraperitoneal injection of BDP12 (4 or 10 mg/kg) or vehicle, as per the experimental protocol. Mice were euthanized 12 h after CLP for the collection of blood and liver tissues for downstream analyses.

For survival studies, a separate cohort underwent the same CLP procedure and was observed for 48 h.

### 4.9 Autoimmune‐Induced ALI/ALF Model

Noninfectious, autoimmune‐related ALI/ALF was induced in mice by intraperitoneal injection of Con A (20 mg/kg dissolved in PBS). One hour after Con A administration, mice received an intraperitoneal injection of either BDP12 (10 mg/kg) or prednisolone (PRDN, 10 mg/kg, dissolved in 10% DMSO in corn oil) as a positive control. Mice were euthanized 8 h after Con A treatment, and blood and liver tissues were collected for subsequent analyses.

### 4.10 Pharmacokinetic and Tissue Distribution Analysis of BDP12

For pharmacokinetic analysis, mice were intraperitoneally injected with BDP12 (10 mg/ kg). Blood samples were collected at 0, 15, 30, 60, 180, and 360 min after administration. Plasma was obtained by centrifugation at 12,000×*g* for 10 min at 4°C. An aliquot of plasma was mixed with trifluoroacetic acid containing loratadine (100 ng/mL) as an internal standard, vortexed for 3 min, and centrifuged again at 12,000×*g* for 10 min. The resulting supernatant was transferred to an autosampler vial and analyzed using UPLC‐MS/MS to determine BDP12 concentrations.

For tissue distribution analysis, mice were intraperitoneally injected with BDP12 (10 mg/kg) once daily for three consecutive days. Two hours after the third injection, mice were anesthetized, and major organs including heart, liver, spleen, lung, and kidney were collected, weighed, and homogenized with 0.9% saline (1:2, w/v). The homogenates were centrifuged at 12,000×*g* for 10 min at 4 °C. The supernatant was mixed with trifluoroacetic acid containing loratadine (100 ng/mL) as an internal standard, vortexed for 3 min, and centrifuged again at 12,000×*g* for 10 min. The final supernatant was transferred to autosampler vials, and BDP12 concentrations were quantified by UPLC‐MS/MS.

### 4.11 Histological Analysis

Tissue samples were fixed in 4% paraformaldehyde, paraffin‐embedded, and sectioned at 5 µm thickness. For histological evaluation, sections were stained with hematoxylin and eosin (H&E). Liver pathology of parenchymal necrosis was blindly evaluated by trained pathologists based on Suzuki's score system [62].

Immunohistochemistry was conducted on deparaffinized, rehydrated liver sections. Antigen retrieval was achieved by heating in sodium citrate buffer (pH 6.0), followed by blocking with 1% BSA in PBS for 30 min. Sections were then incubated overnight at 4 °C with primary antibodies (1:100 dilution). After washing, HRP‐conjugated secondary antibodies were applied for 1 h at 37 °C. Visualization was performed using DAB substrate, and nuclei were counterstained with hematoxylin. Images were acquired using a brightfield microscope, and immunoreactivity was quantified using ImageJ (NIH, Bethesda, MD, USA).

Immunofluorescence staining was performed on paraffin‐embedded liver sections to evaluate the expression and localization of target proteins. After deparaffinization and rehydration, antigen retrieval was carried out in sodium citrate buffer (pH 6.0) at 95 °C for 15 min. Sections were then cooled to room temperature and blocked with 1% BSA in PBS for 30 min. Subsequently, sections were incubated overnight at 4 °C with primary antibodies (1:100 dilution). After washing with PBS, appropriate fluorophore‐conjugated secondary antibodies (1:200 dilution) were applied for 1 h at room temperature in the dark. Nuclei were counterstained with DAPI (1:1000) for 5 min. Finally, sections were mounted with anti‐fade mounting medium, and fluorescence images were captured using a fluorescence microscope.

TUNEL staining was used to identify apoptotic cells in liver tissue sections. After deparaffinization and rehydration, antigen retrieval was conducted at 95 °C for 15 min in sodium citrate buffer (pH 6.0). Sections were then permeabilized with 0.1% Triton X‐100 in PBS for 10 min at room temperature. TUNEL reaction mixture containing TdT enzyme and fluorescein‐labeled dUTP was applied for 1 h at 37 °C in a dark, humidified chamber. Nuclei were counterstained with DAPI (1:1000) for 5 min. After mounting with anti‐fade medium, fluorescent signals (TUNEL: green or red; DAPI: blue) were visualized using a fluorescence microscope. The percentage of apoptotic (TUNEL‐positive) cells was quantified using ImageJ.

### 4.12 Myeloperoxidase Activity Assay

Neutrophil infiltration in liver tissues was assessed by measuring myeloperoxidase (MPO) activity using a commercial MPO Activity Assay Kit (Thermo Fisher). Briefly, liver samples were homogenized in 1 mL of 50 mm potassium phosphate buffer (pH 6.0) containing 0.5% hexadecyltrimethylammonium bromide. Homogenates were centrifuged at 15,000×*g* for 20 min at 4 °C. A 10 µL aliquot of the supernatant was incubated in a reaction mixture consisting of phosphate buffer (pH 6.0), 0.17 mg/mL 3,3′‐dimethoxybenzidine, and 0.0005% hydrogen peroxide. MPO activity was determined by measuring absorbance at 460 nm. Protein concentrations were quantified using the Pierce BCA Protein Assay Kit (Thermo Fisher), and MPO activity was normalized to total protein content and expressed as units per gram of tissue (U/g).

### 4.13 Western Blot

Total proteins were extracted using RIPA lysis buffer, and concentrations were determined using the Pierce BCA Protein Assay Kit. Equal amounts of protein were subjected to SDS‐PAGE, followed by transfer to PVDF membranes. After blocking with 5% skim milk at room temperature for 1 h, membranes were incubated with primary antibodies (diluted 1:500–1:1000) overnight at 4 °C. The next day, membranes were treated with HRP‐conjugated secondary antibodies (diluted 1:2000) for 1 h at room temperature. Protein bands were visualized using enhanced chemiluminescence (ECL) reagents (Bio‐Rad, Hercules, CA, USA).

For co‐immunoprecipitation assays, cell lysates were incubated overnight at 4 °C with target‐specific antibodies, followed by incubation with protein A/G agarose beads. The immunocomplexes were collected, washed, and analyzed by Western blot to assess protein–protein interactions. Band intensities were quantified using ImageJ software, and results were normalized to loading controls or total protein input.

### 4.14 Single‐Cell RNA Sequencing

Primary hepatocytes and nonparenchymal cells were freshly isolated from mouse liver using a two‐step collagenase perfusion method and subsequently mixed in equal proportions to create a heterogeneous cell suspension. Cell viability was assessed by trypan blue exclusion, and only samples with viability above 85% were used for library preparation.

Single‐cell libraries were constructed using the Chromium Single Cell 3′ Gene Expression platform (10X Genomics, Pleasanton, CA, USA) according to the manufacturer's instructions. Briefly, cells were loaded onto the Chromium Controller to generate Gel Beads‐in‐Emulsion (GEMs), allowing barcoding of individual cells. Reverse transcription, cDNA amplification, and library construction were performed following the 10X Genomics standard protocol. Libraries were quantified and quality‐controlled using an Agilent Bioanalyzer and Qubit fluorometer before sequencing.

Sequencing was performed on an Illumina platform with paired‐end reads, targeting a depth of approximately 50,000 reads per cell. Raw sequencing data were processed using the Cell Ranger pipeline (10X Genomics) for demultiplexing, alignment to the mouse reference genome (mm10), and gene quantification. For downstream analysis, the processed count matrix was uploaded to the Omicstudio cloud platform (https://www.omicstudio.cn/) for data visualization and advanced bioinformatics analysis.

### 4.15 ELISA Assay

Cytokine and BDNF concentrations were measured in mouse serum, various tissues, and cell culture supernatants. Levels of mouse TNF‐α, IL‐6, IFN‐α, and BDNF were quantified using commercially available ELISA kits, following the manufacturers’ protocols. In addition, custom ELISAs were developed to detect protein complexes involving BDNF‐TLR4 and MD2‐TLR4. For this, 96‐well plates were coated with anti‐TLR4 antibodies and blocked with bovine serum albumin (BSA). Cell lysates from different treatment groups were added to the wells and incubated, followed by the application of either anti‐MD2 or anti‐BDNF antibodies. After thorough washing, HRP‐conjugated secondary antibodies specific to MD2 or BDNF were applied. The enzymatic reaction was developed using tetramethylbenzidine (TMB) substrate, and absorbance was read at 450 nm using a SpectraMax M5 Multi‐Mode Microplate Reader (Molecular Devices, San Jose, CA, USA).

### 4.16 Real‐Time qPCR

Total RNA was isolated from tissue samples using Trizol reagent following the manufacturer's protocol. The extracted RNA was then reverse‐transcribed into complementary DNA (cDNA) using the PrimeScript RT reagent kit (Takara, RR047A). Quantitative real‐time PCR (qPCR) was performed on a Bio‐Rad CFX96 Touch Real‐Time PCR Detection System using TB Green Premix Ex Taq II (Takara, RR820A) for fluorescence detection. Gene expression levels were quantified relative to the housekeeping gene *Actb* (β‐actin) using the comparative Ct method. Primer sequences for target genes are detailed in Table S1.

### 4.17 Cell Transfections

To knock down *Rest* or *Bdnf* expression in hepatocytes, specific siRNAs were synthesized by Genechem and transfected into primary hepatocytes using Lipofectamine RNAiMAX (Thermo Fisher, 13778150) following the manufacturer's protocol. For gene overexpression studies involving REST, BDNF,  BDNF mutant, TLR4, or MD2 in hepatocytes or HEK‐293T cells, the corresponding wild‐type coding sequences cloned into pcDNA3.1 vectors (synthesized by GeneScript) were introduced using Lipofectamine 2000.

### 4.18 Conditioned Medium‐Based Indirect Co‐culture Assay

To investigate the paracrine effects of hepatocyte‐derived secreted BDNF on macrophage function, an indirect co‐culture system using CM was established. Primary mouse hepatocytes were treated with or without LPS and subjected to *Rest* silencing, or to *Bdnf* silencing or overexpression. After treatment or transfection, the culture medium was replaced with fresh medium, and the cells were further incubated for 24 h. The supernatants were then collected and centrifuged at 1,000×*g* for 10 min at 4 °C to remove cellular debris. The resulting hepatocyte‐conditioned medium was mixed at a 1:1 ratio with fresh complete culture medium to ensure sufficient nutrients and to avoid potential confounding by nutrient depletion. This CM mixture was subsequently applied to primary mouse peritoneal macrophages or NF‐κB‐EGFP reporter cells seeded the previous day. Macrophages were incubated with conditioned medium for 30 min before further analyses. Control groups consisted of cells treated with CM derived from hepatocytes transfected with control siRNA or empty vectors.

### 4.19 Transcription Factor Prediction

To identify high‐confidence TFs potentially regulating BDNF transcription, the TF Target Finder webserver (https://jingle.shinyapps.io/TF_Target_Finder/) was used and selected the “Target > TF” analysis module. The gene symbol “BDNF” was input into the search field, and transcription factor predictions were obtained from eight independent databases, including hTFtarget, ChIP Atlas, GTRD, TRRUST, ChEA, ENCODE, PWMEnrich JASPAR, and FIMO JASPAR. To ensure the robustness of candidate TFs, Venn diagram analysis was performed to identify overlapping transcription factors across all eight datasets. Only transcription factors that appeared in all datasets were considered high‐confidence candidates.

### 4.20 Immunoprecipitation‐Coupled Mass Spectrometry

Primary macrophages were treated with recombinant BDNF for 1 h. After incubation, cells were lysed in NP‐40‐based lysis buffer supplemented with protease and phosphatase inhibitors. Lysates were cleared by centrifugation and incubated overnight at 4 °C with anti‐BDNF antibody (2 µg per mg total protein) preconjugated to magnetic Protein A/G beads (Thermo Fisher). Beads were washed thoroughly to remove nonspecific binding proteins and eluted by heating in a denaturing buffer. The eluates were subjected to in‐solution digestion with trypsin following standard reduction and alkylation steps (10 mm DTT, 55 mm iodoacetamide). Peptides were desalted using C18 columns and analyzed by DIA‐based LC‐MS/MS on a high‐resolution mass spectrometer. Raw data were processed and searched against the Mus musculus UniProt database with a false discovery rate (FDR) <1% at both peptide and protein levels.

### 4.21 Surface Plasmon Resonance Assay

The binding affinity of BDNF and BDP12 to rTLR4 (extracellular portion) was determined using a Biacore T200 instrument (Cytiva; Marlborough, MA, USA) with a CM5 sensor chip (Cytiva). Briefly, rTLR4 was loaded onto the sensors using an Amine Coupling Kit (Cytiva). BDP12 samples (0, 9.77, 19.53, 39.06, 78.13, 156.25, and 312.5 nm) were prepared in running buffer (PBS containing 0.5% P20 and 5% DMSO), whereas BDNF samples (0, 7.8, 15.6, 31.3, 62.5, and 125 nm) were diluted in PBS containing 0.5% P20. After loading the sensor and sample plates, BDNF or BDP12 solutions were injected over the reference and ligand‐coupled flow cells at a flow rate of 30 µL/min. For BDNF, the association and dissociation phases were 200 s each at 25 °C; for BDP12, a 200‐s association phase was followed by a 100‐s dissociation phase at 25 °C. The final graphs were obtained by subtracting the blank sensorgrams and blank samples from the duplex. The data were analyzed using Biacore T200 software EV (Cytiva).

### 4.22 BDNF‐BDP12 Competitive Binding Assay

The effect of BDP12 on the competition of BDNF binding to TLR4 was evaluated using an ELISA‐based assay. Briefly, 96‐well plates were coated overnight at 4 °C with anti‐TLR4 antibody (1:500 dilution) and subsequently incubated with recombinant TLR4 protein to allow capture. After washing, recombinant BDNF (20 µg/mL) was premixed with graded concentrations of BDP12, and the mixtures were added to the wells, followed by incubation for 2 h at room temperature. After washing, bound BDNF was detected using an anti‐BDNF antibody and an HRP‐conjugated secondary antibody. Color development was achieved with TMB substrate, and the reaction was terminated using the stop solution. The relative amount of TLR4‐bound BDNF was quantified by measuring absorbance at 450 nm.

### 4.23 Biotinylated Pull‐Down Assay

To examine the binding of BDP12 to TLR4 or TrkB, a Pierce Biotinylated Protein Interaction Pull‐Down Kit (Thermo Fisher Scientific; Cat# 21115) was used according to the manufacturer's protocol. Briefly, 10 µL of 10 mm biotinylated BDP12 (Bio‐BDP12) was incubated with 50 µL streptavidin agarose beads at 4 °C for 30 min with gentle rotation. Negative controls included blank, biotin alone, and non‐biotinylated BDP12 to rule out nonspecific binding. Liver tissue lysates were prepared and added to the bead complexes, followed by incubation at 4 °C for 24 h with gentle rocking. Beads were then washed three times, and bound proteins were eluted with the elution buffer. Eluates were boiled in 5× SDS loading buffer and subjected to SDS‐PAGE using a 10% polyacrylamide gel. Western blotting was performed to detect TLR4 and TrkB. Total tissue lysates served as input controls.

### 4.24 Molecular Docking for the BDNF‐TrkB Complex

The full‐length sequences of human mature BDNF and its receptor TrkB were retrieved from the UniProt database (UniProt IDs: [BDNF: P23560], [TrkB: Q16620]). The predicted complex structure was generated using AlphaFold3 (https://alphafoldserver.com/). The modeling was configured to simulate a 2:2 stoichiometry (two BDNF monomers with two TrkB monomers) to form a tetrameric complex. The seed was set to “auto”, and default parameters were used for model ranking. The top‐ranked model was visualized and analyzed using PyMOL (Schrödinger, LLC). To evaluate binding affinities and key residues contributing to complex stability, VD‐MM/GBSA (Molecular Mechanics/Generalized Born Surface Area) free energy calculations were performed using the HawkDock web server (http://cadd.zju.edu.cn/hawkdock/). The results were ranked based on binding free energy contributions at the residue level. The top 50 BDNF residues with the lowest (most favorable) free energy values were recorded as potential interface hotspots.

### 4.25 Molecular Dynamics Simulation for the BDNF‐TLR4 Complex

The crystal structures of human TLR4 (extracellular domain) and BDNF were retrieved from the Protein Data Bank (PDB IDs: 3FXI and 1B8M, respectively). To model the potential interaction between BDNF and TLR4, protein–protein docking was performed using the ZDOCK 3.0.2f server, which employs a fast Fourier transform (FFT) correlation approach for rigid‐body docking. The top 500 poses generated by ZDOCK were subsequently re‐scored and refined using the IRaPPA re‐ranking protocol to account for desolvation and electrostatic effects, from which the top‐ranking complex model was selected for further analysis.

The top‐scoring BDNF‐TLR4 complex model from molecular docking was subsequently subjected to molecular dynamics (MD) simulations using GROMACS 2022.4 with the CHARMM36 all‐atom force field. The complex was solvated in a cubic water box with TIP3P explicit water molecules, extending at least 1.2 nm from the protein surface. Sodium and chloride ions were added to neutralize the system and to achieve a physiological concentration of 0.15 m. Initial energy minimization was performed using the steepest descent algorithm (50,000 steps) to eliminate steric clashes and achieve a stable initial configuration. Following minimization, the system was equilibrated in two phases: first under an *NVT* ensemble for 100 ps at 300 K using the v‐rescale thermostat, and then under an *NPT* ensemble for 100 ps at 1 bar using the Parrinello–Rahman barostat. Production MD was then carried out for 100 ns with a 2‐fs integration time step, and coordinates were saved every 10 ps for subsequent analysis. Post‐simulation analyses included evaluation of the system stability via root mean square deviation (RMSD) of the protein backbone and calculation of root mean square fluctuation (RMSF) per residue to assess local conformational flexibility. Secondary structure stability and intermolecular hydrogen bonds between BDNF and TLR4 were also monitored throughout the trajectory. All trajectory analyses were performed using built‐in GROMACS utilities, and the resulting data were visualized and plotted using GraphPad Prism 8.0.

### 4.26 Proximity Ligation Assay

To assess the direct interaction between TLR4 and either BDNF or BDP12 in macrophages, a proximity ligation assay was performed using the Duolink In Situ Red Starter Kit (Sigma‐Aldrich), following the manufacturer's protocol. Briefly, primary macrophages were seeded onto coverslips and treated with either recombinant BDNF protein or biotin‐labeled BDP12 for the indicated durations. Cells were then fixed in 4% paraformaldehyde. For the detection of protein–protein interactions, cells were incubated with combinations of either anti‐BDNF or anti‐biotin antibodies, along with an anti‐TLR4 antibody, at 4 °C overnight. Species‐specific PLA probes (PLUS and MINUS) were applied, and ligation and amplification steps were carried out according to the kit instructions. PLA signals, visualized as red fluorescent puncta, were detected using a confocal microscope (e.g., Zeiss LSM series). Negative controls included cells treated with isotype‐matched IgG antibodies or PBS, to assess nonspecific background signal and validate antibody specificity.

### 4.27 Proliferation and Viability Assay

The impact of LPS (0, 0.5, 1, 2, 5, 10 µg/mL), recombinant BDNF (rBDNF; 0, 50, 100, 200, and 400 ng/mL), and BDP12 (0, 1, 2, 4, and 8 µm) on cell proliferation and viability was evaluated using the Cell Counting Kit‐8 (CCK‐8; Thermo Fisher) following the manufacturer's protocol. Absorbance was measured at 450 nm to quantify metabolic activity as an indicator of cell viability. For in vivo analysis of proliferative responses, mice were administered rBDNF (300 ng) or BDP12 (10 mg/kg) once daily for three consecutive days. At the end of treatment, animals were euthanized, and liver tissues were collected for immunohistochemical staining of PCNA to assess hepatocyte proliferation.

### 4.28 Publicly Available Bulk Transcriptomic Data

Publicly available RNA sequencing datasets related to various models of acute hepatitis, including LPS, LPS/DGal, ethanol (EtOH), LPS combined with EtOH, APAP, and Con A, were retrieved from the Gene Expression Omnibus (GEO) database. The following accession numbers were used: GSE166488, GSE160880, GSE241513, GSE97234, GSE220520, GSE218879, and GSE17184. Differentially expressed genes (DEGs) were either extracted from the supplementary materials provided by the original studies or identified using the GEO2R web tool, where applicable. For datasets that offered only raw count or FPKM expression matrices, gene expression analysis and DEG identification were performed using the ExpressAnalyst online platform (https://www.expressanalyst.ca/), following standard normalization and statistical testing procedures. The correlation between *Bdnf* and *Ccl2* gene expression was analyzed using GraphPad Prism 8.0. A random‐effects meta‐analysis of correlation coefficients across different datasets was conducted using the Meta‐Mar webtool (https://www.meta‐mar.com).

### 4.29 Statistical Analysis

All data are reported as Mean ± SEM. Statistical analysis was performed with GraphPad Prism 8.0 software (San Diego, CA, USA). Sample sizes were not predetermined using statistical methods but were consistent with those commonly used in the field. Each dot point represents an individual animal or independent experiment. The Shapiro–Wilk test was applied to assess normality. For comparisons between two groups, unpaired Student's *t*‐tests were used, or the Mann–Whitney *U*‐test if applicable. For multiple group comparisons, a two‐sided one‐way ANOVA followed by Tukey's post hoc test was conducted. Statistical tests were selected based on data distribution, with significance defined as *p < 0.05, **p < 0.01, and ***p < 0.001. All data collection and analysis were conducted in a blinded manner, and no animals were excluded.

## Author Contributions

Conceptualization: G.L., W.L., X.K.L.; Methodology: W.W.Z., Y.Q.C., Y.Q.Z., Y.H.Z., L.J.H., L.M.J., Q.H.Z., P.C., M.S.L., J.X.Y., Y.Q.X.; Investigation: W.W.Z., J.Y.H., X.H., W.L., G.L.; Visualization: W.W.Z., Y.Q.C., Y.Q.Z., Y.H.Z.; Funding acquisition: W.W.Z., Y.H.Z.; Project administration: G.L., W.L., W.W.Z.; Supervision: G.L., X.K.L.; Writing – original draft: W.W.Z.; Writing – review & editing: G.L., W.L., X.H.

## Funding

This study was supported National Natural Science Foundation of China (82404637), Postdoctoral Fellowship Program of CPSF (GZC20231957), China Postdoctoral Science Foundation (2024M752443), Medical and Health Science and Technology Project of Zhejiang Province (2025KY322), and Science and Technology Plan Project of Wenzhou Municipality (Y20240374).

## Ethical Approval

Paraffin‐embedded human liver specimens were obtained from patients with acute hepatitis (*n* = 8) and non‐hepatitis controls (*n* = 2) at the Affiliated Xiangshan Hospital of Wenzhou Medical University (Approval No. 2024‐SL‐005). All animal experiments were conducted in accordance with protocols approved by the Animal Policy and Welfare Committee of Wenzhou Medical University (Approval No. wydw2020‐0012).

## Conflicts of Interest

The authors declare no competing interests.

## Supporting information




**Supporting File 1**: advs74969‐sup‐0001‐SuppMat.docx.

**Supporting File 2**: advs74969‐sup‐0002‐Data.pdf.

## Data Availability

The data that support the findings of this study are available on request from the corresponding author. The data are not publicly available due to privacy or ethical restrictions. The raw data generated from this study including scRNA‐Seq have been deposited in GEO (accession number: GSE300744), and the IP‐MS has been deposited in iProX (project ID: IPX0014894000).
