# Supplementary material for: Hepatocyte BDNF Acts as a Novel Immune Checkpoint to Restrain TLR4‐Mediated Acute Hepatitis
Source: Adv Sci (Weinh). 2026 Mar 25;13(32):e21164. doi: 10.1002/advs.202521164 (PMC13252628; doi:10.1002/advs.202521164)
Supplement: Supplementary file 1 — Supporting File 1: advs74969‐sup‐0001‐SuppMat.docx. [file ADVS-13-e21164-s001.docx]

*Supplementary Information*

**Supplementary Figures**


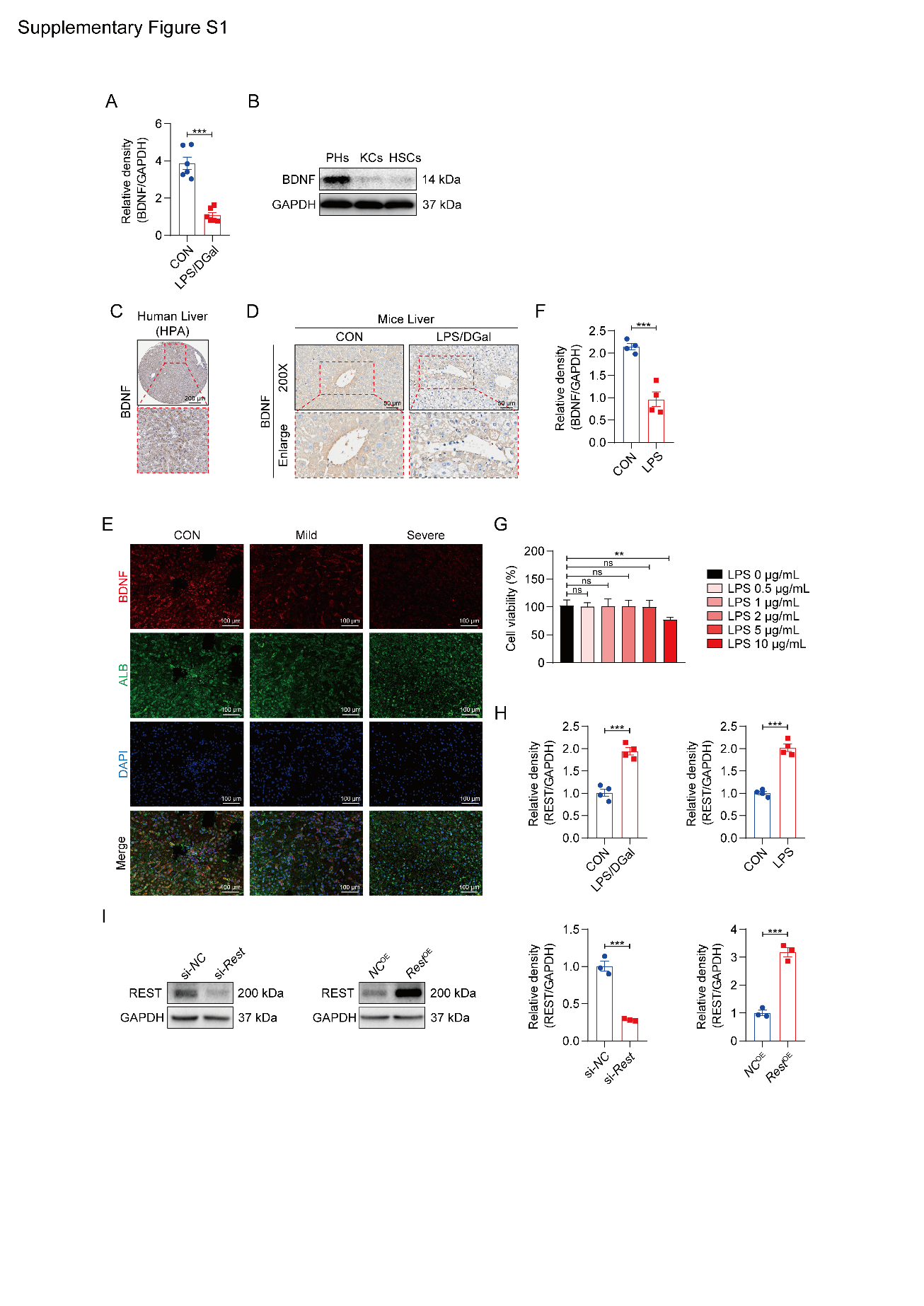


**Fig. S1. Histological staining of BDNF in liver section and effects of *Rest* silencing and overexpression in hepatocytes.** (A) Densitometric quantification corresponding to Figure 1E. (B) Protein levels of BDNF in primary hepatocytes (PHs), primary Kupffer cells (KCs), and primary hepatic stellate cells (HSCs). (C) Publicly available immunohistochemistry staining of BDNF in human liver tissue (Image ID: CAB009564), obtained from The Human Protein Atlas (HPA; https://www.proteinatlas.org/ENSG00000176697-BDNF/tissue/liver#img). (D) Immunohistochemistry staining of BDNF in liver tissues from control and LPS/DGal-treated mice. (E) Immunofluorescence staining of liver sections from healthy controls and patients with hepatitis. BDNF is shown in red, the hepatocyte marker albumin (ALB) in green, and nuclei are counterstained with DAPI. (F) Densitometric quantification corresponding to Figure 1L. (G) Primary hepatocytes were treated with gradient LPS (0, 0.5, 1, 2, 5, 10 μg/mL) for 24 h, and relative cell viability was determined by CCK-8 assay. (H) Densitometric quantification corresponding to Figure 1O. (I) Protein levels of REST in hepatocyte in which *Rest* was either silenced or overexpressed. Densitometric quantification was shown on the right. Data are shown in mean ± SEM; each dot represents data from an individual sample; ns=not significant; *p<0.05; **p<0.01; ***p<0.001.


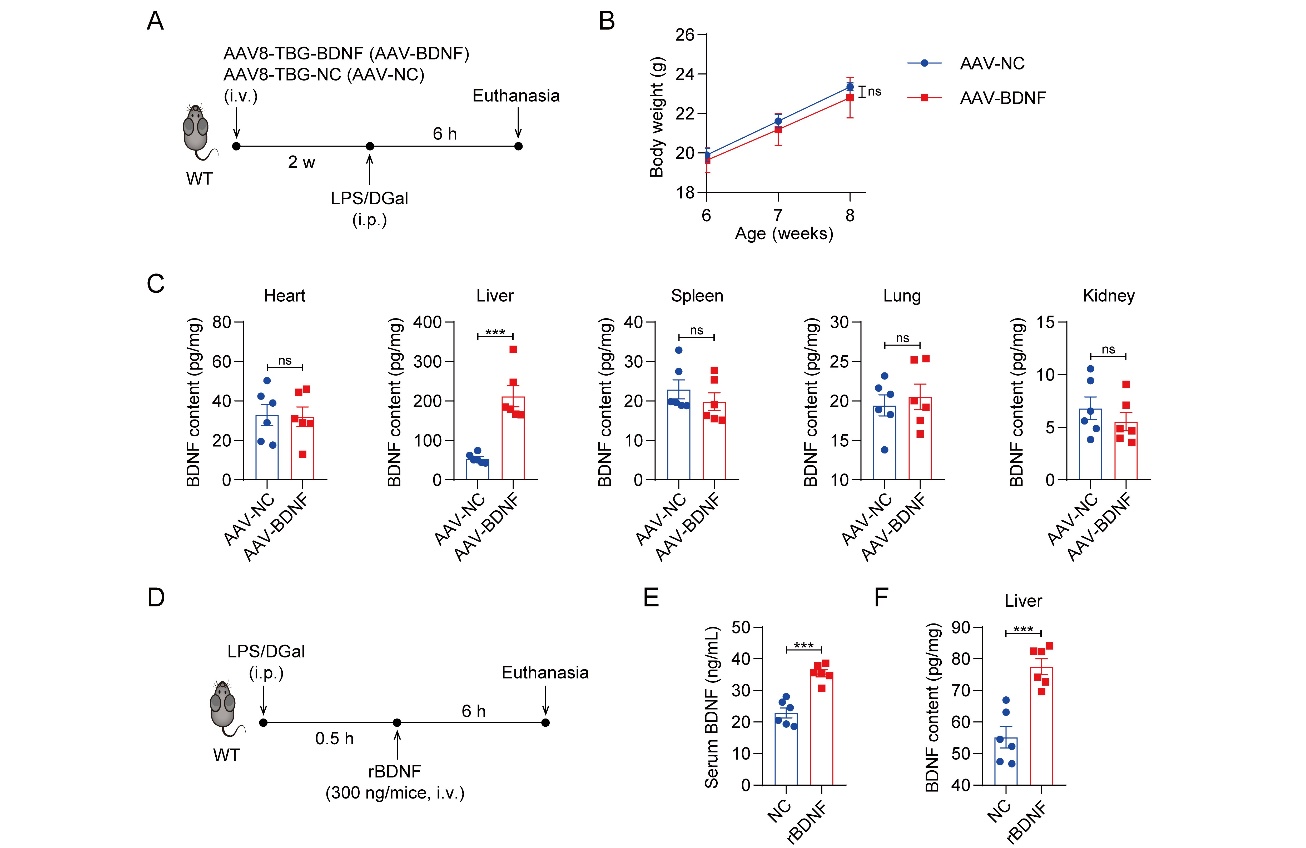
**Fig. S2. Experimental workflow in mice and quantification of BDNF levels across various organs.** (A) Schematic diagram of the experimental workflow. Hepatocyte-specific BDNF overexpression and secretion were achieved via tail vein injection of AAV8-TBG-sp-BDNF (AAV-BDNF) for 2 weeks, while AAV8-TBG-sp-NC (AAV-NC) served as the negative control. ALI/ALF was induced using LPS combined with DGal. (B) Body weight changings after AAV8 injection for 2 weeks. (C) BDNF content in heart, liver, spleen, lung, and kidney from AAV8 injected mice. (D) Schematic diagram of the experimental workflow. Recombinant BDNF protein (rBDNF) or vehicle control was administered to mice via tail vein injection following LPS/D-Gal treatment. (E) Serum level of BDNF in mice treated with or without rBDNF. (F) BDNF content in liver from mice treated with or without rBDNF. Data are shown in mean ± SEM; n=6 per group; ns=not significant; *p<0.05; **p<0.01; ***p<0.001.


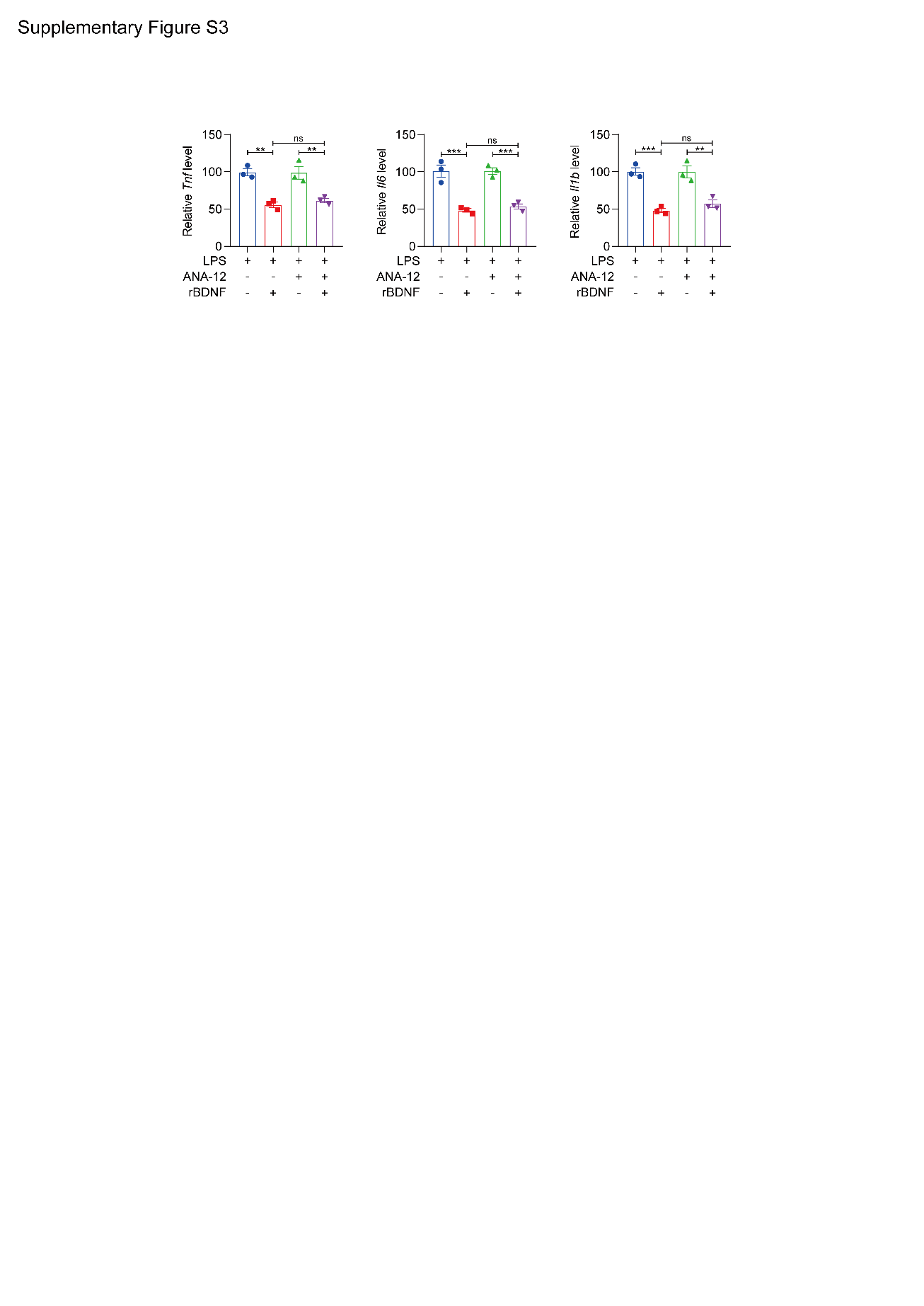


**Fig. S3. TrkB inhibitor ANA-12 does not abolish the anti-inflammatory effect of BDNF.** Macrophages were pretreated with ANA-12 (10 μM), rBDNF (100 ng/mL), or solvent control for 1 h and then stimulated with 500 ng/mL LPS for 12 h. mRNA levels of *Tnf*, *Il6*, and *Il1b* were detected by RT-qPCR. Data are shown in mean ± SEM; n=3 per group; ns=not significant; *p<0.05; **p<0.01; ***p<0.001.


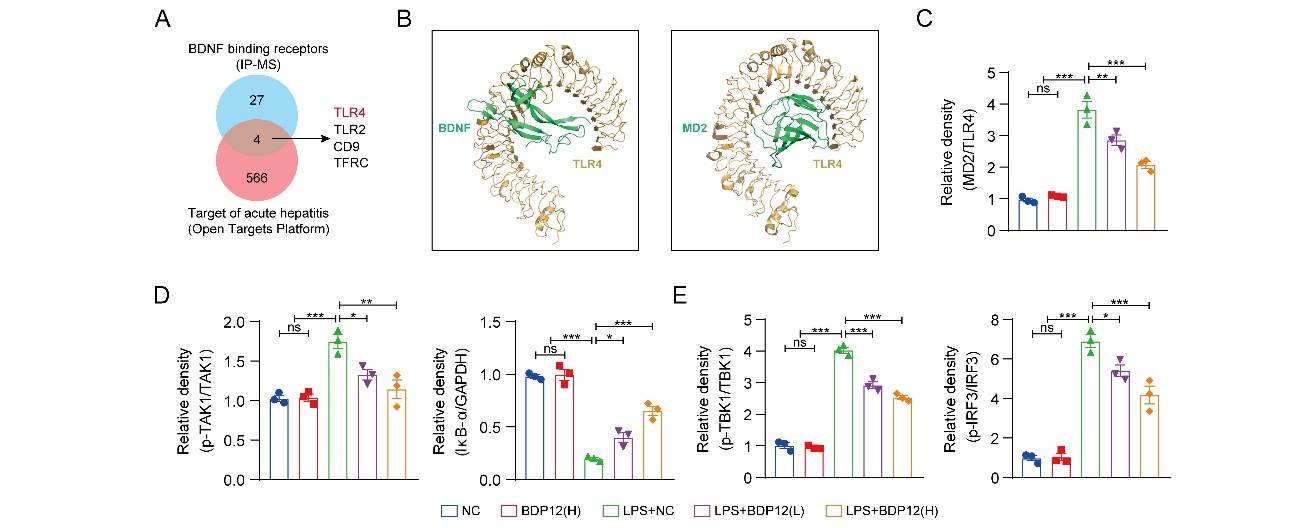
**Fig. S4. Identification of TLR4 as a binding receptor of BDNF by IP-MS and comparison of the structural conformations of BDNF-TLR4 and MD2-TLR4 complexes.** (A) Venn diagram showing that among the 31 potential binding receptors of BDNF, TLR4 was identified as a BDNF-targeted receptor that highly associated with acute hepatitis. (B) Structural conformations of the BDNF-TLR4 complex and the MD2-TLR4 complex (PDB: 3FXI). (C) Densitometric quantification corresponding to Figure 5F. (D) Densitometric quantification corresponding to Figure 5G. (E) Densitometric quantification corresponding to Figure 5H. Data are shown in mean ± SEM; n=3 per group; ns=not significant; *p<0.05; **p<0.01; ***p<0.001.


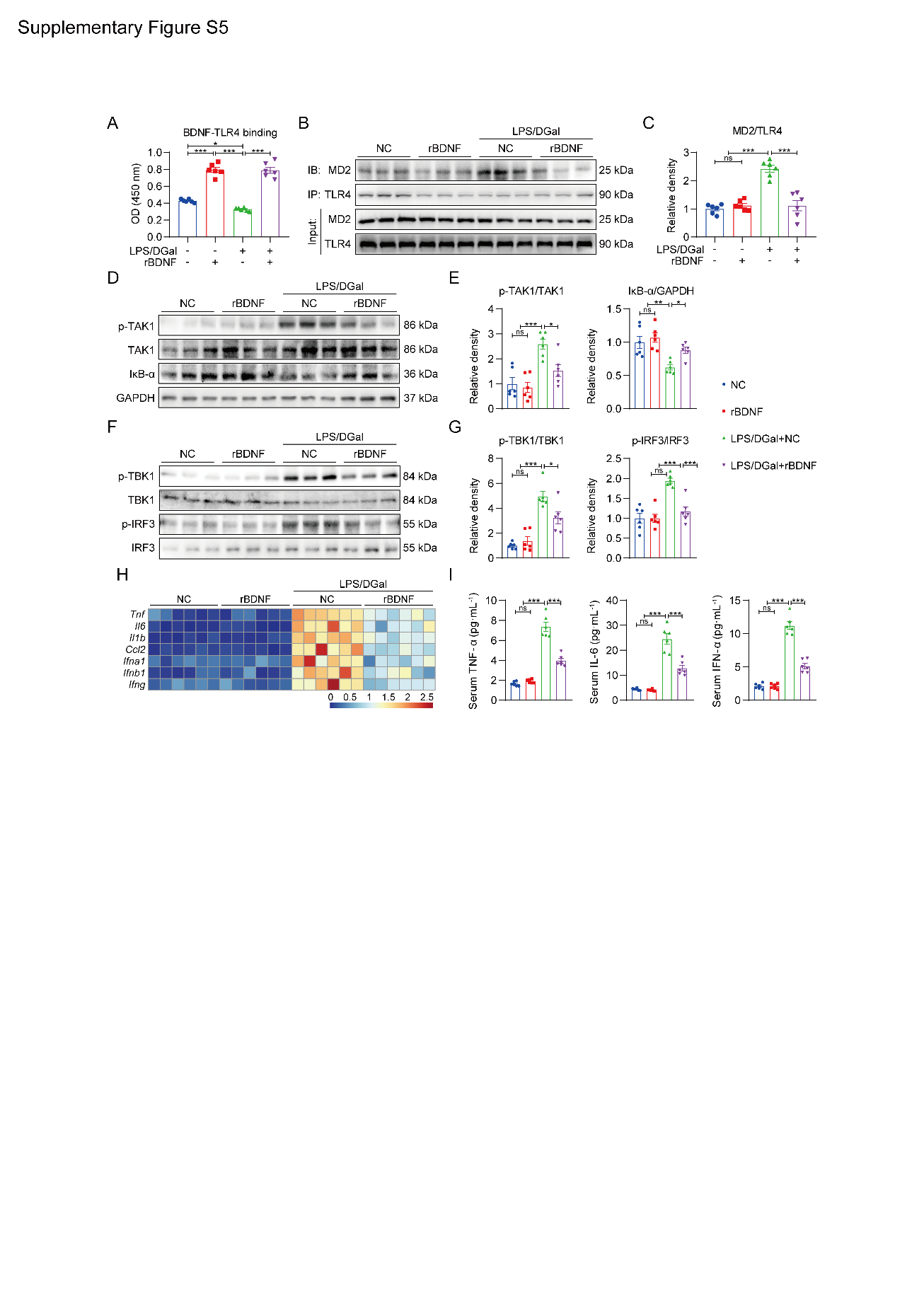
 **Fig. S5. rBDNF treatment disrupts the TLR4 pathway and inflammation response in ALI/ALF mice.** (A) The concentration of the BDNF-TLR4 complex in the liver was determined by ELISA. (B) The level of MD2-TLR4 complex formation in liver tissues was assessed by co-immunopercipetation. (C) Densitometric quantification of panel B. (D) Activation of the TAK1-NFκB pathway was assessed by probing for phosphorylated TAK1 and IκB-α, with unphosphorylated proteins and GAPDH as controls. (E) Densitometric quantification of panel D. (F) Activation of the TBK1-IRF3 pathway was evaluated by probing for phosphorylated TBK1 and IRF3, with unphosphorylated proteins as controls. (G) Densitometric quantification of panel F. (H) Heatmap showing mRNA levels of pro-inflammatory factors *Tnf*, *Il6*, *Il1b*, *Ccl2*, *Ifna1*, *Ifnb1*, and *Ifng* in liver tissues. (I) Serum levels of TNF-α, IL-6, and IFN-α in the mice. Data are shown in mean ± SEM; n=6 per group; ns=not significant; *p<0.05; **p<0.01; ***p<0.001.


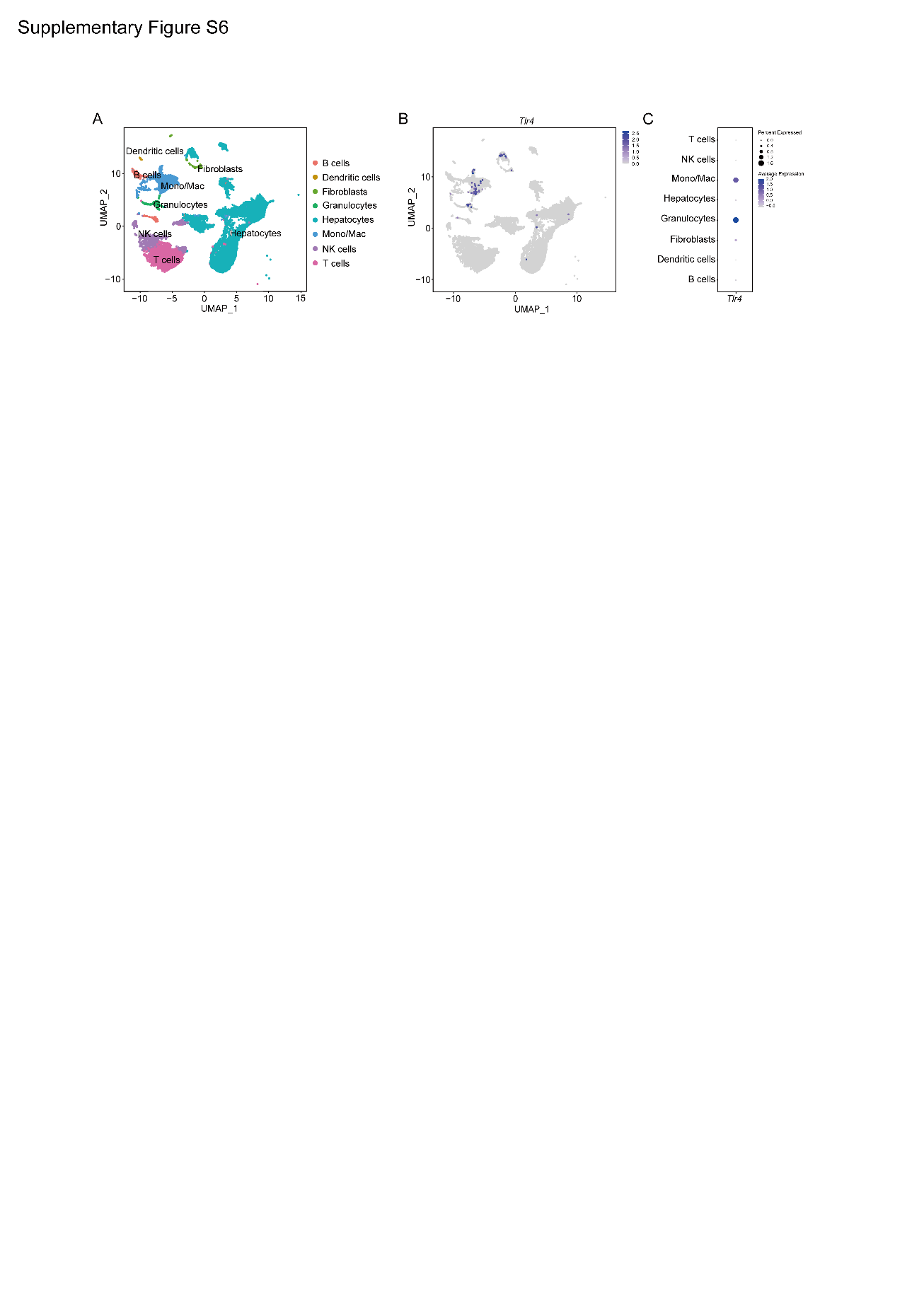


**Fig. S6. Identification of the cellular source of TLR4 in the liver.** (A) UMAP plot showing 8 cell clusters in the mouse liver. (B) Feature plot illustrating the single-cell expression distribution of *Tlr4* in mouse liver. (C) Dot plot showing *Tlr4* expression across the 8 liver cell clusters.


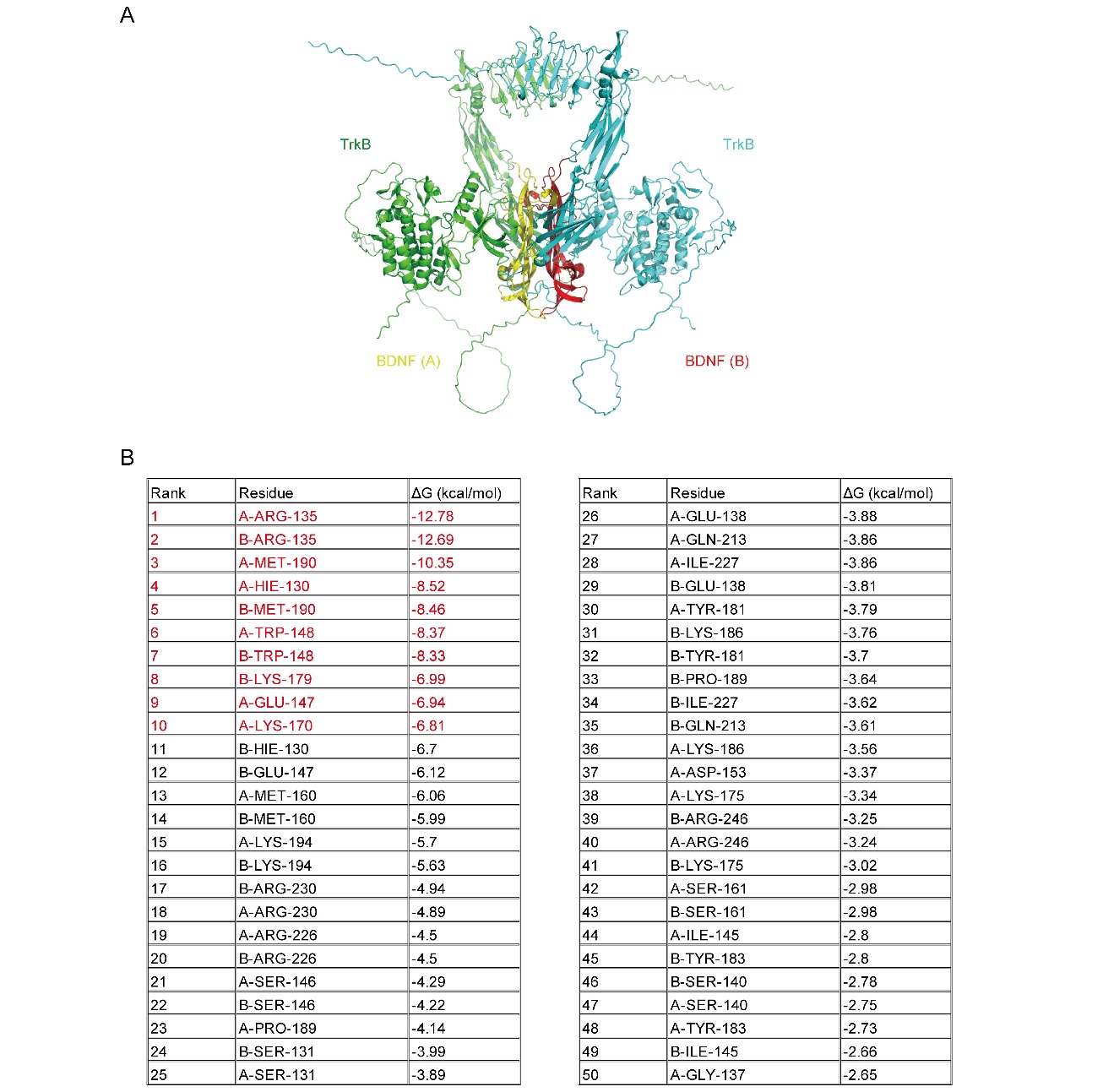


**Fig. S7. Conformation of the BDNF-TrkB complex and binding free energy of BDNF residues.** (A) The tetrameric structure formed by two BDNF molecules and two TrkB molecules as predicted by AlphaFold3. (B) The top 50 BDNF amino acid residues contributing to the BDNF-TrkB complex, as calculated by HawkDock using the VD-MM/GBSA method. "A" and "B" represent the different BDNF molecules in the BDNF homodimer. The top 10 residues are highlighted in red.


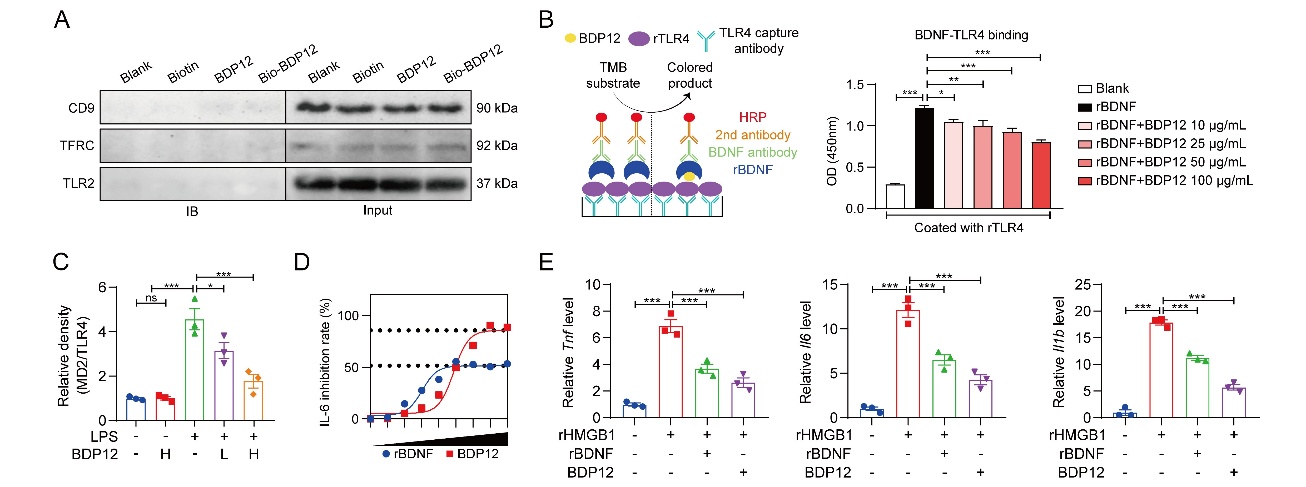
**Fig. S8. BDP12 competes with BDNF for binding to TLR4, and both BDNF and BDP12 inhibit HMGB1-induced inflammation.** (A) Biotinylated pull-down assays were performed to assess the interaction between BDP12 and CD9, TFRC, or TLR2. (B) Schematic illustration of the experimental design used to verify the ability of BDP12 to compete with BDNF for TLR4 binding using ELISA. ELISA analyses show a dose-dependent decrease in the binding of BDNF to TLR4 upon treatment with BDP12. (C) Densitometric quantification corresponding to Figure 7H. (D) Macrophages were treated with increasing concentrations of rBDNF (0, 3.125, 6.25, 12.5, 25, 50, 100, 200, 400 ng/mL) or BDP12 (0, 0.0625, 0.125, 0.25, 0.5, 1, 2, 4, 8 µM) for 1 hour, followed by stimulation with LPS (500 ng/mL) for 24 hours. IL-6 levels in the medium were measured by ELISA, and inhibition rates were calculated. (E) Macrophages were pretreated with rBDNF (100 ng/mL), BDP12 (4 μM), or solvent control for 1 h and then stimulated with 100 ng/mL recombinant HMGB1 (rHMGB1) for 12 h. mRNA levels of *Tnf*, *Il6*, and *Il1b* were detected by RT-qPCR. Data are shown in mean ± SEM; n=3 per group; ns=not significant; *p<0.05; **p<0.01; ***p<0.001.


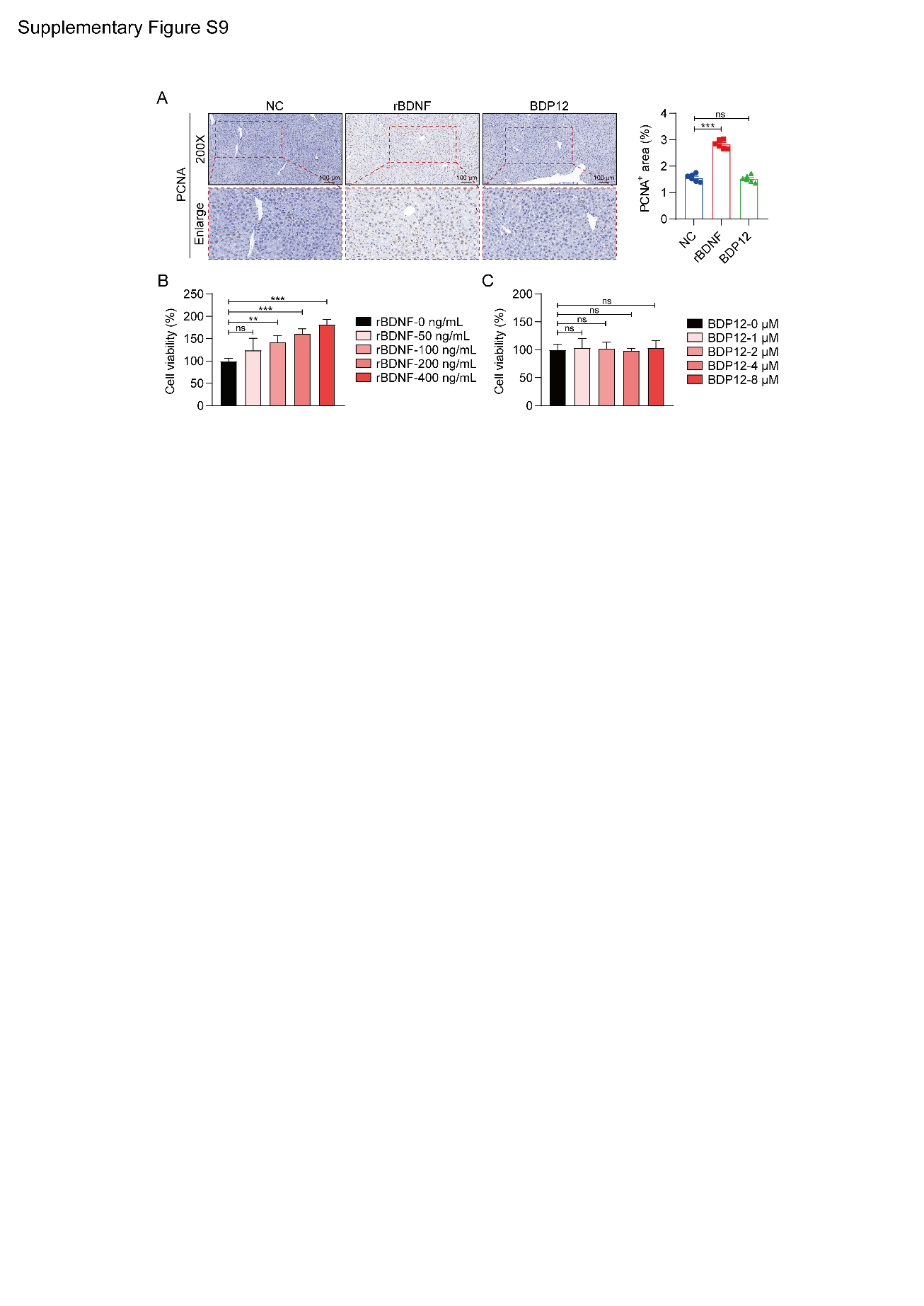


**Fig. S9. Differential proliferative potential of BDNF and BDP12 in the liver and in HepG2 cells.** (A) Mice treated with rBDNF or BDP12 for 3 d underwent immunohistochemistry staining for PCNA in liver tissues. Quantification of PCNA-positive area was shown on the right. (B) HepG2 cells were treated with gradient rBDNF (0, 50, 100, 200, 400 ng/mL) for 24 h, and relative cell viability was determined by CCK-8 assay. (C) HepG2 cells were treated with gradient BDP12 (0, 1, 2, 4, 8 μM) for 24 h, and relative cell viability was determined by CCK-8 assay. Data are shown in mean ± SEM; n=6 per group; ns=not significant; *p<0.05; **p<0.01; ***p<0.001.


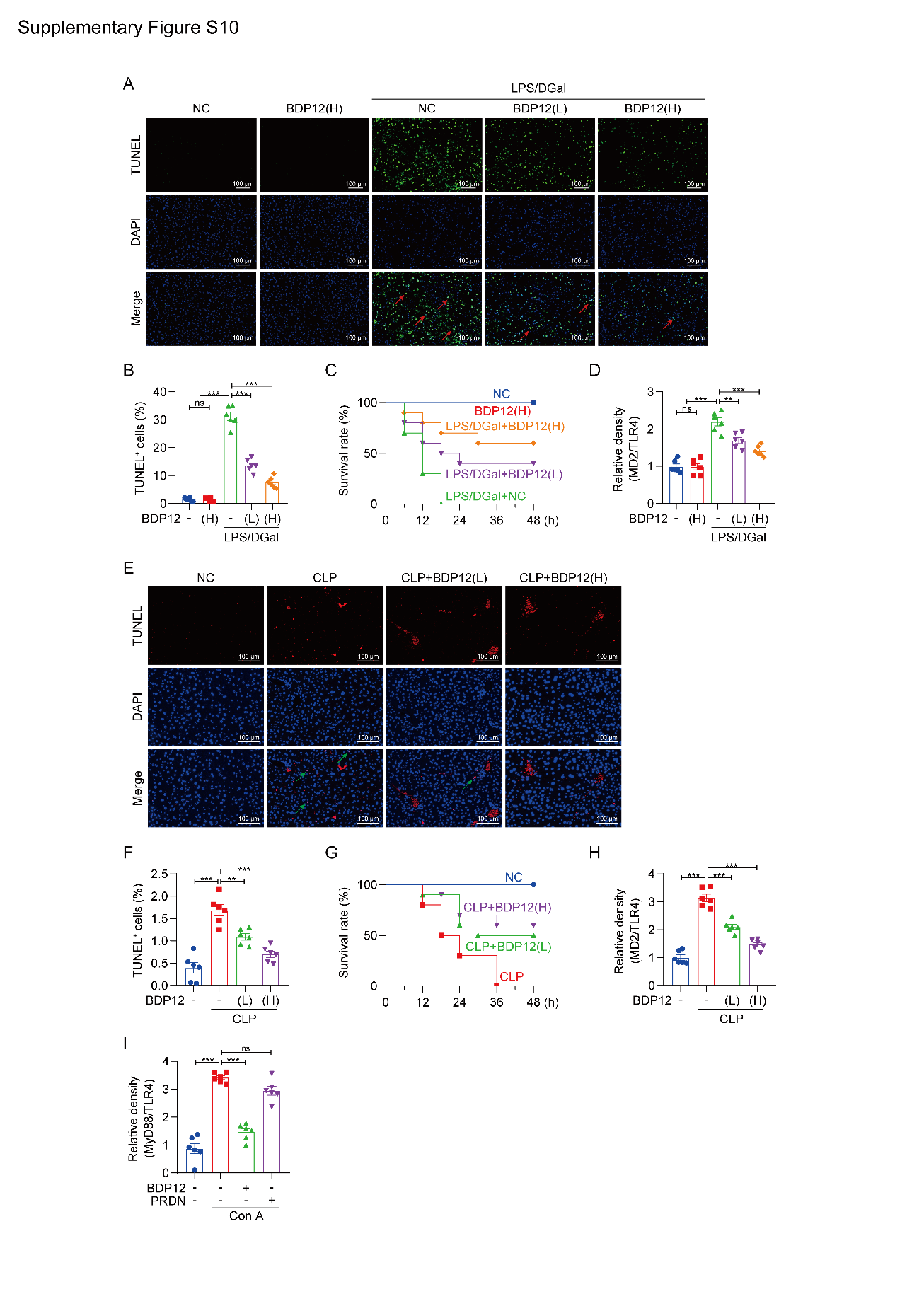


**Fig. S10. TUNEL staining and survival analysis of BDP12 in different ALI/ALF models.** (A) Representative images of TUNEL staining in liver sections from LPS/DGal-induced mice receiving either vehicle or BDP12 treatment. TUNEL-positive nuclei (green) indicate apoptotic cells. Nuclei were counterstained with DAPI. (B) Quantification of TUNEL-positive cells shown in panel A. (C) Survival curve of mice receiving either vehicle or BDP12 treatment in the LPS/DGal-induced lethal model. (D) Densitometric quantification corresponding to Figure 8J. (E) Representative images of TUNEL staining in liver sections from CLP-operated mice receiving either vehicle or BDP12 treatment. TUNEL-positive nuclei (red) indicate apoptotic cells. Nuclei were counterstained with DAPI. (F) Quantification of TUNEL-positive cells shown in panel E. (G) Survival curve of mice receiving either vehicle or BDP12 treatment in the CLP-induced lethal model. (H) Densitometric quantification corresponding to Figure 8V. (I) Densitometric quantification corresponding to Figure 9I. Data are shown in mean ± SEM; n=6 per group; ns=not significant; *p<0.05; **p<0.01; ***p<0.001.


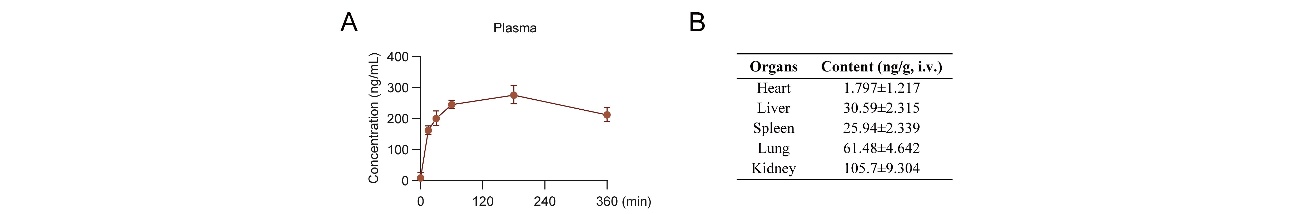


**Fig. S11. Pharmacokinetic and tissue distribution analyses of BDP12.** (A) Mice were administered BDP12 by intraperitoneal injection, and blood samples were collected at 0, 15, 30, 60, 180, and 360 min after administration. Plasma concentrations of BDP12 were measured by LC-MS/MS. (B) Mice were intraperitoneally injected with BDP12 once daily for three consecutive days. Two hours after the third injection, tissues including heart, liver, spleen, lung, and kidney were collected, and BDP12 concentrations were determined by LC-MS/MS. Data are shown in mean ± SEM; n=3 per group.

**Supplementary Tables**

**Table S1. Primers used for real-time qPCR assay.**

| **Gene** | **Species** | **Forward sequence** | **Reverse sequence** |
| --- | --- | --- | --- |
| *Bdnf* | mouse | GGCTGACACTTTTGAGCACGTC | CTCCAAAGGCACTTGACTGCTG |
| *Il6* | mouse | AGTTGCCTTCTTGGGACTGA | TCCACGATTTCCCAGAGAAC |
| *Il1b* | mouse | GCAACTGTTCCTGAACTCAACT | ATCTTTTGGGGTCCGTCAACT |
| *Tnf* | mouse | CCCTCACACTCAGATCATCTTCT | GCTACGACGTGGGCTACAG |
| *Ccl2* | mouse | CTTCTGGGCCTGCTGTTCA | CCAGCCTACTCATTGGGATCA |
| *Ifnb1* | mouse | CAGCTCCAAGAAAGGACGAAC | GGCAGTGTAACTCTTCTGCAT |
| *Ifng* | mouse | ATGAACGCTACACACTGCATC | CCATCCTTTTGCCAGTTCCTC |
| *Icam1* | mouse | GTGATGCTCAGGTATCCATCCA | CACAGTTCTCAAAGCACAGCG |
| *Vcam1* | mouse | AGTTGGGGATTCGGTTGTTCT | CCCCTCATTCCTTACCACCC |
| *Actb* | mouse | GGCTGTATTCCCCTCCATCG | CCAGTTGGTAACAATGCCATGT |
