# Supplementary material for: Hepatocyte BDNF Acts as a Novel Immune Checkpoint to Restrain TLR4‐Mediated Acute Hepatitis
Source: Adv Sci (Weinh). 2026 Mar 25;13(32):e21164. doi: 10.1002/advs.202521164 (PMC13252628; doi:10.1002/advs.202521164)

Figure 1J

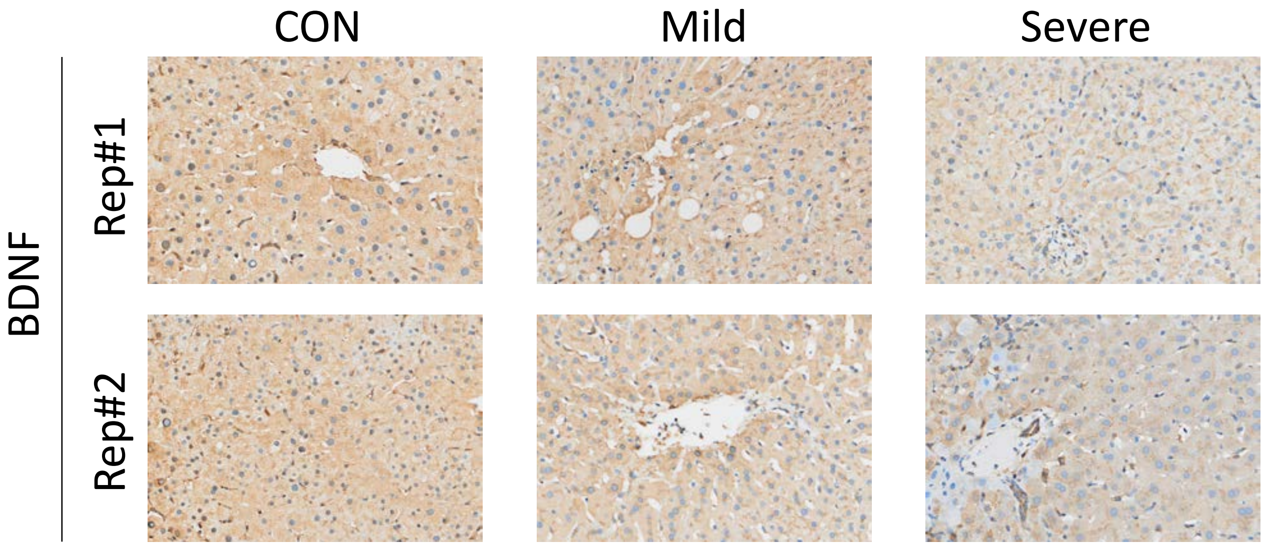

Figure 2E, 2H, 2N

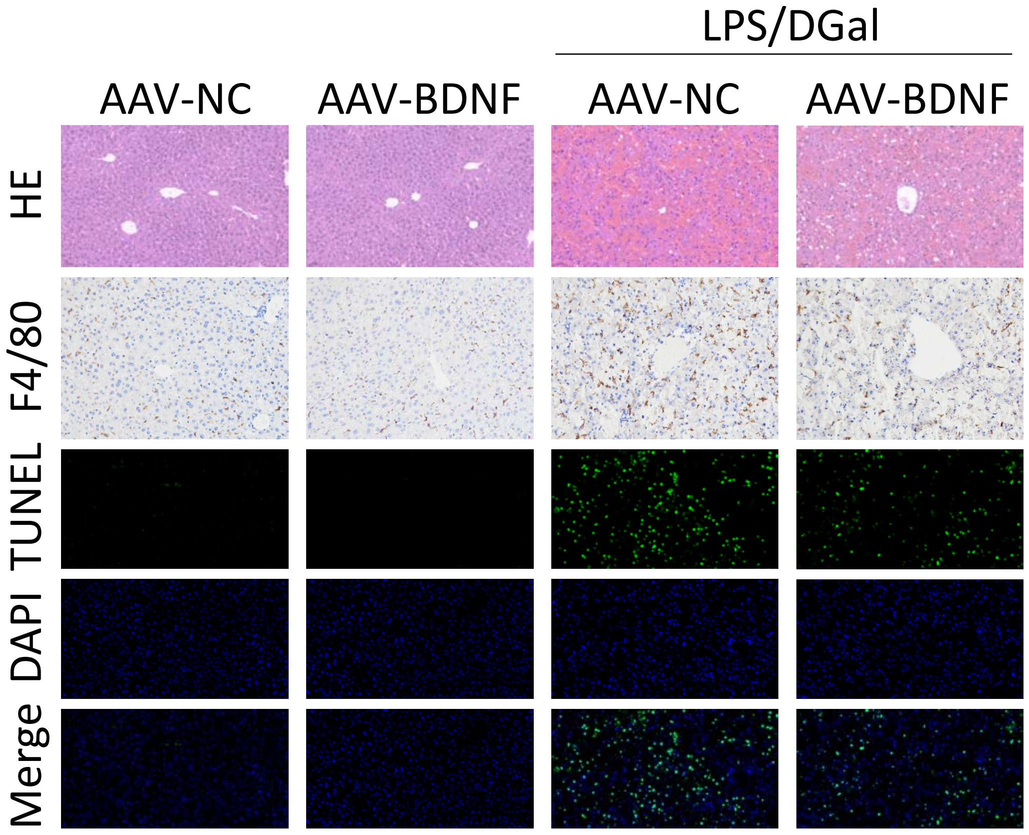

Figure 3E, 3H, 3N

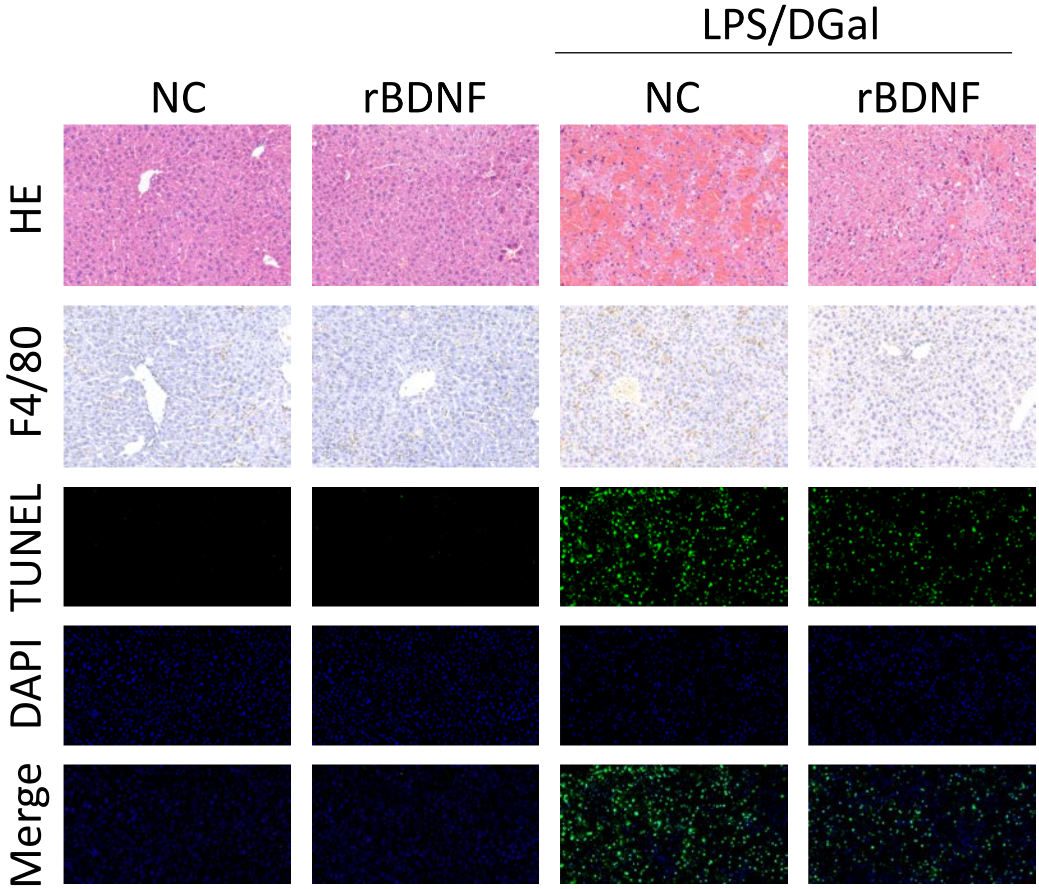

Figure 4I

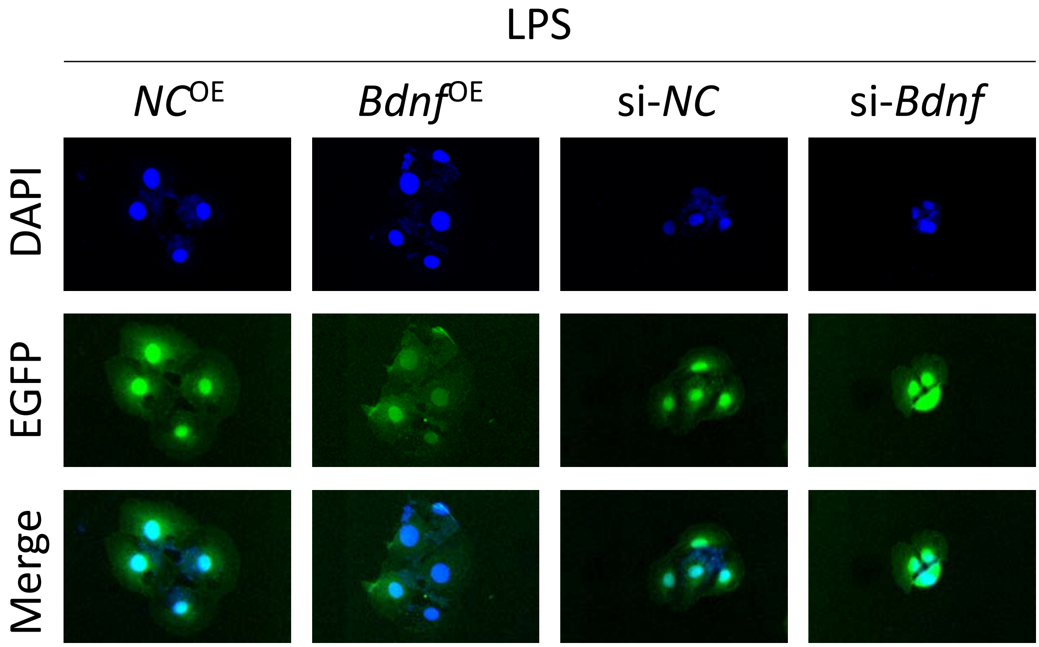

Figure 4J

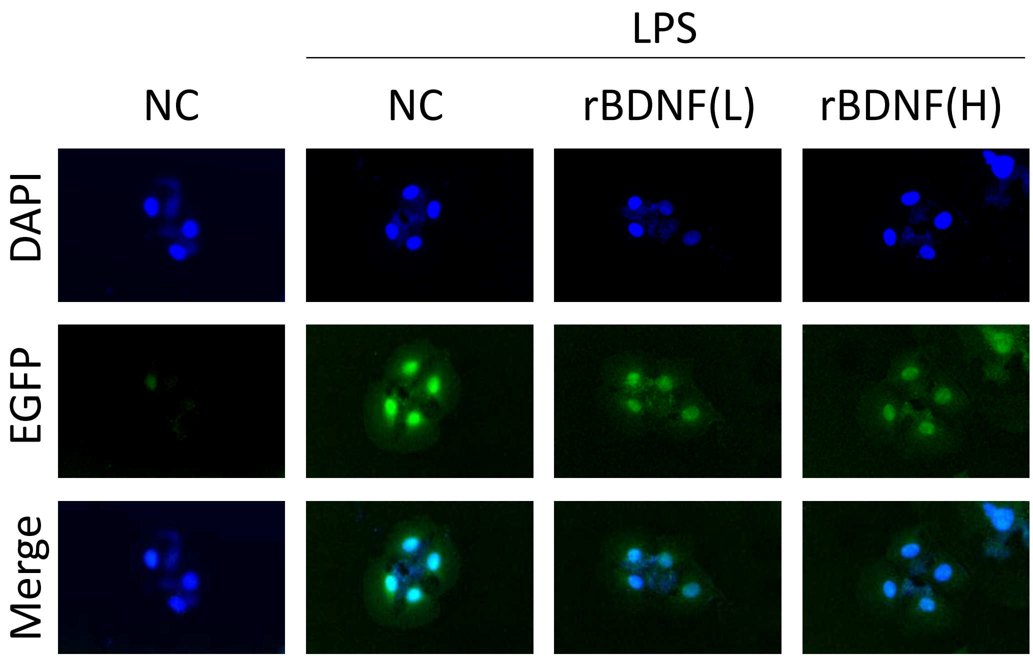

Figure 5D

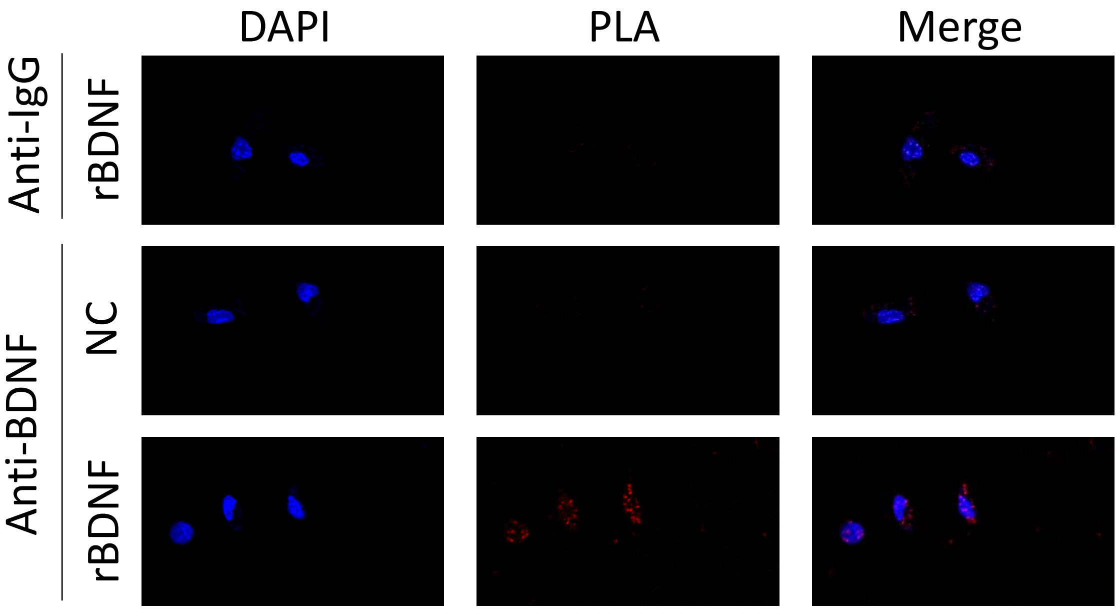

Figure 6F, 6I, 6M

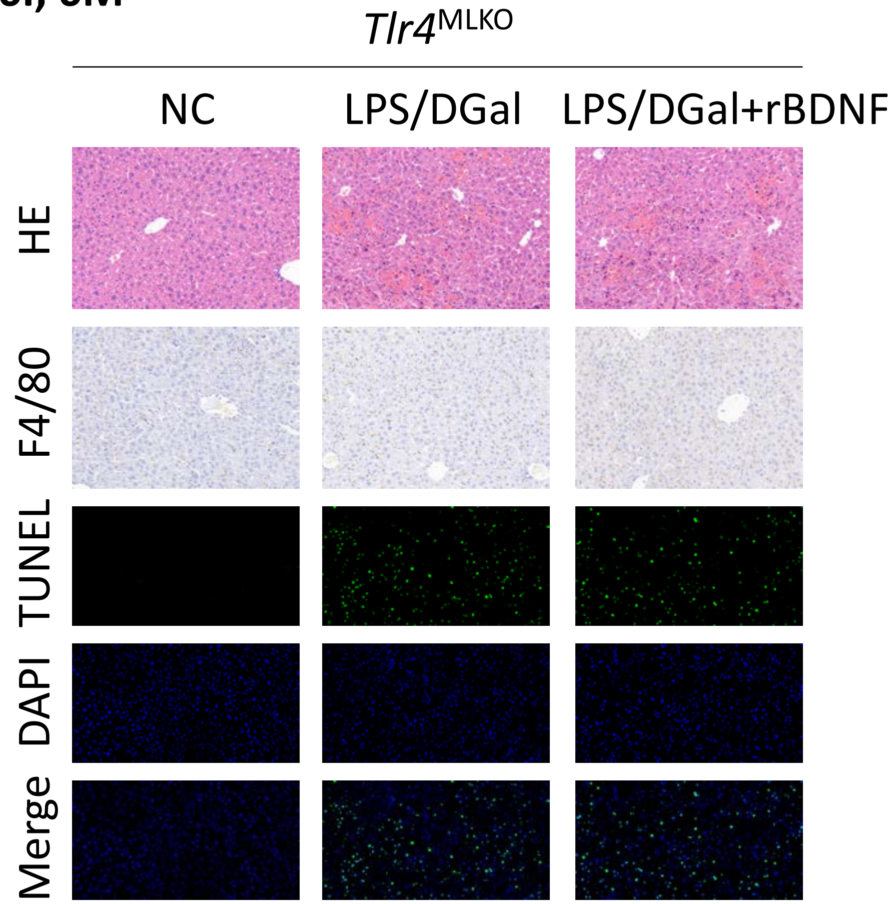

Figure 7F

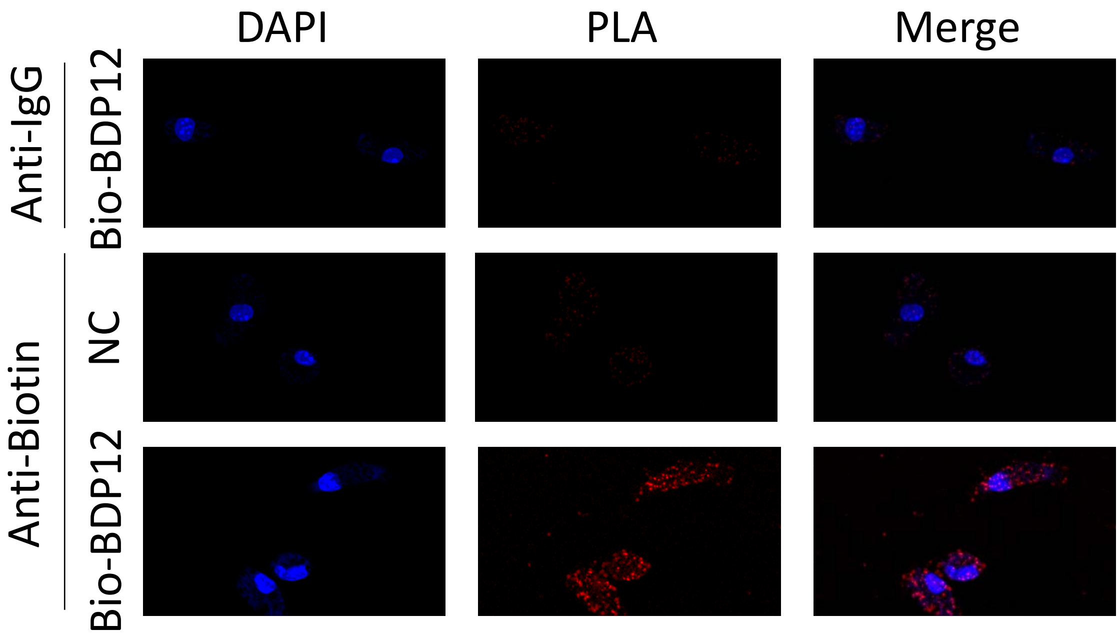

Figure 7G

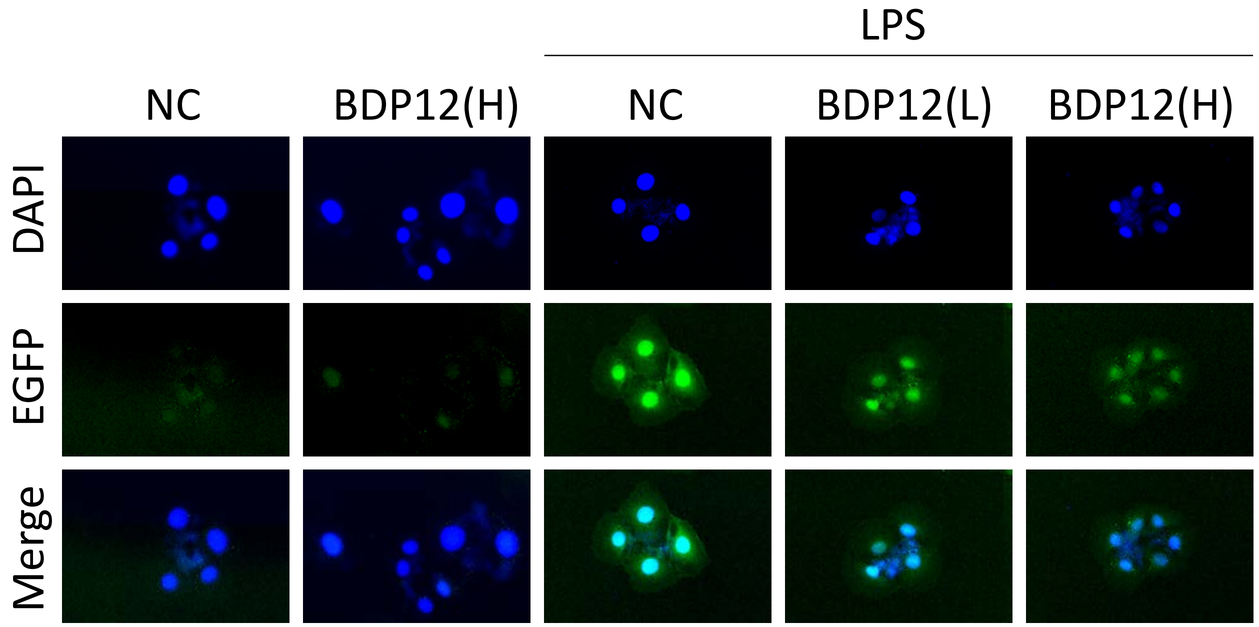

Figure 8B, 8H

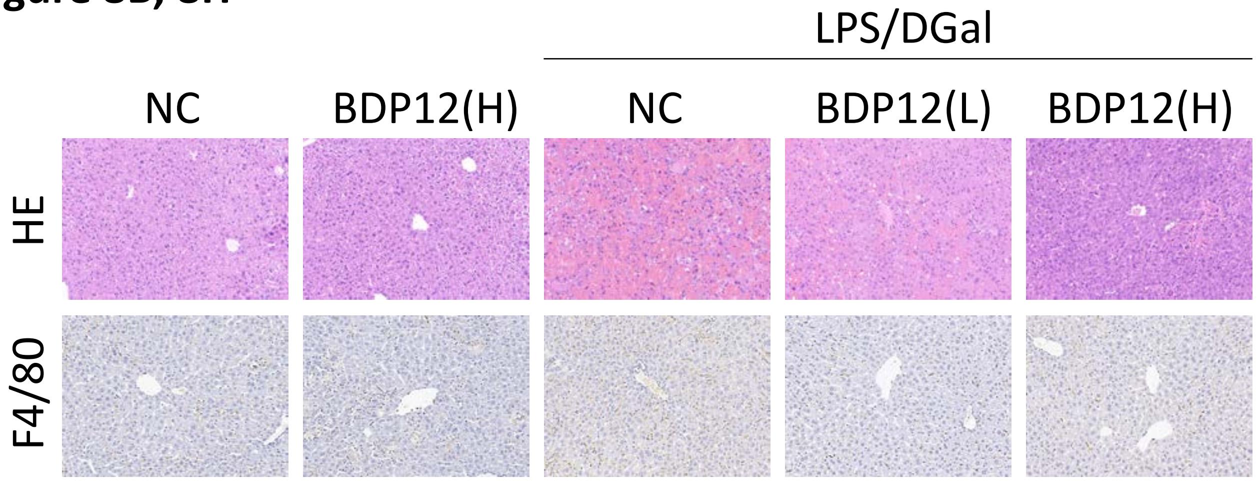

Figure 8N, 8T

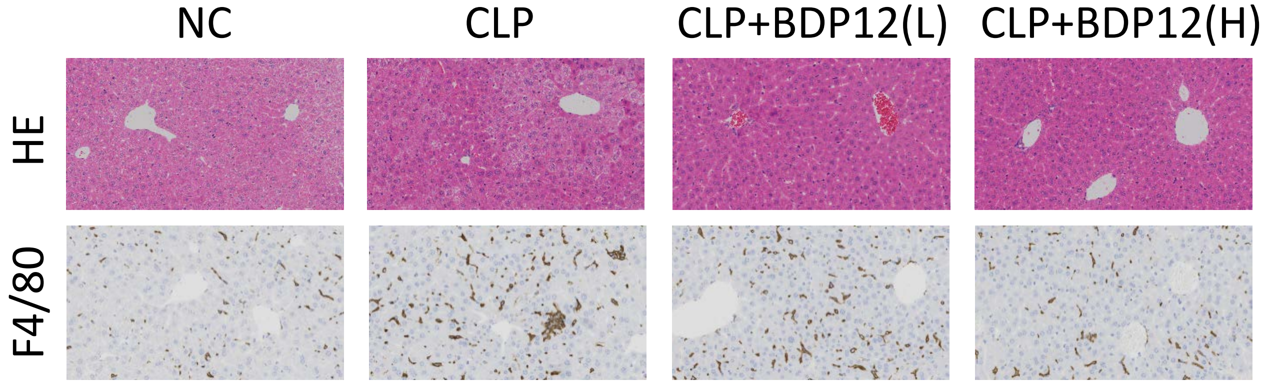

Figure 9B, 9G

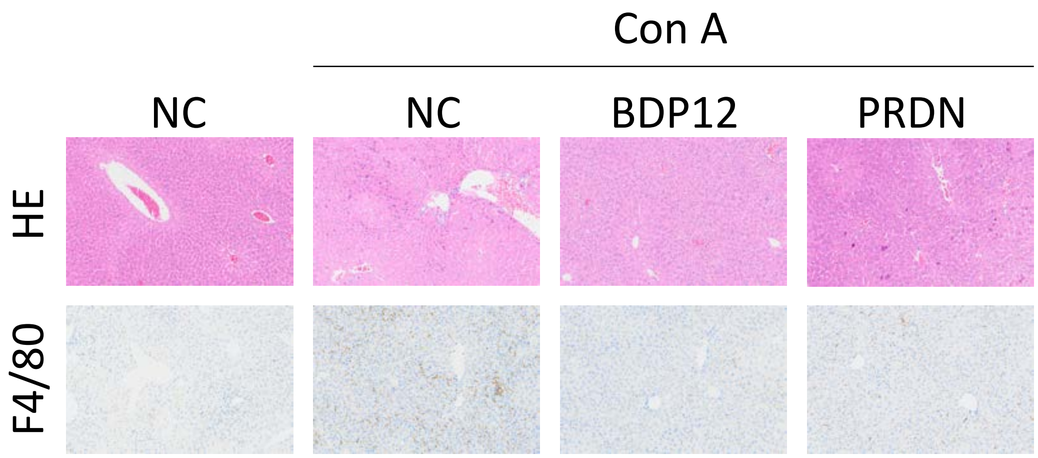

Supplementary Figure S1D

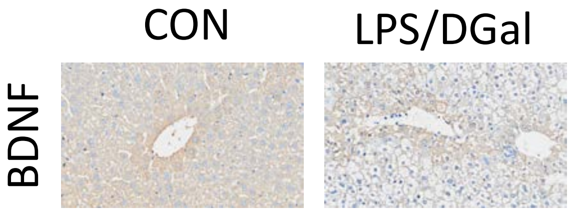

Supplementary Figure S1E

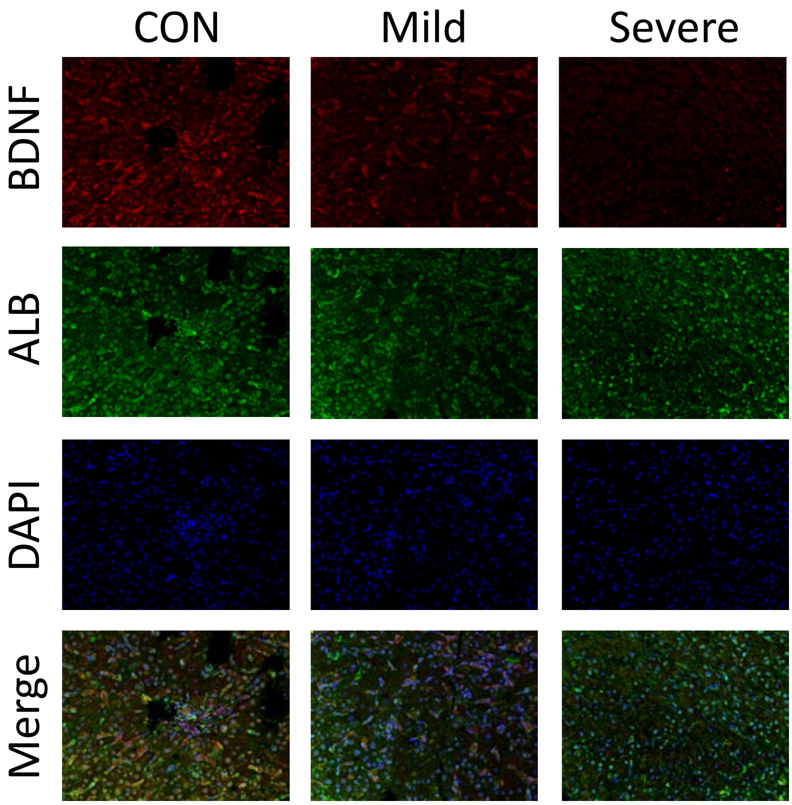

Supplementary Figure S9

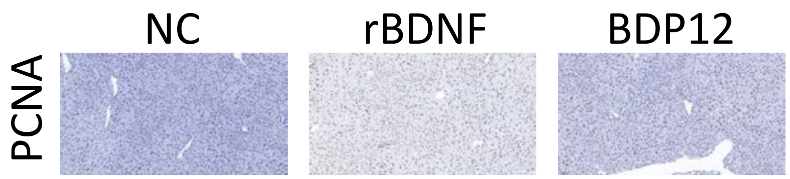

Supplementary Figure S10A

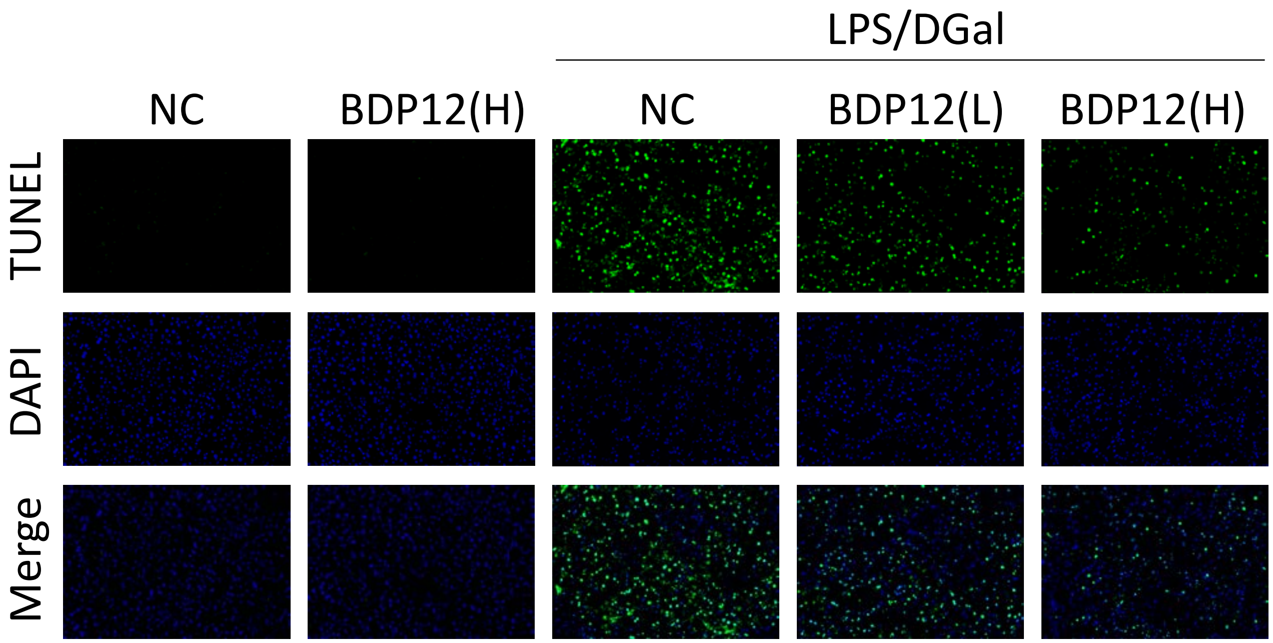

Supplementary Figure S10E

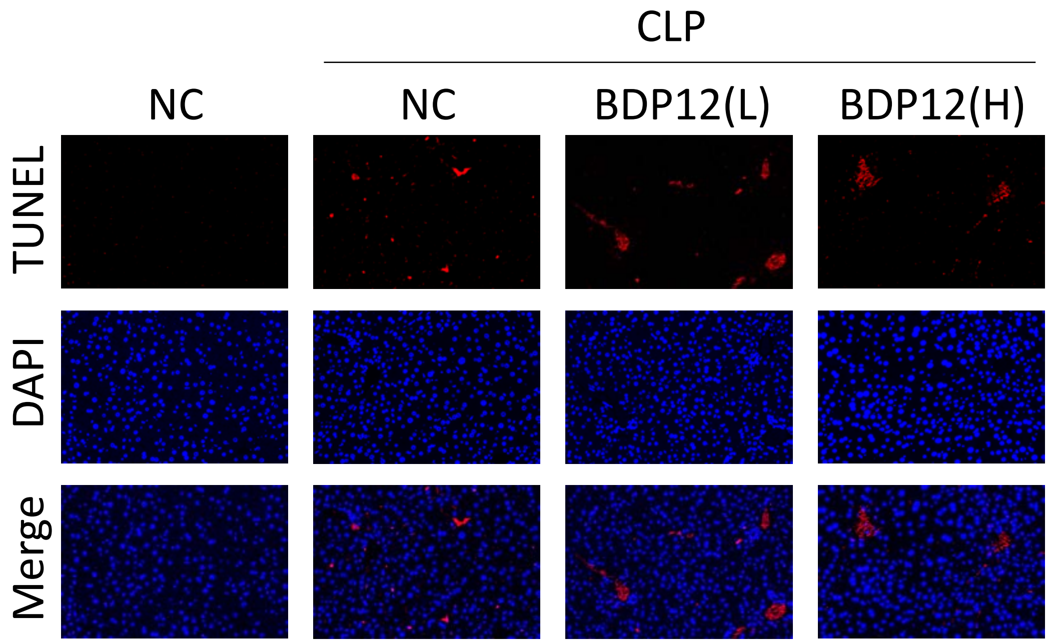

Unedited gel for Figure 1E

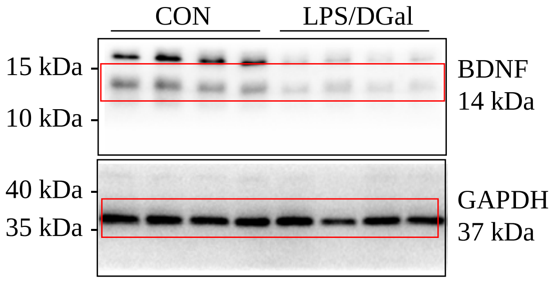

Unedited gel for Figure 1L

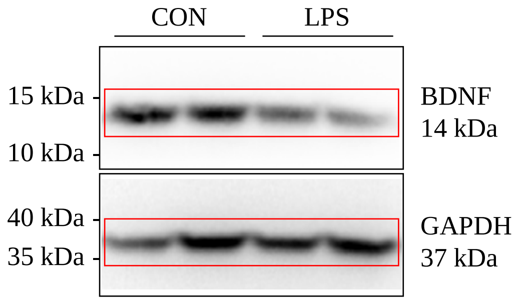

Unedited gel for Figure 1O

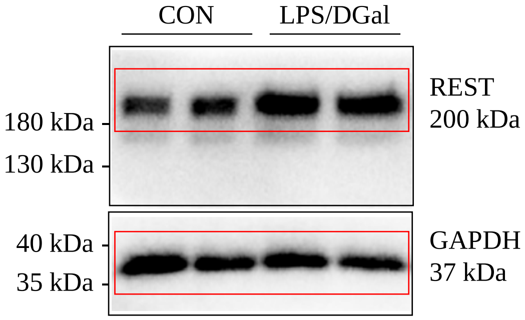

Liver lysates

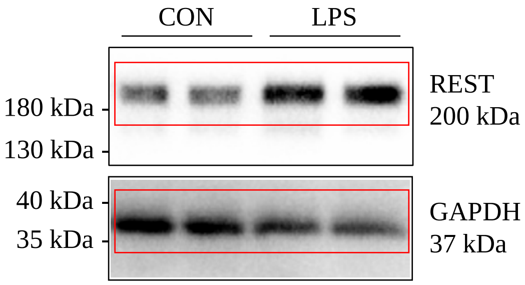

Hepatocytes

Unedited gel for Figure 2L

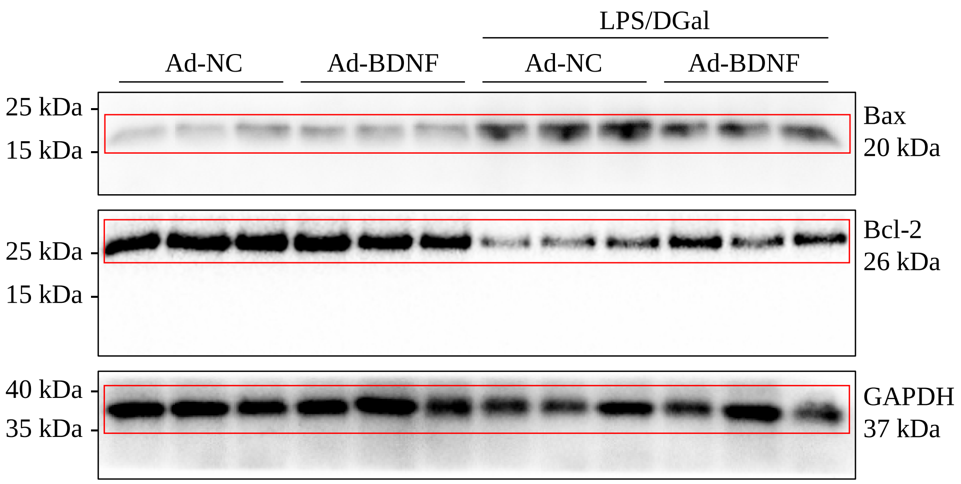

Unedited gel for Figure 3L

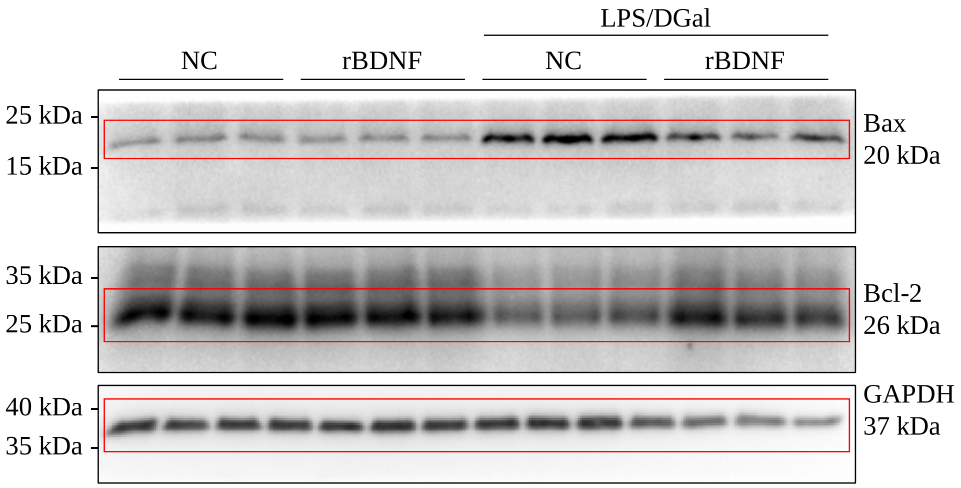

Unedited gel for Figure 5B

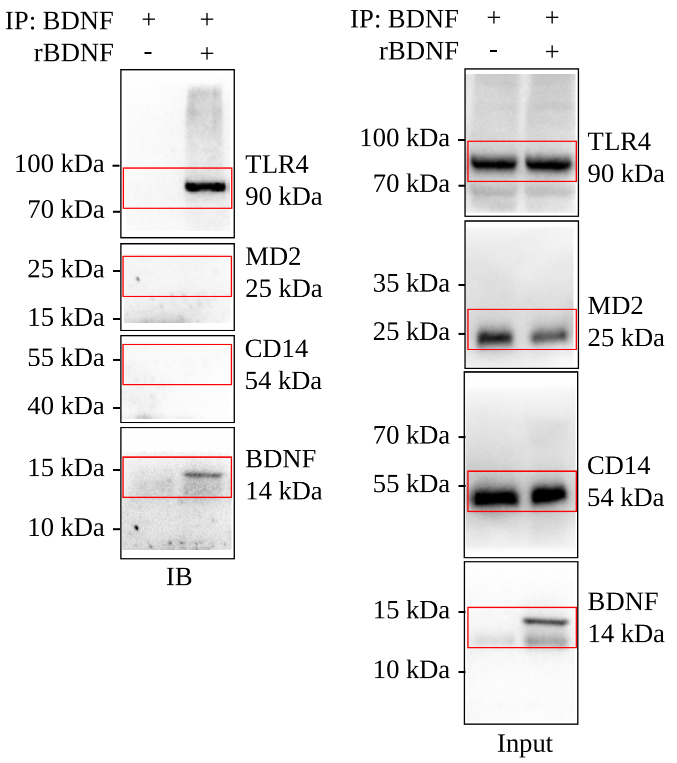

Unedited gel for Figure 5F

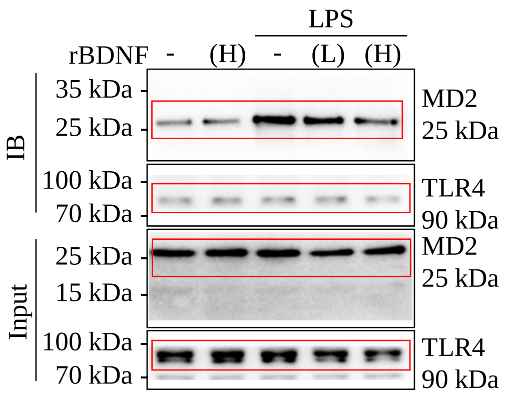

Unedited gel for Figure 5G

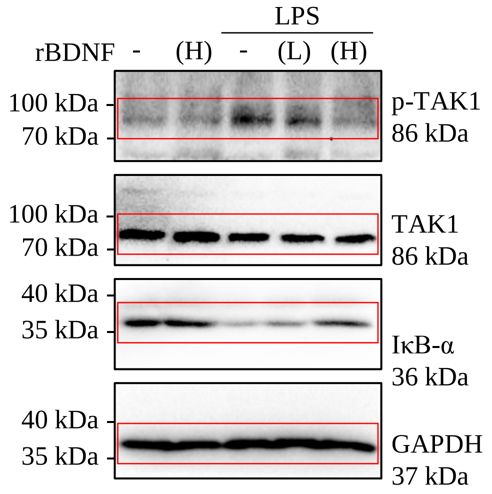

Unedited gel for Figure 5H

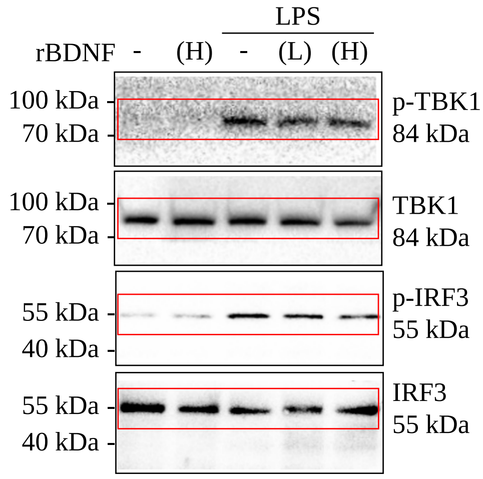

Unedited gel for Figure 6O

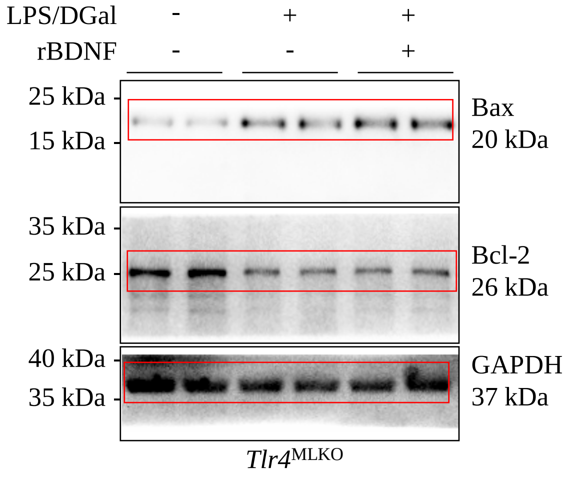

Unedited gel for Figure 7E

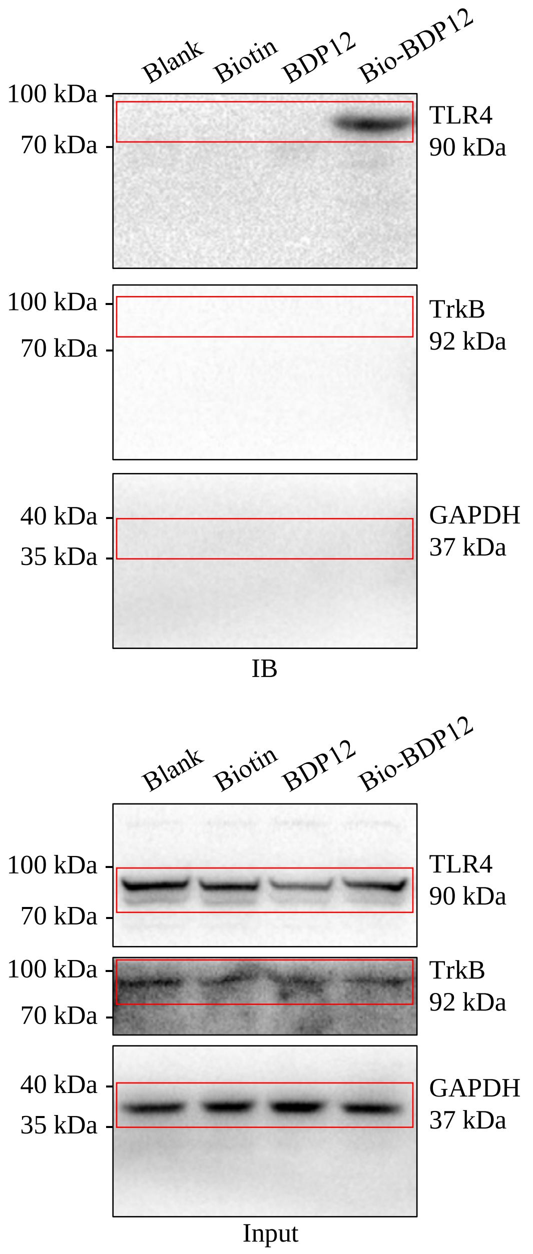

Unedited gel for Figure 7H

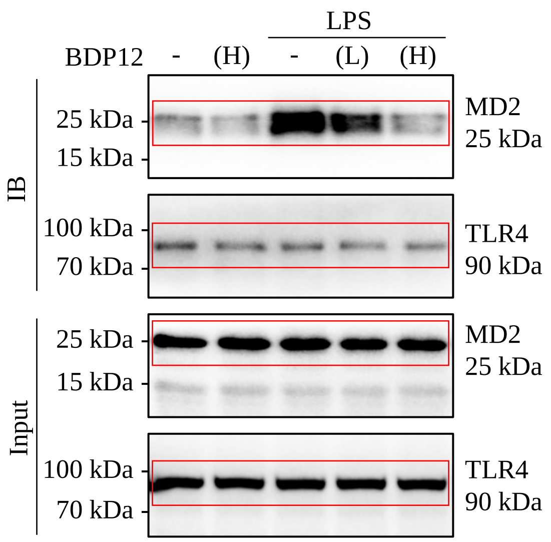

Unedited gel for Figure 8J

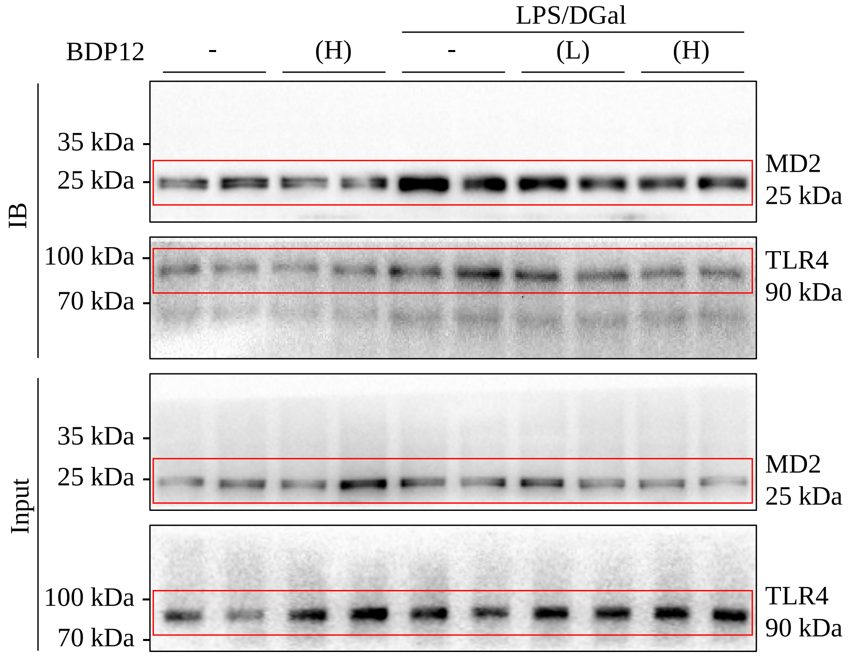

Unedited gel for Figure 8V

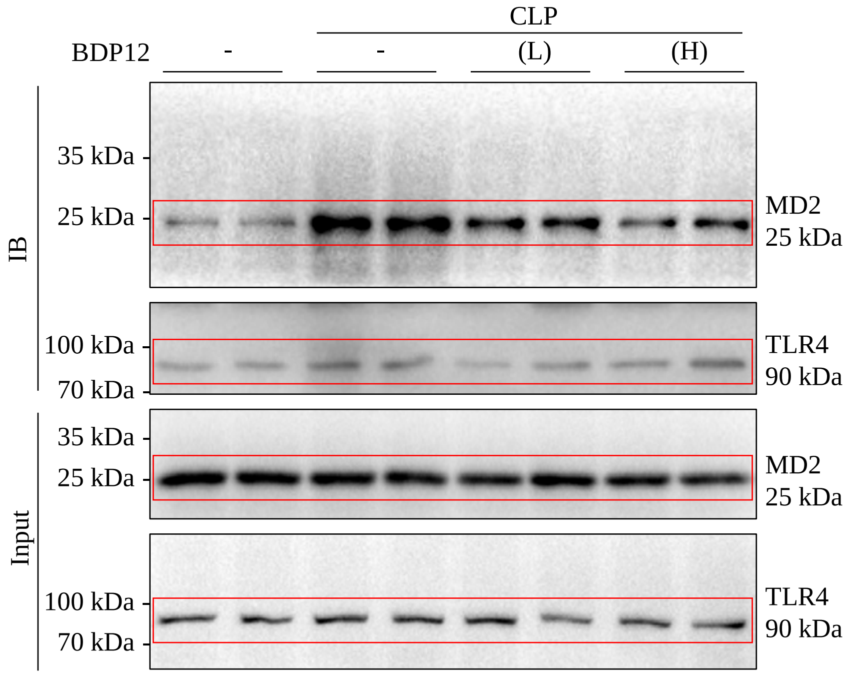

Unedited gel for Figure 9I

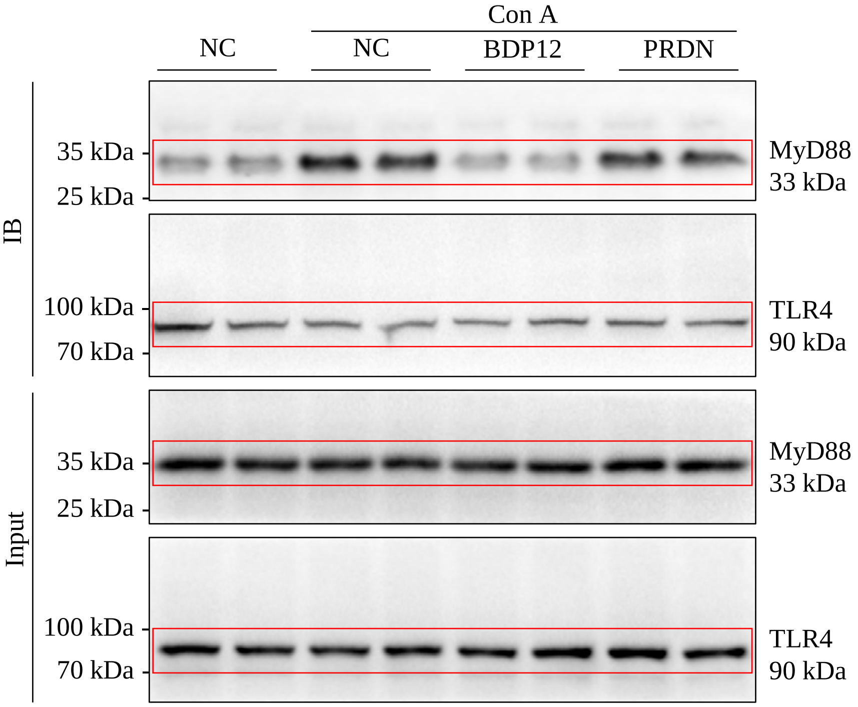

Unedited gel for Supplementary Figure S1I

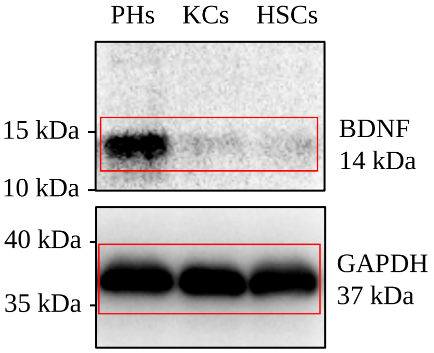

Unedited gel for Supplementary Figure S1I

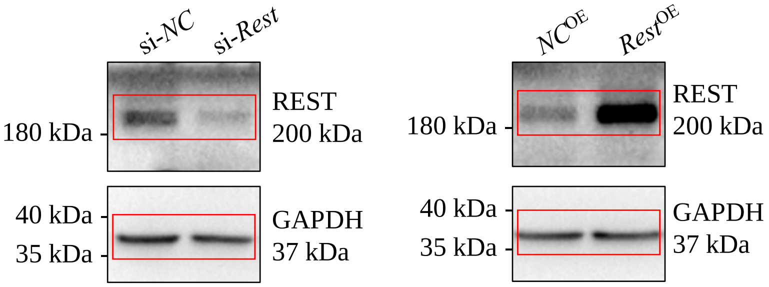

Unedited gel for Supplementary Figure S5B

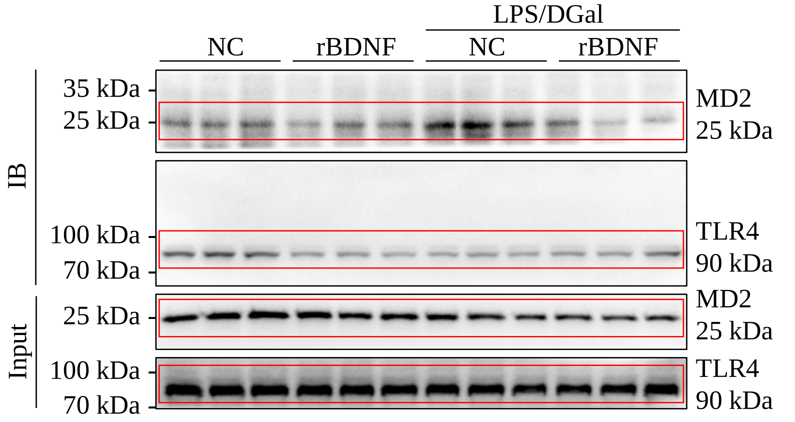

Unedited gel for Supplementary Figure S5D

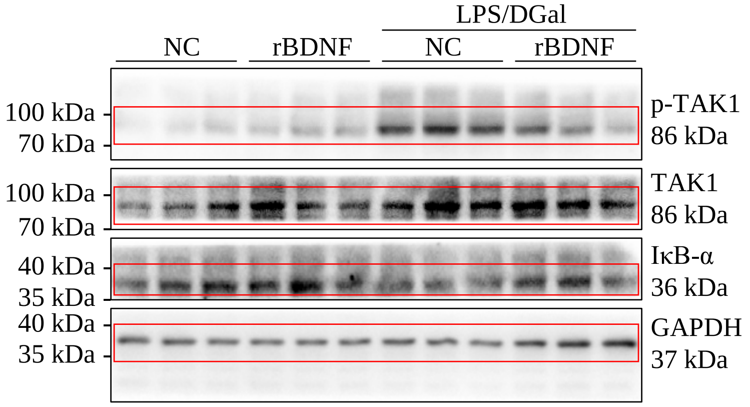

Unedited gel for Supplementary Figure S5F

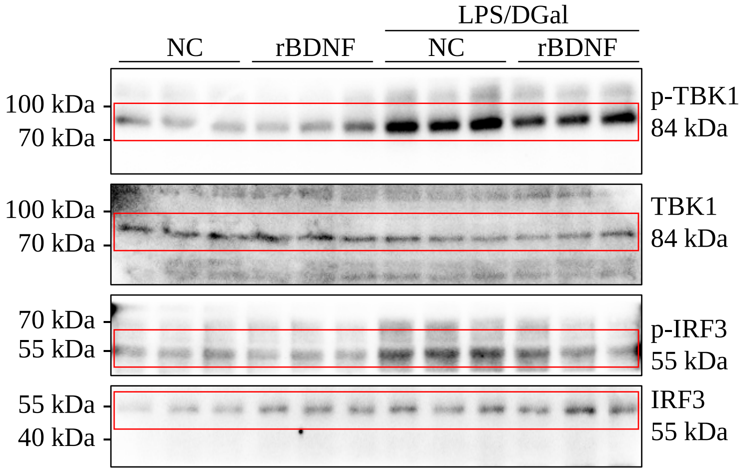

Unedited gel for Supplementary Figure S8A

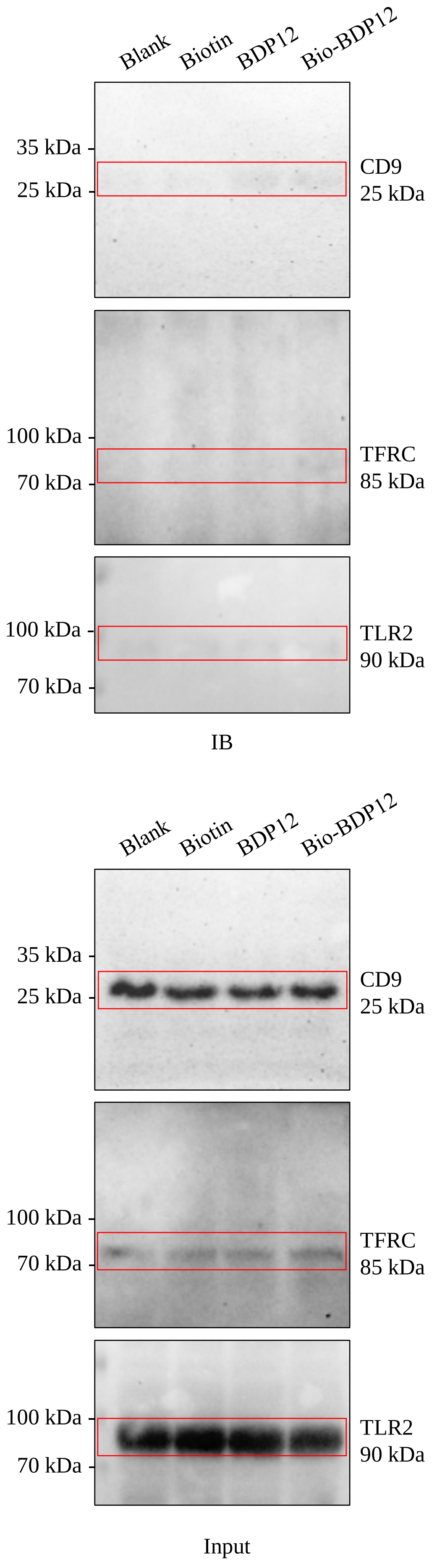

Supplement: Supplementary file 2 — Supporting File 2: advs74969‐sup‐0002‐Data.pdf. [file ADVS-13-e21164-s002.pdf]
